# Global trends and future predictions of gastrointestinal ulcers in youth

**DOI:** 10.3389/fpubh.2025.1511050

**Published:** 2025-07-18

**Authors:** Kun He, Shicai Ye, Yanqi Kou, Shenshen Du, Weinan Yuan, Lei Ge, Yuan Tian, Botao Luo, Yanping Ha, Liping Zhan, Ruyin Ye, Yujie Huang, Bingbing Li, Biao Nie, Yuping Yang

**Affiliations:** ^1^Department of Gastroenterology, Affiliated Hospital of Guangdong Medical University, Guangdong Medical University, Zhanjiang, China; ^2^Department of Gastroenterology, The First Affiliated Hospital of Jinan University, Jinan University, Guangzhou, China; ^3^The First Affiliated Hospital, and College of Clinical Medicine of Henan University of Science and Technology, Luoyang, China; ^4^Department of Gastroenterology, Yellow River Hospital Affiliated of Henan University of Science and Technology, Sanmenxia, China; ^5^Department of Gastrointestinal Surgery, Affiliated Hospital of Guangdong Medical University, Guangdong Medical University, Zhanjiang, China; ^6^Department of Pathology, Guangdong Medical University, Zhanjiang, China

**Keywords:** gastrointestinal ulcers, global burden, age-standardized rates, ARIMA model, DALYs

## Abstract

**Background:**

By analyzing past disease trends and comparing two time series statistical models, we can predict the global burden of gastrointestinal ulcers in specific populations of adolescents and young adults aged 10–24. This prediction can provide important references for optimizing prevention and control strategies in healthcare systems.

**Methods:**

We collected data on prevalence, incidence, disability-adjusted life years (DALYs), and mortality for specific age groups between 10 and 24 years from 1990 to 2019. The data were then stratified by age, gender, and economic development level. We applied decomposition analysis and frontier analysis, and compared the performance of two statistical prediction models. We used the best-performing model to predict changes in each indicator.

**Results:**

In 2019, there were 958,842 (95% uncertainty interval [UI]: 639,698–1,371,106) prevalent cases, 407,850 (95% UI: 260,513–577,751) incident cases, 363,862 (95% UI: 309,793–422,230) DALY cases, and 4,404 (95% UI: 3,826–5,042) deaths globally, all showing an increasing trend compared to 1990. However, the age-standardized prevalence rate (ASPR), age-standardized incidence rate (ASIR), age-standardized DALY rate (ASDR), and age-standardized death rate (ASDER) all decreased from 1990 to 2019, with reductions of 6.6, 3.8, 50.86, and 53.8%, respectively. The estimated annual percentage change (EAPC) for these metrics was −0.59 (95% confidence interval [CI]: −0.73 to −0.46), −0.41 (95% CI: −0.51 to −0.31), −2.81 (95% CI: −2.96 to −2.66), and −3.1 (95% CI: −3.28 to −2.92), respectively. The ARIMA model, identified as the most accurate prediction model, suggests that by 2050, the burden of gastrointestinal ulcers in this age group will significantly decrease compared to 2019. Over the next 30 years, the global number of incident cases will initially rise before stabilizing, prevalent cases will fluctuate, and both DALYs and deaths will decline. ASPR, ASIR, ASDR, and ASDER will continue to decrease, with the most notable reductions in ASDR and ASDER.

**Conclusion:**

In 2019, the global burden of gastrointestinal ulcers showed significant increases in prevalent and incident cases, DALYs, and deaths compared to 1990. However, when adjusted for age, the prevalence rate (ASPR), incidence rate (ASIR), DALY rate (ASDR), and death rate (ASDER) all demonstrated substantial reductions, indicating improvements in management and prevention. The ARIMA model, identified as the most accurate, projects a significant decline in the burden of gastrointestinal ulcers for this age group by 2050, compared to 2019. While the raw numbers of cases and mortality have risen, age-standardized rates have decreased, reflecting advancements in healthcare strategies. These findings emphasize the need for continued focus on preventive measures and healthcare optimization to further reduce the global burden of gastrointestinal ulcers. The projected decline highlights the potential effectiveness of current strategies and offers a positive outlook for future management.

## Introduction

Peptic ulcer disease (PUD) is characterized by disruptions in the gastrointestinal mucosa caused by gastric acid secretion or pepsin (1). Most PUD cases are attributed to *Helicobacter pylori* (*H. pylori*)-associated PUD and nonsteroidal anti-inflammatory drug (NSAID)-associated PUD (2). Over 90% of duodenal ulcers are closely linked to *H. pylori* infection (3). The development of infection-related PUD is a complex process involving *H. pylori* infection, subsequent inflammation, and mucosal damage. Numerous studies have confirmed the negative impact of COX-1 inhibitors on the gastrointestinal tract, leading to damage in the stomach and small intestine (4). Currently, the combined use of antibiotics and proton pump inhibitors (PPIs) is effective in eradicating *H. pylori* for treating infection-related PUD. However, the global increase in antibiotic resistance, coupled with the rise in respiratory diseases following the COVID-19 pandemic, has led to widespread NSAID and antipyretic use, which may contribute to an increase in gastrointestinal ulcers. These two factors’ fluctuations may drive changes in the burden of gastrointestinal ulcer diseases. Additionally, a retrospective meta-analysis by Ciociola et al. (5) reviewing six U.S. clinical trials found that approximately 27% of ulcer patients had neither *H. pylori* infection nor NSAID-induced ulcers, referred to as “idiopathic ulcers.” The occurrence of idiopathic ulcers is believed to result from the interaction of multiple pathogenic factors, including stress, hypersecretion of acid, and gastrinomas (6). Contemporary issues, such as poor lifestyle habits, stress, and urbanization, are also closely related to PUD occurrence (7), making it a significant challenge to control the burden of peptic ulcer disease. Reducing *H. pylori* infection and managing NSAID or aspirin use remain key strategies for lowering the risk of gastric and duodenal ulcers (8, 9). PUD primarily affects the stomach and proximal duodenum, with a lifetime prevalence of 5–10% in the general population of Western countries (10), and an annual incidence of 0.1–0.3% (11). Although the number of hospitalizations due to peptic ulcer bleeding has steadily decreased globally, the mortality rate remains stable at 5–10% (12). The most severe complication is gastrointestinal perforation, usually presenting as sudden severe upper abdominal pain. Depending on the patient’s age and complications, the mortality rate following perforation can reach up to 20% (13). PUD can also lead to other conditions. For example, a recent population study found that the diagnosis of mood disorders is associated with an increased risk of subsequent PUD/gastritis (14). Compared to middle-aged and older adults, adolescents and young adults typically have milder symptoms and lower risks of severe complications, often resulting in underdiagnosis and undertreatment. However, neglecting these issues can lead to serious long-term consequences. Despite this, research on the global burden of gastrointestinal ulcers in adolescents and young adults is limited, highlighting the need to assess the trends in the burden of gastrointestinal ulcers in the 10–24 age group from 1990 to 2019, based on data from the Global Burden of Disease, Injuries, and Risk Factors (GBD) study at global, regional, and national levels.

## Methods

We analyzed the 2019 Global Burden of Disease (GBD) study using repeated cross-sectional data from the Global Health Data Exchange (GHDx). This dataset covers the global burden of 369 diseases and injuries across 204 countries and regions from 1990 to 2019, including gastrointestinal ulcers. According to the Global Biodiversity Development Project’s definition, data on gastrointestinal ulcers in the 10–24 age group were collected from 21 regions with similar geographic and biological characteristics. In this study, adolescents are defined as those aged 10–19, and young adults as those aged 20–24. The GBD 2019 also calculated the Sociodemographic Index (SDI) for each country, a composite measure reflecting social and economic factors that influence health outcomes. The SDI is the geometric mean of the total fertility rate for individuals under 25, the mean years of education for those aged 15 and older, and the lag-distributed income per capita index, on a scale of 0 to 1, where 0 represents the lowest education, income, and highest fertility. The SDI is divided into five quintiles: low, low-middle, middle, middle-high, and high. Incident cases, prevalent cases, deaths, mortality rate, incidence rate, and prevalence rate were directly extracted from GBD 2019, with all rates per 100,000 people. The 95% uncertainty intervals (UI) are based on the 25th and 975th values from 1,000 estimates using the GBD algorithm. The Institutional Review Board of the First Affiliated Hospital of Jinan University determined that approval was not required as the study used publicly available data. This study adhered to the Guidelines for Accurate and Transparent Health Estimates Reporting (15, 16).

### Statistical analysis

The age-standardized rates (ASR), mortality rates, percentages, and DALYs were extracted from the GBD 2019 study. Crude incidence rates (e.g., prevalence, incidence, mortality) are fundamental indicators for tracking disease trends. However, differences in population age structure can cause heterogeneity in the burden of gastrointestinal ulcers across groups. To ensure comparability, we adjusted the crude rates by age structure to obtain age-standardized rates (ASR). The age-standardized prevalence rate (ASPR), age-standardized incidence rate (ASIR), age-standardized DALY rate (ASDR), and age-standardized death rate (ASDER) were then used to estimate the burden of gastrointestinal ulcers in adolescents aged 10–24.

We used a generalized linear model with a Gaussian distribution to calculate the estimated annual percentage change (EAPC) of the prevalence rate, incidence rate, DALY rate, and death rate to quantify global disease trends. If the lower limit of the EAPC and its 95% confidence interval (CI) is > 0, the disease burden is considered to have increased. Conversely, if the upper limit of the EAPC and its 95% CI is < 0, a decreasing trend is observed.

Additionally, the ARIMA (Autoregressive Integrated Moving Average) model, a statistical tool for forecasting and analyzing time series data, was employed in our study. ARIMA plays a crucial role by predicting disease trends, which aids in public health policy-making and resource allocation. The model can also predict missing data based on existing data, improving dataset completeness and enhancing the accuracy of the research. ARIMA helps identify outliers, ensuring data quality and accurate trend interpretation. Thus, it provides in-depth insights into disease trends and enhances analytical precision, making it valuable for global disease burden research.

The Exponential Smoothing (ES) model, another time series forecasting tool, was also used in GBD analysis. The ES model is well-suited for predicting disease trends and evaluating public health intervention.

## Results

### Prevalence, incidence, DALYs and death of the global peptic ulcer burden from 1990 to 2019

In 2019, there were 958,842 (95% UI: 639,698–1,371,106) prevalent cases of gastrointestinal ulcers globally, with an age-standardized prevalence rate (ASPR) of 51.5 per 100,000 (95% UI: 34.36–73.64), reflecting a 6.6% decrease since 1990. Additionally, 407,850 (95% UI: 260,513–577,751) new cases were reported, with an age-standardized incidence rate (ASIR) of 21.91 per 100,000 (95% UI: 13.99–31.03), a 3.8% decline since 1990. The number of disability-adjusted life years (DALYs) related to gastrointestinal ulcers was 363,862 (95% UI: 309,793–422,230), with an age-standardized DALY rate (ASDR) of 19.54 (95% UI: 16.64–22.68), showing a significant 50.68% reduction since 1990. There were 4,404 (95% UI: 3,826-5,042) deaths, with an age-standardized death rate (ASDER) of 0.24 (95% UI: 0.21–0.27), a 53.8% decrease from 1990.

In 2019, the highest ASPR, ASIR, ASDR, and ASDER for gastrointestinal ulcers were observed in Kiribati, with 232.47 (95% UI: 172.26–303.4), 69.17 (95% UI: 50.14–93.99), 121.9 (95% UI: 57.99–201.99), and 1.58 (95% UI: 0.65–2.75) per 100,000, respectively. Conversely, the lowest ASPR and ASIR were found in Costa Rica, with 2.75 (95% UI: 1.42–4.71) and 1.24 (95% UI: 0.65–2.13) per 100,000, respectively. Andorra had the lowest ASDR at 0.69 per 100,000 (95% UI: 0.43–1.02), while ASDER was zero in multiple countries.

From 1990 to 2019, the Syrian Arab Republic experienced the largest increases in ASPR (44.8%) and ASIR (40.8%), while Zimbabwe saw the greatest rises in ASDR (39%) and ASDER (42%). In contrast, Bhutan saw a 71% decrease in both ASPR and ASIR, Spain had the largest decrease in ASDR (85.5%), and ASDER dropped to zero in several countries (Supplementary Tables 1–4).

Regions with incidence rates exceeding 30% in 2019 included parts of Africa, Europe, and East Asia, while most of North America had rates between 10 and 20%. Areas with the highest prevalence rates were in Africa, Central Asia, and South Asia, with some regions surpassing 120%, while regions with the lowest prevalence rates, including most of the Americas and Europe, ranged from 0 to 30%. Mortality rates were highest in Africa and lowest in most of North America and Europe, where rates were under 0.2. DALY rates were highest in sub-Saharan Africa and parts of South America, and lowest in most of North America and Europe.

**Figure 1:**
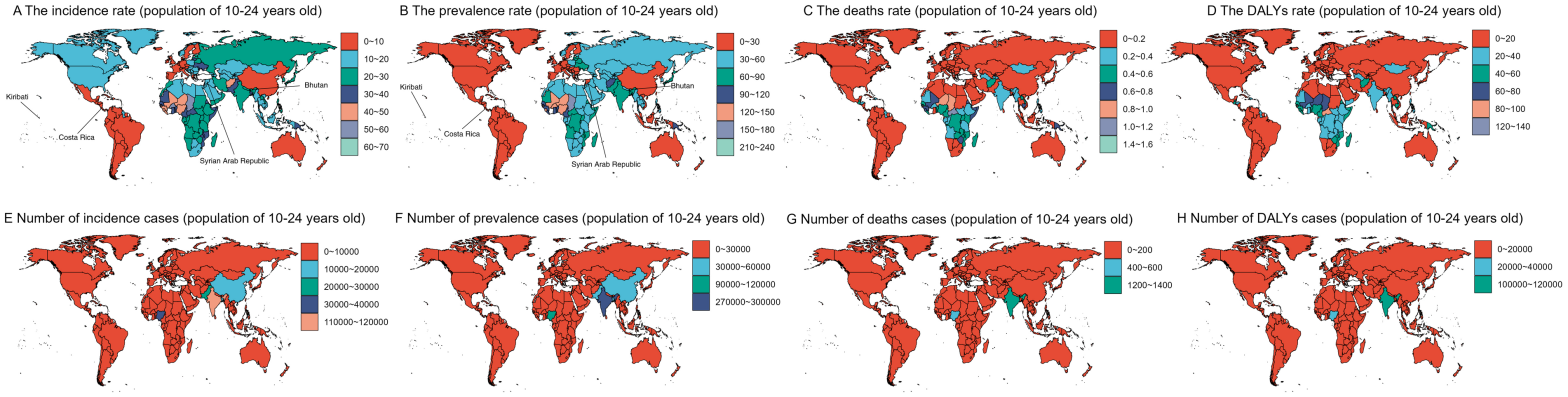
World maps displaying data for the population aged 10-24 years. Panels **(A–D)** show the incidence rate, prevalence rate, death rate, and disability-adjusted life years (DALY) rate, respectively, using color gradients to represent different value ranges. Panels **(E–H)** present the number of incidence cases, prevalence cases, death cases, and DALY cases. Kiribati: From 1990 to 2019, it had the highest prevalence (ASPR: 232.47 cases [95% UI: 172.26–303.4] per 100,000) and incidence (ASIR: 69.17 cases [95% UI: 50.14–93.99] per 100,000) rates in the world. Costa Rica: From 1990 to 2019, it had the lowest ASPR (2.75 cases [95% UI: 1.42–4.71]) and ASIR (1.24 cases [95% UI: 0.65–2.13]) globally. Syrian Arab Republic: Showed the fastest growth in both indicators—ASPR increased by 44.8%, and ASIR by 40.8% during the same period. Bhutan: Experienced the largest decline in both indicators—ASPR and ASIR both decreased by 71% from 1990 to 2019.

In terms of the number of incident cases, prevalent cases, deaths, and DALYs, populous countries like India and China had relatively high levels. However, India had a globally high number of deaths and DALYs, which warrants attention from local health authorities and the national government. Overall, the disease burden among the 10–24 age group shows significant global disparities, with high levels in some African and Asian countries, and lower levels in many North American, European, and East Asian countries (Figure 1).

### Peptic ulcer burden in 44 GBD among the 44 GBD regions

Among the 44 GBD regions, the highest age-standardized prevalence rate (ASPR) and incidence rate (ASIR) for gastrointestinal ulcers were in Western Sub-Saharan Africa, with 123.93 cases per 100,000 (95% UI: 85.5–171.52) and 46.7 cases per 100,000 (95% UI: 31.68–65.42), respectively. The highest age-standardized death rate (ASDR) and excess death rate (ASDER) were in Oceania, at 55.86 per 100,000 (95% UI: 39.46–81.64) and 0.72 (95% UI: 0.48–0.98), respectively. Central Latin America had the lowest ASPR and ASIR, at 7.86 (95% UI: 4.95–11.89) and 3.23 (95% UI: 1.99–4.77) per 100,000, respectively. Australasia had the lowest ASDR and ASDER, with 0.81 (95% UI: 0.56–1.17) and 0 (0–0.01), respectively.

The largest increases in ASPR and ASIR were observed in High-income Asia Pacific and Central Europe, with increases of 4.7 and 5.8%, respectively. However, ASDR and ASDER decreased across all regions. The country with the highest reduction in ASPR saw a 36.8% decrease, while Latin America & Caribbean – WB had the highest drop in ASIR at 45%. The greatest decrease in ASDR occurred in East Asia (77%), while the largest decrease in ASDER was in Australasia, with a 100% reduction (Supplementary Tables 1–4).

**Figure 2:**
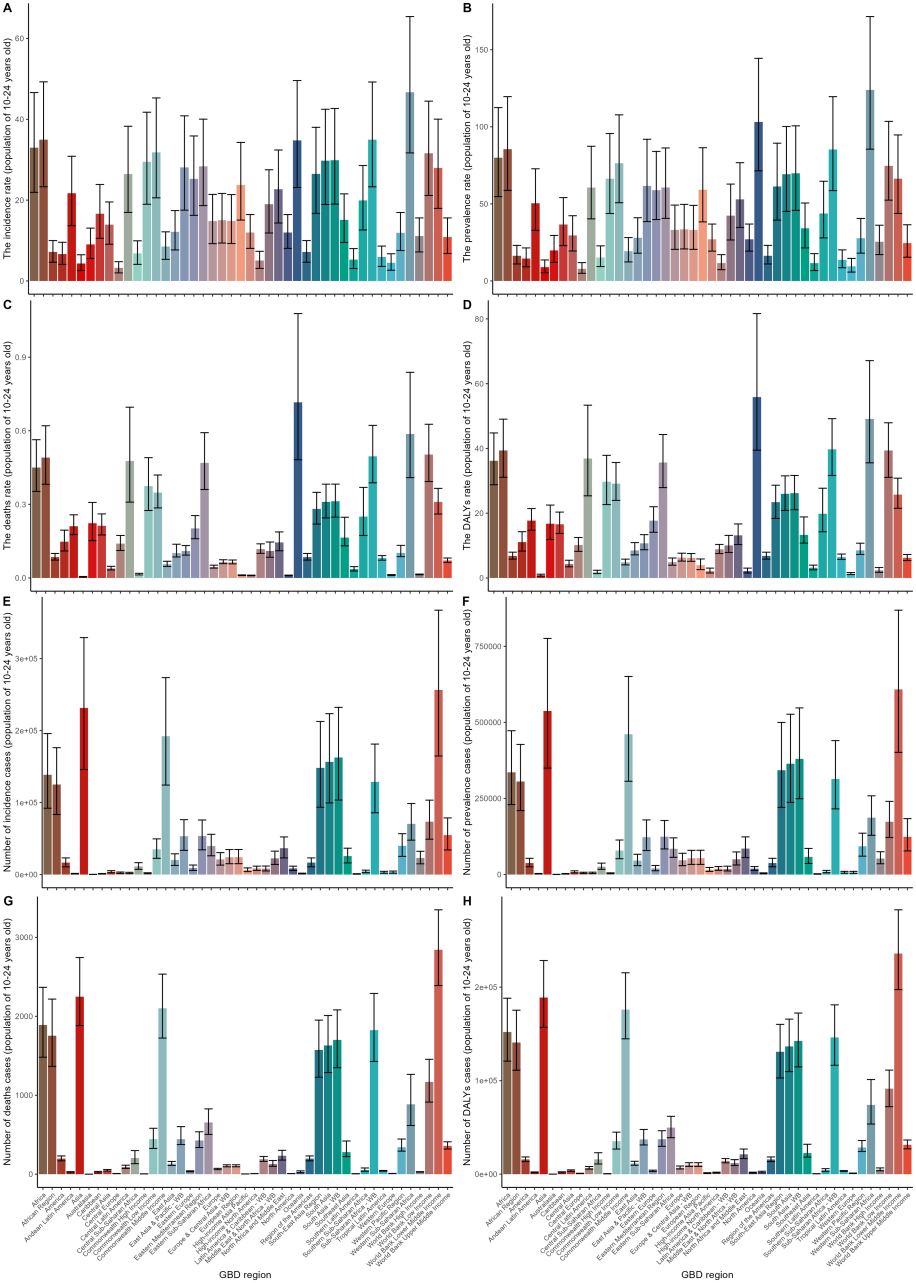
This figure shows the regional distribution of gastrointestinal ulcer burden among individuals aged 10–24 years across Global Burden of Disease (GBD) regions. Panels **A–D** present age-standardized rates of incidence, prevalence, deaths, and DALYs (disability-adjusted life years) per 100,000 population;Panels **E–H** display the corresponding number of incident cases, prevalent cases, deaths, and DALYs. Each bar represents a GBD region, and black error bars indicate the 95% uncertainty intervals.

In 2019, Western Sub-Saharan Africa had the highest incidence and prevalence rates, while Oceania had the highest death and DALY rates. The highest incidence, prevalence, death, and DALY cases were in the World Bank lower middle-income countries and Asia (Figures 2A–H). In regions with limited healthcare systems, notable gender disparities were observed. Females had significantly higher age-standardized prevalence rates (ASPR) of 76.17 per 100,000 (95% UI: 50.85–108.33) compared to males at 53.18 per 100,000 (95% UI: 36.24–75.44). A similar pattern was seen in the age-standardized incidence rates (ASIR), with females at 31.49 per 100,000 (95% UI: 20.85–42.53) and males at 23.72 per 100,000 (95% UI: 15.91–31.83). However, in East Sub-Saharan Africa, males had the highest age-standardized death rates (ASDR) at 33.93 per 100,000 (95% UI: 20.91–48.16) and excess death rates (ASDER) at 0.91 (95% UI: 0.60–1.20), showing a unique gender pattern (Supplementary Figures 1A,B).

In terms of age distribution, while most regions showed an increasing trend with age, West Africa, Oceania, and Sub-Saharan Africa exhibited distinct patterns, with ASPR peaking in the 15–19 age group and ASIR peaking in the 10–14 age group. Notably, in East Sub-Saharan Africa, the 20–24 age group had the highest disease burden, with an ASDER of 1.82 (95% UI: 1.20–2.32) and an ASDR of 127.72 per 100,000 (95% UI: 86.22–163.15) (Supplementary Figures 2A,B).

Among major populous regions, significant gender disparities were seen in the number of incidence cases, prevalence cases, deaths, and DALYs, particularly in high-density population areas.

### Peptic ulcer burden in SDI regions

Among all SDI regions, Low SDI countries had the highest rates of ASPR, ASIR, ASDR, and ASDER for gastrointestinal ulcers, with 82.81 (95% UI: 57.73–115.49), 34.01 (95% UI: 22.73–47.77), 37.71 (95% UI: 31.16–44.64), and 0.47 (95% UI: 0.38–0.56) per 100,000, respectively. In contrast, High SDI countries had the lowest rates, with 27.25 (95% UI: 18.28–38.69), 11.8 (95% UI: 7.62–16.6), 2.38 (95% UI: 1.75–3.36), and 0.01 (95% UI: 0.01–0.02) per 100,000. Across all SDI regions, ASPR, ASIR, ASDR, and ASDER showed a decreasing trend, with the largest declines in ASPR, ASIR, and ASDR in Low-middle SDI countries (26.6, 21.5, and 65.6%, respectively). ASDER decreased the most in High SDI countries, by 83.3%. The reductions in ASPR and ASIR were more pronounced in Low SDI than in Low-middle SDI regions, while ASDR declined faster in Low-middle SDI countries, highlighting a lag in treatment capabilities in Low SDI regions (Supplementary Tables 1–4).

In 2019, the incidence rate, prevalence rate, mortality rate, and DALY rate all increased as economic levels decreased. The number of incidence cases, prevalence cases, deaths, and DALYs was generally higher in Low-middle SDI countries compared to Low SDI regions, with these regions leading in deaths and DALYs (Supplement Figures 3A–H). Integrating the socioeconomic context, the gray lines represent the expected rates for ASPR, ASIR, ASDR, and ASDER based on the 2019 SDI values. The analysis shows that disease burden indicators generally improve as SDI levels increase. However, Kiribati and Vanuatu have significantly higher disease burden indicators than expected, suggesting substantial room for improvement in these regions (Supplementary Figures 4A–D).

### Peptic ulcer burden by gender

In 2019, gender-specific data for digestive ulcers revealed that females had higher age-standardized prevalence rates (ASPR), incidence rates (ASIR), and disability-adjusted life years (ASDR), with values of 60.27 (95% UI: 40.48–85.08), 24.5 (95% UI: 16–34.83), and 20.26 (95% UI: 16.78–24.33), respectively. However, the age-standardized death rate (ASDER) was higher in males, at 0.24 (95% UI: 0.21–0.29). Both genders showed declines in these indicators, but the reductions in ASPR, ASIR, and ASDR were more pronounced in males, at 10.5, 9.3, and 51.1%, respectively (Supplementary Tables 1–4). In contrast, females experienced a larger decrease in ASDER, at 55.7%. Regarding gender differences in digestive ulcers, incidence rates, number of incident cases, prevalence rates, and number of prevalent cases were higher in females than in males. While there was no significant difference in mortality rates and death counts between genders, males had higher rates. The DALY rate and DALY count were also slightly higher in females, but the difference was not significant (Supplementary Figures 5A–H).

### Peptic ulcer burden in EAPC

**Figure 3:**
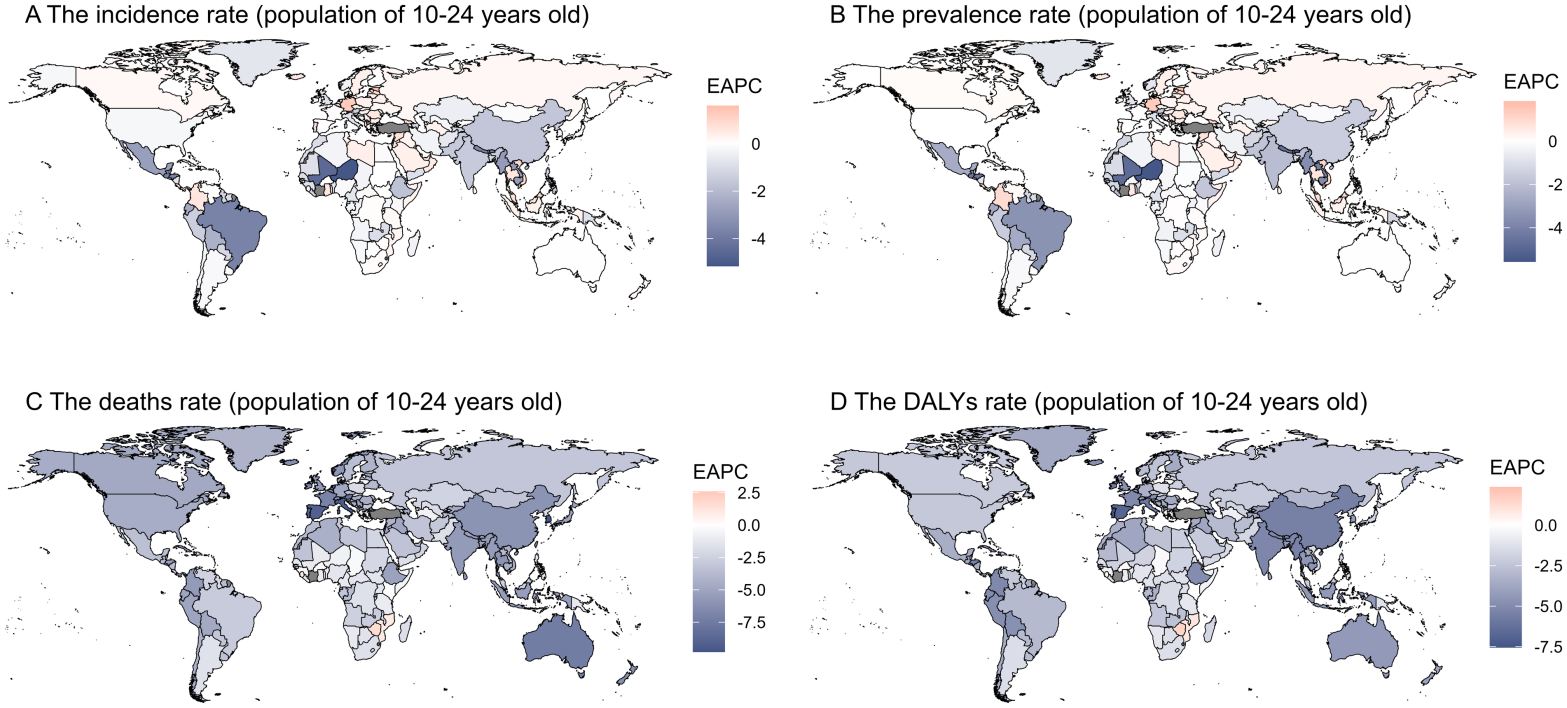
This figure shows the Estimated Annual Percentage Change (EAPC) in age-standardized rates of gastrointestinal ulcers among individuals aged 10–24 years from 1990 to 2019, with panels **A–D** presenting the EAPC for incidence, prevalence, mortality, and DALY rates, respectively; blue indicates a decreasing trend, red indicates an increasing trend, highlighting regional differences in temporal changes in disease burden.

Globally, the burden of digestive ulcers among individuals aged 10 to 24 has declined across 204 countries. The EAPC values for prevalence, incidence, DALYs, and mortality rates decreased by −0.59 (95% CI: −0.73 to −0.46), −0.41 (95% CI: −0.51 to −0.31), −2.81 (95% CI: −2.96 to −2.66), and −3.1 (95% UI: −3.28 to −2.92), respectively. Among the 44 GBD regions, Central Europe saw the highest increases in EAPC values for prevalence (0.29; 95% CI: 0.18–0.41) and incidence (0.33; 95% CI: 0.24–0.42). Sub-Saharan Africa had the largest increase in DALYs (0.33; 95% CI: −0.17-0.83), while Southern Sub-Saharan Africa experienced the highest rise in ASDER (0.37; 95% UI: −0.19-0.94). In contrast, Tropical Latin America showed the largest decreases in prevalence (−3.39; 95% CI: −3.69 to −3.09) and incidence (−3.63; 95% CI: −3.97 to −3.29), and East Asia had the greatest decline in DALYs (−5.29; 95% CI: −5.44 to −5.14). Australasia exhibited the largest decrease in ASDER (−7.16; 95% UI: −7.72 to −6.59). In SDI regions, both prevalence and incidence rates decreased, with the most significant reductions in low SDI regions (−1.54; 95% CI: −1.76 to −1.31) and low-middle SDI regions (−1.25; 95% CI: −1.42 to −1.07). Low-middle SDI countries saw the largest decrease in DALYs (−4.18; 95% CI: −4.47 to −3.9), while high SDI regions had the greatest reduction in mortality (−5.42; 95% UI: −5.65 to −5.19). By gender, EAPC values for prevalence, incidence, DALYs, and mortality showed a downward trend, with females experiencing more significant declines in prevalence (−0.64; 95% CI: −0.82 to −0.46), incidence (−0.37; 95% CI: −0.51 to −0.22), DALYs (−2.89; 95% UI: −3.1 to −2.68), and mortality (−3.3; 95% UI: −3.56 to −3.04). Males had a more pronounced decrease in incidence (−0.45; 95% CI: −0.52 to −0.38) (Supplementary Tables 1–4). The EAPC values for prevalence and incidence were negative in most countries, particularly in parts of the Americas and Northern Africa, suggesting a significant decline in incidence. While mortality rates among adolescents remained stable in most regions, some areas in the Americas showed a positive EAPC value, indicating an increase in adolescent mortality. In contrast, negative EAPC values in parts of the Americas and Asia indicated a reduction in health loss due to the disease during the study period. However, some regions saw positive EAPC values, reflecting an increase in health loss (Figure 3).

### Temporal trends of digestive ulcer incidence: insights from overall, socio-demographic index (SDI), and gender stratifications (0–24 years)

**Figure 4:**
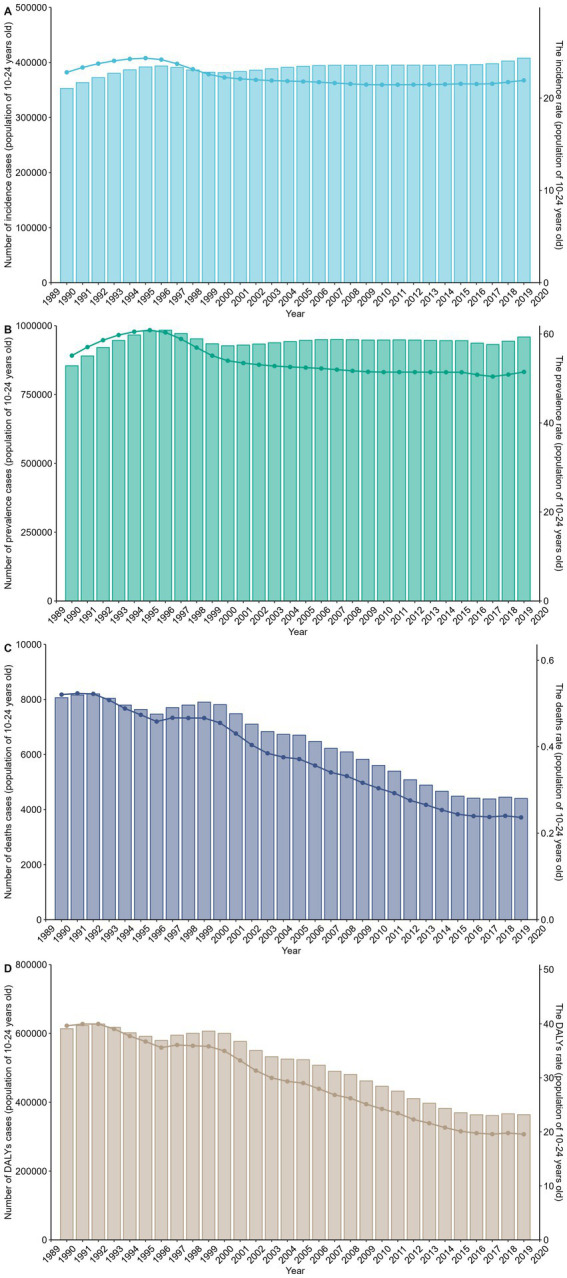
This figure shows the trends in the burden of gastrointestinal ulcers among individuals aged 10–24 years from 1990 to 2019, with panels A–D displaying the number of cases and age-standardized rates for incidence **(A)**, prevalence **(B)**, deaths **(C)**, and DALYs **(D)**; rates are per 100,000 population, highlighting an overall increase in case numbers alongside a decline in standardized rates over time.

The overall time trends from 1990 to 2019 describe changes in disease incidence and prevalence, showing a generally stable trend with slight fluctuations but no significant increase or decrease. Similarly, the number of prevalent cases has shown a relatively stable trend, akin to the number of incident cases. Regarding mortality numbers and rates, we can observe a consistent decline year by year, reflecting improvements in mortality rates. The trends for DALYs and DALYs rates also show a yearly decreasing trend (Figure 4). Based on the time descriptions of SDI regions, we can see that high SDI regions generally outperform low SDI regions in all aspects. The incidence and prevalence rates in high SDI regions show a decreasing trend, while in low SDI regions, they are relatively stable or slightly increasing. Mortality rates and DALYs in all SDI classifications are on a declining trend, but the decline is more significant in low SDI regions (Supplementary Figure 6). These trend lines illustrate the changes in incidence (A), prevalence (B), mortality rates (C), and DALYs rates (D) over time by gender. It is noteworthy that females generally have higher values in all indicators, particularly in incidence and prevalence rates. The differences in mortality and DALYs rates between males and females are not as pronounced as in incidence and prevalence rates, but males typically show higher values (Supplementary Figure 7).

### Frontier analysis of peptic ulcer burden

**Figure 5:**
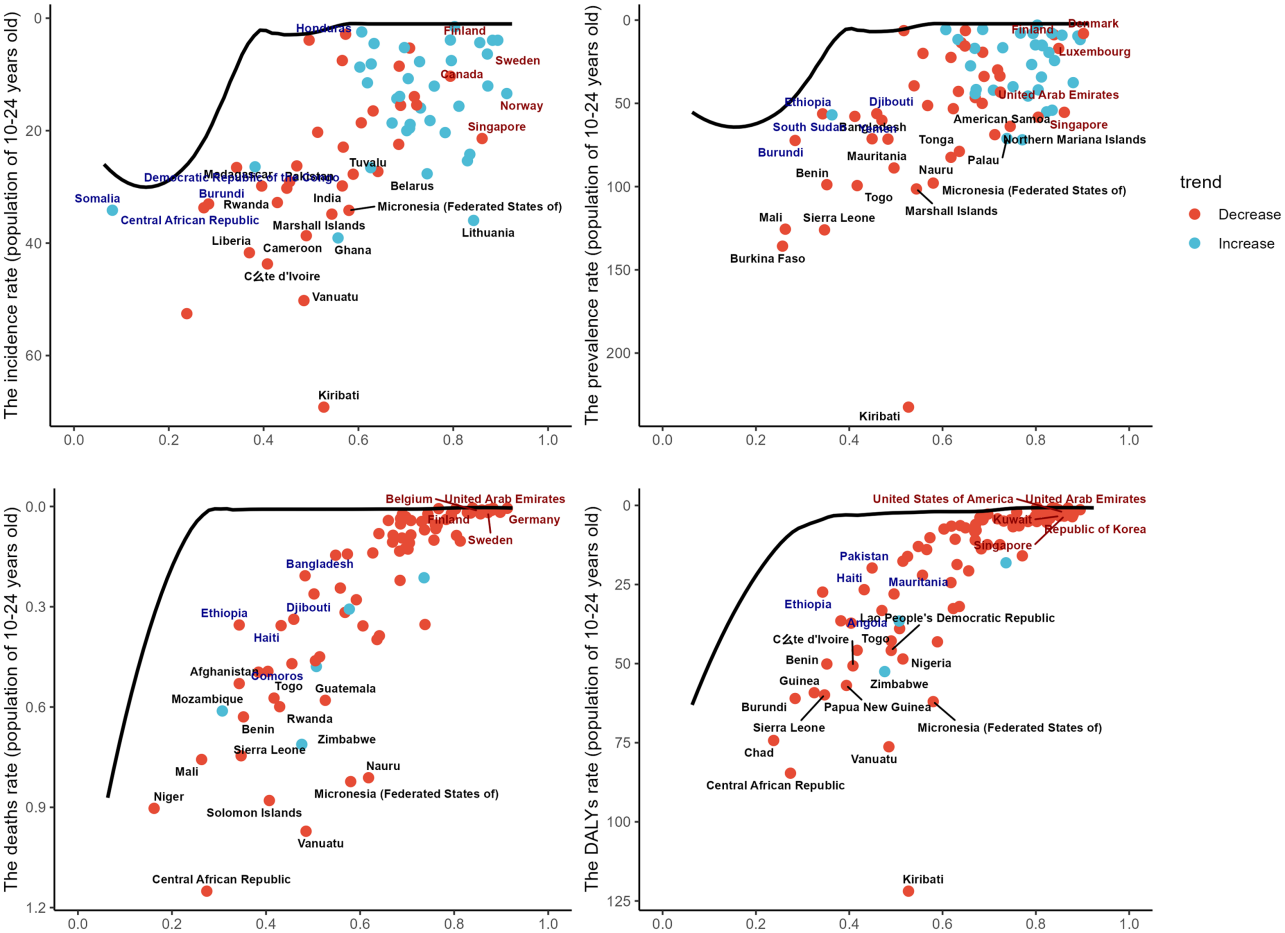
This figure shows the relationship between the Sociodemographic Index (SDI) and the burden of gastrointestinal ulcers among individuals aged 10–24 years across countries, with panels **A–D** presenting age-standardized rates of incidence, prevalence, mortality, and DALYs per 100,000 population; each dot represents a country, where red indicates a decreasing trend and blue indicates an increasing trend, and the black line represents the frontier curve, reflecting differences and distributions in disease burden relative to development level.

The boundary represents the countries or regions with the lowest disease burden, based on their SDI. The effective distance is the gap between a country’s observed burden and the achievable burden according to its SDI. A significant effective difference suggests unrealized opportunities for improvement, such as reducing the incidence of digestive diseases. Using 2019 data on prevalence, incidence, DALYs, and mortality alongside SDI, we estimated the effective difference for each country or region from the boundary. The largest effective differences were found in Kiribati, Vanuatu, Burkina Faso, and the Central African Republic, which, due to their lower development levels, have relatively poor economic conditions. In contrast, countries with the smallest effective differences, such as Switzerland, the United States, and Finland, are in developed regions. This highlights that countries with lower economic levels experience a higher burden of digestive ulcers. In the figure, the black solid line represents the boundary, dots indicate countries and regions, blue dots show an increasing trend, and red dots indicate the opposite (Figure 5).

### Interpretation of gastrointestinal ulcer decomposition models

**Figure 6:**
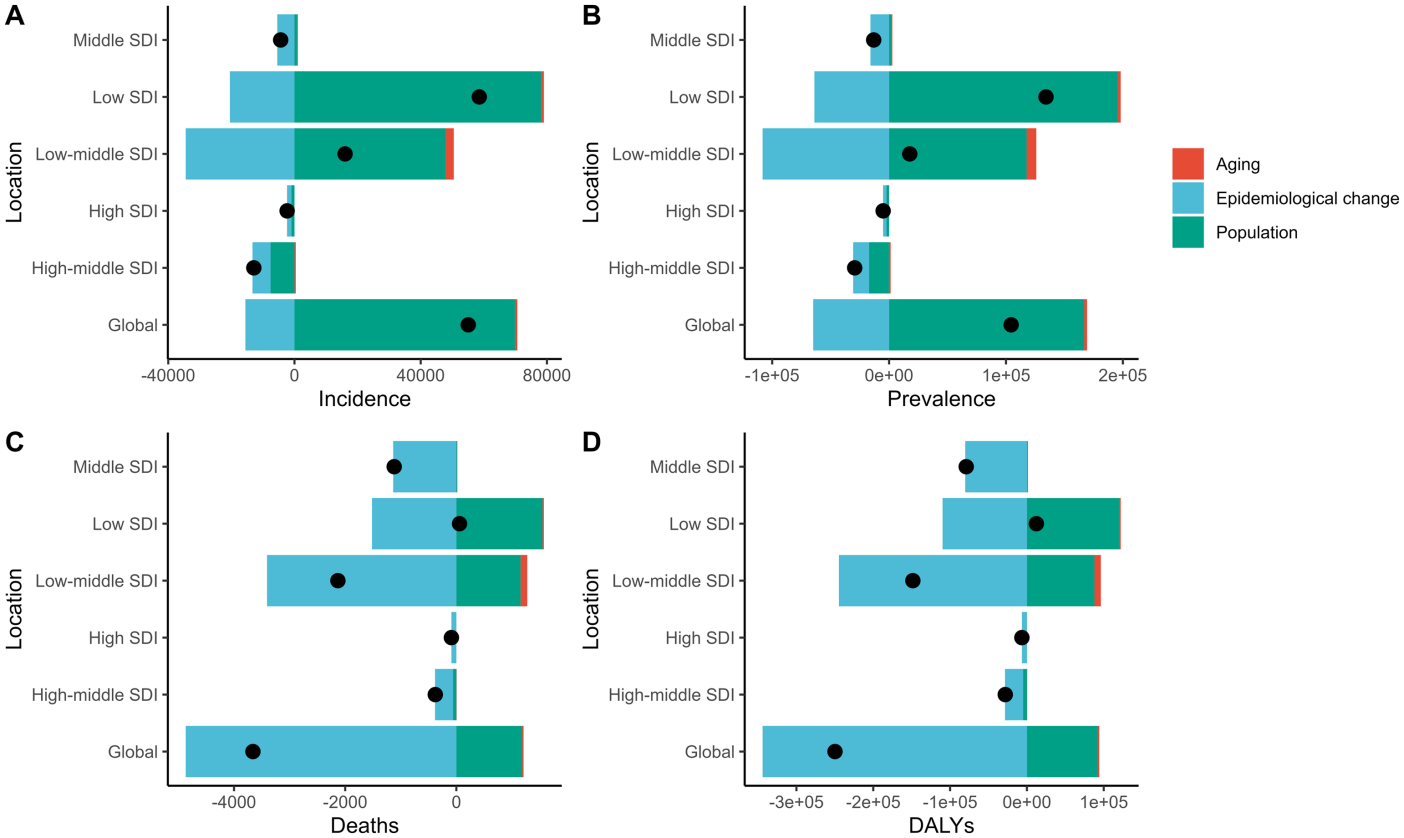
This figure illustrates the changes in the number of incident cases **(A)**, prevalent cases **(B)**, deaths **(C)**, and total DALYs **(D)** due to gastrointestinal ulcers among individuals aged 10–24 years from 1990 to 2019 at the global level and across different Sociodemographic Index (SDI) groups. The contributions of population growth (green bars), epidemiological changes (blue bars), and population aging (red bars) to these changes are shown. The black dots represent the total net changes, highlighting the relative contributions of each factor to the disease burden in different SDI regions.

This figure shows the decomposition of disease metrics across Socio-Demographic Index (SDI) regions in the Global Burden of Disease (GBD) study. The main factors analyzed are aging (red), epidemiological changes (blue), and population growth (green). The four subplots depict changes in incidence (A), prevalence (B), mortality (C), and Disability-Adjusted Life Years (DALYs) (D). Incidence rates have risen significantly due to population growth, particularly in low and low-middle SDI regions. The effect of aging on incidence is minor in high and high-middle SDI regions. Prevalence changes are mainly driven by population growth, with all SDI regions showing an upward trend. Epidemiological changes particularly influence prevalence in low and low-middle SDI regions. Mortality changes are primarily driven by epidemiological shifts, especially in low and low-middle SDI regions, while population growth has a smaller effect on mortality in high SDI regions and globally. DALY changes are mainly driven by epidemiological factors, showing a decline across all SDI regions, especially in low and low-middle SDI areas. The impact of population growth on DALYs is minimal in high SDI regions. Overall, population growth is the primary driver of the increasing disease burden in various SDI regions, while epidemiological changes help reduce mortality and DALYs. Aging has a greater impact in high SDI regions but a relatively small effect globally. These findings suggest that low SDI regions should focus on strategies addressing population growth and epidemiological changes to mitigate disease burden (Figure 6).

### Comparison of ARIMA and ES models for disease burden forecasting: a time series analysis approach

**Figure 7:**
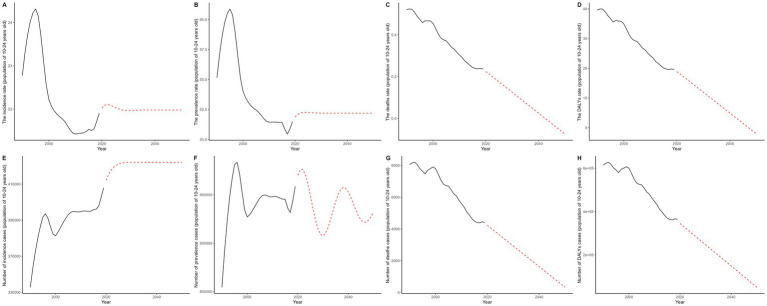
This figure illustrates the historical trends (1990–2019) in the burden of gastrointestinal ulcers among individuals aged 10–24 years and projects changes through 2050 using the ARIMA model; panels **A–D** show age-standardized incidence, prevalence, mortality, and DALY rates per 100,000 population, while panels **E–H** display the corresponding numbers of cases, with black solid lines representing observed data and red dashed lines indicating ARIMA-based projections.

ARIMA (Auto-Regressive Integrated Moving Average) and ES (Exponential Smoothing) are widely used time series forecasting methods. In the context of the Global Burden of Disease (GBD) database, they provide reliable predictions for trends in various health indicators. In this study, we applied both models to forecast the future trends of eight data points and compared their predictive performance. The ARIMA model outperformed the ES model in terms of smaller ME, RMSE, MAE, and MASE, indicating a better fit. For AIC and BIC values, the ARIMA model’s values were below 500, while the ES model’s values exceeded 500, further supporting the ARIMA model’s superiority. Residual checks showed that both models passed the white noise test. The ARIMA model’s residuals followed a normal distribution, with a Shapiro test *p*-value of less than 0.05, whereas the ES model’s *p*-value was greater than 0.05 (Supplementary Table 5). In summary, the ARIMA model proved to be superior. Regarding long-term trends, solid lines in the figures represent actual observed values, and dashed lines represent model-predicted future trends. In most cases, the forecasted trends suggest a gradual decline in disease burden indicators from 2020 (Figure 7).

## Discussion

This study analyzes the burden and trends of gastrointestinal ulcers among adolescents and young adults aged 10 to 24 years across 204 countries and regions from 1990 to 2019. Compared to 1990, both the incidence and prevalence of gastrointestinal ulcers increased by 2019, while the disability-adjusted life years (DALYs), mortality, and corresponding rates decreased. From 1990 to 2019, age-standardized mortality rate (ASPR) and age-standardized incidence rate (ASIR) declined by 6.6 and 3.8%, respectively, while age-standardized DALYs (ASDR) and age-standardized death rate (ASDER) decreased by 50.68 and 53.8%. The rapid decline in ASDR and ASDER is primarily attributed to advances in treatment, particularly in managing severe complications through techniques such as endoscopy, proton pump inhibitors (PPIs), and interventional embolization. Additionally, screening and treatment for high-risk populations have contributed to the prevention of severe complications (17). However, the years of disability and death caused by these diseases remain significantly high in low- and middle-income countries. Among the four burden indicators, the disparities are particularly evident in high population density and low-income regions. For example, India has the highest disease burden globally. From a GBD regional perspective, economically underdeveloped but densely populated areas in Africa and Asia account for the majority of cases. India and similar regions require more proactive health policies to reduce the disease burden among youth. Moreover, in some Pacific island nations like Kiribati, the values for ASPR, ASIR, ASDR, and ASDER in 2019 were the highest globally. This could be due to two factors: first, a higher rate of *Helicobacter pylori* (*H. pylori*) infection in Pacific island populations, with a distinct ethnic characteristic. Research in South Auckland shows significantly higher *H. pylori* infection rates among Māori and Pacific Islanders compared to Europeans in the region (18). Data also show that overcrowding in Pacific island households contributes to a 44% *H. pylori* infection rate (19, 20). Second, a study on healthcare accessibility ranked Kiribati among the six worst countries globally (21). Despite the global burden, many countries show a declining trend, likely linked to improved *H. pylori* eradication rates and standardized use of non-steroidal anti-inflammatory drugs (NSAIDs) (22). In recent years, significant progress has been made with the PPI triple therapy for *H. pylori*-related peptic ulcer disease (PUD) (23). Overall, our study finds that gastrointestinal ulcers remain a significant global public health issue for adolescents and young adults. It is worth noting that peptic ulcer disease (PUD) may be associated with other chronic gastrointestinal disorders in certain populations, particularly Crohn’s disease, a major subtype of inflammatory bowel disease (IBD). In recent years, the incidence of Crohn’s disease has continued to rise globally, especially in countries and regions with a high Socio-Demographic Index (SDI). This trend parallels our study’s observation that PUD prevalence has not significantly declined in some high-SDI countries (24). Crohn’s disease can involve the stomach and duodenum and often presents as multiple *Helicobacter pylori*-negative ulcers that respond poorly to standard proton pump inhibitor (PPI) therapy. These characteristics increase the likelihood of misdiagnosis as refractory PUD, particularly among adolescents and young adults (25). Moreover, PUD and Crohn’s disease share several risk factors—such as smoking, dietary patterns, and genetic predisposition—suggesting a possible overlap in pathological mechanisms or mutual influence in certain individuals (26). Although their etiologies differ substantially—PUD is primarily driven by *H. pylori* infection and NSAID use, while Crohn’s disease is largely immune-mediated and characterized by chronic intestinal inflammation—the observed epidemiological overlap underscores the need for a more dynamic understanding of the gastrointestinal disease spectrum in public health strategies. In particular, failure to consider the increasing burden of IBD when analyzing PUD trends in developed regions may lead to an underestimation of the true burden of gastrointestinal disorders in adolescents and young adults.

Modern risk factors, such as the increasing global antibiotic resistance, also warrant attention. Studies show marked regional differences in antibiotic resistance, with Southern Europe and the Mediterranean having higher resistance rates than Northern Europe, and increasing clarithromycin resistance year by year (9, 27). For instance, previous studies have identified obesity, smoking, and alcohol consumption as major risk factors for gastric ulcers. Obesity is an independent predictor, while long-term alcohol consumption impairs gastric mucosal repair. Smoking not only increases the risk of ulcers but also delays healing, increases recurrence, and raises the incidence of ulcer-related complications (28–32). A 2023 study in China found that smoking rates among 15–24-year-olds increased from 8.3% in 2003 to 12.5% in 2013 (33). In the U.S. and other regions, adult obesity rates have risen from 15 to 33%, while childhood overweight rates have increased from 6% in 1980 to 19% in 2004 (34–37). Similar trends have been observed in low- and middle-income countries, contributing to the higher disease burden in these regions. Dietary habits may also influence the epidemiology of gastrointestinal ulcers. Soluble fiber in fruits and vegetables helps prevent duodenal ulcers (38). Evidence suggests that Hispanic populations consume more fiber-rich snacks, such as fruits and vegetables, compared to other groups (39, 40). This dietary habit, combined with a legume-based diet, shows significant protective effects. Modern challenges, such as life and academic pressures, along with unhealthy lifestyle habits, have also been identified as risk factors (3, 27). Regarding SDI regions, high-SDI regions have seen a significant decrease in gastrointestinal ulcer burden over the past 30 years. Middle-income countries have also seen reductions in burden indicators, but the decline in ASDR in low-income countries has been smaller. This suggests that the mortality rate from gastrointestinal ulcers in low-income countries should be a focus of health policies. Most of these countries have high *H. pylori* infection rates, For example, China has a 44% *H. pylori* seroprevalence, while South Africa and Nigeria have infection rates between 87 and 91% (41, 42). Our epidemiological trend analysis further confirms the significant impact of low- and middle-income SDI regions on prevalence. While middle-income countries have made some progress in controlling the disease burden, all indicators in low-SDI regions remain higher than in other regions, with slower rates of decline. This indicates that the burden of gastrointestinal ulcers is increasing in economically underdeveloped countries, which face various challenges such as insufficient healthcare investment, low diagnostic levels, poverty, gender inequality restricting access to healthcare, economic pressure from unhealthy dietary habits, and harsh work environments that exacerbate the disease burden. The complex interplay of multiple risk factors and disease patterns makes it more difficult to reduce the disease burden in these regions. In addition to promoting telemedicine and primary healthcare, increasing local medical investment and balancing resource distribution are crucial for reducing the disease burden. We urge governments and health organizations to strengthen support for low-SDI regions, adhering to the “Health for All” principle to reduce health disparities. Addressing the unique and diverse causes of disease burden in these regions is equally important. For example, in Pacific island nations, the ASIR for children aged 10–14 is the highest globally, which requires more attention from local governments and health institutions to youth disease patterns to reduce overall population prevalence. In South America, the low burden of gastrointestinal ulcers is also unique; for instance, Peru’s *H. pylori*-related PUD prevalence is 12%, starkly contrasting with the 60–75% prevalence in most developed countries (43). In Sub-Saharan Africa, regions with similar economic levels show high rates of gastrointestinal ulcer complications (including surgery) and mortality, reflecting lower healthcare standards (44). Disease patterns show significant regional differences; in high-income countries, *H. pylori* prevalence has declined, and NSAIDs and aspirin are the primary causes of peptic ulcers. Previous studies have also noted an increase in gastrointestinal diseases among women (45). Our research also found significant gender differences in the 10–24 age group. In some low- and middle-income regions, females have higher ASPR, ASIR, and ASDR than males. Since 1990, the decline in male indicators has been more substantial. Estrogen affects gastrointestinal function and activity (46, 47), and women are more likely than men to use NSAIDs. Nearly two-thirds of women use NSAIDs as first-line treatment during menstruation (48, 49). In some low-income countries, gender inequality limits women’s educational and economic opportunities, reducing their access to healthcare and preventive care. Malnutrition and impaired immunity also increase their risk of gastrointestinal ulcers (50, 51). This may explain why young women in low- and middle-income countries have higher rates of gastrointestinal ulcers. Recent analyses reveal significant disparities in incidence, prevalence, DALYs, mortality, and treatment outcomes across countries, indicating room for improvement. Although gastrointestinal ulcers are associated with socioeconomic and demographic factors, high-income countries are leading in disease control, reflecting a close relationship between economic status and ulcer incidence. For example, Japan has implemented proactive policies to control the disease burden, including PUD and gastric cancer screening programs, which have successfully reduced prevalence (52). Recently, Japan has focused more on adolescent screening and treatment, as *H. pylori* infection primarily occurs in childhood (53). Low-SDI countries often have larger family sizes, lower economic levels, overcrowding, poor sanitation, infection transmission between siblings, developmental delays, and malnutrition, thus creating clear family transmission chains. Given the technical, financial, and expertise requirements for treating PUD complications, effective prevention can be achieved through non-invasive screening, inclusion of *H. pylori* eradication drugs in insurance, improved living conditions, and enhanced public education. The international community should assist these countries in improving healthcare systems and increasing support for resource-poor regions. The burden of gastrointestinal ulcers should be prominently reflected in global and national health agendas. While this study provides important insights, it has certain limitations. First, although the ARIMA model aids time-series analysis, it may not capture all complex factors influencing disease patterns. Future research could employ more advanced methods, such as machine learning or hybrid approaches, to better analyze multivariable interactions. Second, due to data quality differences, GBD estimates may deviate from actual data, potentially underestimating the disease burden in regions with significant data gaps. The ARIMA and ES models also have limitations in predicting gastrointestinal ulcer burden, as they primarily rely on historical data trends and may struggle to address emerging public health events, policy changes, and socioeconomic fluctuations. Sensitivity analysis and cohort studies should be conducted for regions with large data gaps. Socioeconomic disparities further increase uncertainty in predictions, reflecting differences in healthcare access, health literacy, and treatment adherence, which may introduce systemic biases in model predictions, particularly in resource-scarce areas.

## Data Availability

Publicly available datasets were analyzed in this study. This data can be found here: https://ghdx.healthdata.org/gbd-2021 - GBD 2021.
