# Supplementary material for: Global trends and future predictions of gastrointestinal ulcers in youth
Source: Front Public Health. 2025 Jul 18;13:1511050. doi: 10.3389/fpubh.2025.1511050 (PMC12315113; doi:10.3389/fpubh.2025.1511050)
Supplement: Supplementary file 1 [file Data_Sheet_1.pdf]

Supplementary Material

Supplementary Table1： Global Cases and Age-Standardized Prevalence of Gastrointestinal Ulcers Among Individuals Aged 10–24 Years in 2019

	Number 1990	ASR 1990	Number 2019	ASR 2019	EAPC CI
Global	352786 (228729-496280)	22.78 (14.77-32.04)	407850 (260513-577751)	21.91 (13.99-31.03)	-0.41 (-0.51--0.31)
Sex					
Female	184039 (121495-259832)	24.16 (15.95-34.11)	222518 (145354-316398)	24.5 (16-34.83)	-0.37 (-0.51--0.22)
Male	168748 (109114-239468)	21.44 (13.86-30.42)	185333 (118784-261205)	19.44 (12.46-27.39)	-0.45 (-0.52--0.38)
SDI regions					
High SDI	23041 (14049-33462)	12.57 (7.66-18.25)	20715 (13382-29143)	11.8 (7.62-16.6)	-0.28 (-0.45--0.1)
High-middle SDI	49900 (31163-71731)	16.49 (10.3-23.7)	37019 (23162-53437)	14.59 (9.13-21.06)	-0.43 (-0.48--0.37)
Middle SDI	90348 (56825-130499)	16.6 (10.44-23.97)	85970 (54333-124208)	15.64 (9.89-22.6)	-0.34 (-0.43--0.24)
Low-middle SDI	121570 (80971-171585)	34.35 (22.88-48.49)	137598 (87863-196702)	26.96 (17.21-38.54)	-1.12 (-1.22--1.02)
Low SDI	67778 (45954-94049)	41.37 (28.05-57.4)	126343 (84424-177468)	34.01 (22.73-47.77)	-1.25 (-1.42--1.07)
GBD region					
Africa	66010 (44374-92054)	32.89 (22.11-45.87)	138553 (92103-195829)	32.98 (21.92-46.61)	-0.46 (-0.61--0.3)

African Region	57823 (39016-80401)	35.6 (24.02-49.51)	125021 (83374-176193)	34.96 (23.32-49.27)	-0.57 (-0.75--0.4)
America	20287 (12576-29102)	10.26 (6.36-14.72)	16600 (10720-23114)	7.18 (4.63-9.99)	-1.5 (-1.6--1.39)
Andean Latin America	1220 (781-1769)	9.92 (6.35-14.38)	1120 (690-1612)	6.65 (4.1-9.58)	-1.86 (-2.03--1.69)
Asia	238739 (155000-336761)	24.65 (16-34.77)	231372 (145877-329104)	21.7 (13.68-30.86)	-0.71 (-0.83--0.59)
Australasia	209 (120-317)	4.34 (2.5-6.58)	234 (140-352)	4.32 (2.58-6.48)	0.04 (-0.07-0.15)
Caribbean	1040 (662-1481)	9.76 (6.22-13.9)	1035 (635-1498)	9.04 (5.54-13.08)	-0.56 (-0.66--0.46)
Central Asia	3559 (2353-5022)	17.94 (11.86-25.32)	3762 (2385-5398)	16.64 (10.55-23.87)	-0.38 (-0.46--0.29)
Central Europe	3778 (2337-5451)	13.16 (8.14-18.99)	2505 (1634-3515)	13.93 (9.09-19.55)	0.33 (0.24-0.42)
Central Latin America	2540 (1609-3659)	4.69 (2.97-6.76)	2127 (1312-3136)	3.23 (1.99-4.77)	-1.65 (-1.92--1.38)
Central Sub-Saharan Africa	4589 (3061-6501)	26.5 (17.68-37.55)	11366 (7290-16455)	26.45 (16.96-38.29)	-0.06 (-0.13-0.01)
Commonwealth High Income	1688 (1006-2550)	6.97 (4.16-10.54)	1714 (1034-2497)	6.82 (4.11-9.93)	-0.13 (-0.22--0.04)
Commonwealth Low Income	22274 (15004-31192)	32.88 (22.15-46.04)	34997 (22530-49553)	29.49 (18.99-41.76)	-0.61 (-0.75--0.46)
Commonwealth Middle Income	148290 (98465-210202)	41.17 (27.34-58.36)	192190 (124292-273539)	31.82 (20.58-45.29)	-1.13 (-1.21--1.05)
East Asia	48904 (29831-71325)	13.1 (7.99-19.11)	20186 (12684-28802)	8.52 (5.35-12.16)	-1.61 (-1.74--1.49)
East Asia & Pacific - WB	83447 (52434-120018)	14.76 (9.28-21.23)	53218 (33615-76255)	12.16 (7.68-17.42)	-0.86 (-1.03--0.7)

Eastern Europe	13686 (8664-19621)	28.95 (18.33-41.51)	9084 (5662-13210)	28.1 (17.51-40.86)	0.21 (0.06-0.35)
Eastern Mediterranean Region	34398 (22699-48748)	28.59 (18.87-40.52)	53387 (34310-75804)	25.27 (16.24-35.88)	-0.55 (-0.6--0.5)
Eastern Sub-Saharan Africa	19596 (13206-27205)	31.7 (21.36-44.01)	39613 (26041-55931)	28.36 (18.64-40.04)	-0.66 (-0.76--0.56)
Europe	27521 (17306-39841)	15.29 (9.61-22.13)	21050 (13113-30437)	14.82 (9.23-21.42)	0.09 (-0.12-0.3)
Europe & Central Asia - WB	30073 (19061-43404)	15.48 (9.81-22.34)	24053 (15021-34680)	15.02 (9.38-21.66)	0.06 (-0.14-0.26)
European Region	30149 (19108-43504)	15.37 (9.74-22.17)	24136 (15070-34781)	14.83 (9.26-21.37)	0.04 (-0.16-0.24)
High-income Asia Pacific	9730 (6053-14392)	23.08 (14.36-34.14)	6396 (4057-9233)	23.75 (15.06-34.28)	-0.02 (-0.1-0.07)
High-income North America	7662 (4630-11225)	12.57 (7.6-18.42)	8508 (5721-11662)	11.99 (8.06-16.43)	-0.19 (-0.65-0.26)
Latin America & Caribbean - WB	12690 (7994-18098)	9.21 (5.8-13.14)	8144 (4987-11820)	5.06 (3.1-7.34)	-2.44 (-2.64--2.25)
Middle East & North Africa - WB	16774 (10681-23805)	20.28 (12.91-28.77)	22454 (14025-32520)	19 (11.87-27.51)	-0.27 (-0.29--0.24)
North Africa and Middle East	25520 (16439-36204)	23.11 (14.88-32.78)	36607 (23119-52259)	22.7 (14.33-32.4)	0.01 (-0.08-0.11)
North America	7661 (4629-11223)	12.57 (7.59-18.41)	8507 (5720-11661)	11.99 (8.06-16.43)	-0.19 (-0.65-0.26)
Oceania	849 (586-1184)	40.95 (28.3-57.12)	1356 (902-1933)	34.78 (23.13-49.59)	-0.88 (-1--0.76)
Region of the Americas	20287 (12576-29102)	10.26 (6.36-14.72)	16600 (10720-23114)	7.18 (4.63-9.99)	-1.5 (-1.6--1.39)
South-East Asia Region	139679 (92341-196890)	34.88 (23.06-49.17)	148155 (93503-212646)	26.49 (16.72-38.02)	-1.23 (-1.32--1.13)
South Asia	141297 (93747-200166)	42.19 (27.99-59.76)	156491 (99491-223448)	29.76 (18.92-42.5)	-1.49 (-1.6--1.39)

South Asia - WB	143690 (95427-203651)	41.77 (27.74-59.2)	162536 (103505-232226)	29.87 (19.02-42.68)	-1.44 (-1.53--1.34)
Southeast Asia	24304 (15917-34195)	16.4 (10.74-23.08)	25670 (16174-36608)	15.11 (9.52-21.55)	-0.46 (-0.62--0.3)
Southern Latin America	729 (421-1124)	5.51 (3.18-8.49)	803 (468-1218)	5.28 (3.08-8.01)	-0.2 (-0.24--0.16)
Southern Sub-Saharan Africa	3394 (2151-4823)	19.6 (12.43-27.86)	4250 (2690-6092)	19.93 (12.62-28.57)	0.1 (0.05-0.16)
Sub-Saharan Africa - WB	58243 (39382-81157)	35.82 (24.22-49.91)	128682 (85765-181224)	34.96 (23.3-49.23)	-0.6 (-0.77--0.43)
Tropical Latin America	7197 (4571-10238)	15.05 (9.56-21.41)	3093 (1875-4505)	5.93 (3.59-8.63)	-3.63 (-3.97--3.29)
Western Europe	3733 (2166-5630)	4.54 (2.64-6.85)	3215 (1901-4817)	4.49 (2.66-6.73)	0.03 (-0.02-0.07)
Western Pacific Region	69950 (43607-101194)	14.95 (9.32-21.63)	39898 (25232-56762)	11.91 (7.53-16.94)	-0.96 (-1.14--0.78)
Western Sub-Saharan Africa	29250 (20191-40441)	49.09 (33.89-67.87)	70429 (47785-98668)	46.7 (31.68-65.42)	-0.97 (-1.27--0.66)
World Bank High Income	26008 (15806-37807)	11.54 (7.01-16.78)	23021 (14879-32316)	11.11 (7.18-15.6)	-0.18 (-0.32--0.04)
World Bank Low Income	36053 (24743-50124)	34.85 (23.92-48.46)	73385 (49124-103340)	31.6 (21.15-44.49)	-1.15 (-1.42--0.88)
World Bank Lower Middle Income	200851 (133258-281493)	34.07 (22.61-47.75)	256454 (164679-367228)	27.96 (17.96-40.04)	-0.9 (-0.98--0.82)
World Bank Upper Middle Income	89722 (55850-129788)	14.24 (8.87-20.61)	54784 (34183-78644)	10.87 (6.78-15.6)	-1 (-1.06--0.94)
Countries					
Afghanistan	1824 (1305-2586)	48.1 (34.39-68.18)	5417 (3631-7736)	41.68 (27.94-59.52)	-0.68 (-1.07--0.28)
Albania	121 (74-179)	12.21 (7.47-18.01)	81 (49-119)	14.31 (8.68-21.05)	0.58 (0.45-0.71)

Algeria	1665 (1072-2397)	19.51 (12.56-28.09)	1788 (1095-2607)	18.3 (11.21-26.67)	-0.3 (-0.35--0.25)
American Samoa	4 (3-6)	28.13 (18.45-40.69)	4 (3-7)	26 (16.55-38.88)	-0.62 (-0.78--0.45)
Andorra	0 (0-1)	3.8 (2.11-5.86)	0 (0-1)	3.92 (2.22-6.05)	-0.1 (-0.16--0.04)
Angola	901 (602-1288)	28.08 (18.75-40.14)	2529 (1643-3644)	26.27 (17.07-37.85)	-0.29 (-0.43--0.15)
Antigua and Barbuda	1 (1-2)	6.94 (4.05-10.41)	2 (1-2)	7.66 (4.44-11.33)	0.24 (0.15-0.33)
Argentina	494 (282-759)	5.67 (3.23-8.71)	565 (330-865)	5.31 (3.1-8.12)	-0.19 (-0.27--0.1)
Armenia	153 (101-219)	17.56 (11.64-25.13)	84 (52-123)	15.5 (9.49-22.53)	-0.39 (-0.57--0.21)
Australia	169 (98-257)	4.26 (2.47-6.48)	192 (112-292)	4.23 (2.46-6.44)	0.01 (-0.07-0.09)
Austria	179 (114-253)	10.99 (6.99-15.58)	108 (65-158)	7.63 (4.62-11.19)	-1.37 (-1.52--1.23)
Azerbaijan	439 (296-630)	20.73 (14-29.75)	361 (224-523)	16.4 (10.18-23.72)	-1.06 (-1.22--0.9)
Bahamas	5 (3-8)	6.79 (4.09-9.97)	7 (4-11)	7.54 (4.49-11.3)	0.22 (0.15-0.29)
Bahrain	22 (14-32)	17.13 (10.87-24.61)	45 (28-67)	18.21 (11.25-26.82)	0.3 (0.19-0.41)
Bangladesh	12885 (8608-18180)	36.61 (24.46-51.66)	13636 (8360-20049)	30.09 (18.45-44.24)	-0.99 (-1.28--0.7)
Barbados	4 (3-7)	6.64 (3.95-9.88)	4 (2-6)	6.84 (3.94-10.19)	-0.14 (-0.2--0.07)
Belarus	612 (376-904)	27.59 (16.98-40.77)	390 (242-579)	27.67 (17.2-41.1)	0.32 (0.22-0.42)
Belgium	106 (61-161)	5.24 (3.03-8.02)	104 (60-158)	5.36 (3.12-8.14)	0.63 (0.34-0.92)
Belize	5 (3-7)	7.74 (4.76-11.28)	11 (7-16)	8.69 (5.14-12.69)	0.35 (0.32-0.38)

Benin	621 (431-866)	43.08 (29.85-60.05)	1594 (1040-2288)	38.84 (25.35-55.74)	-0.58 (-0.65--0.52)
Bermuda	1 (0-1)	6.77 (3.97-10.19)	1 (0-1)	7.05 (4.11-10.76)	0.12 (0.06-0.18)
Bhutan	119 (84-167)	55.61 (39.36-77.96)	49 (30-71)	22.77 (14.11-33.1)	-3.93 (-4.36--3.5)
Bolivia (Plurinational State of)	248 (163-355)	12.39 (8.12-17.75)	244 (147-359)	7.53 (4.54-11.06)	-2.29 (-2.48--2.09)
Bosnia and Herzegovina	178 (114-257)	15.2 (9.77-21.96)	79 (48-117)	13.94 (8.52-20.53)	-0.31 (-0.41--0.21)
Botswana	101 (63-143)	22.46 (14.06-31.69)	127 (78-183)	19.52 (11.95-28.22)	0 (-0.32-0.33)
Brazil	7067 (4490-10064)	15.17 (9.64-21.61)	2946 (1791-4296)	5.86 (3.57-8.55)	-3.71 (-4.06--3.35)
Brunei Darussalam	16 (10-24)	21.13 (13.48-31.26)	23 (14-34)	21.47 (13.04-31.61)	0.12 (0.01-0.22)
Bulgaria	210 (129-305)	11.39 (6.99-16.59)	119 (73-174)	12.33 (7.54-18.08)	0.46 (0.36-0.57)
Burkina Faso	1496 (1055-2069)	50.66 (35.72-70.03)	3710 (2539-5131)	51.36 (35.14-71.03)	-0.04 (-0.12-0.04)
Burundi	643 (449-888)	38.36 (26.81-53.02)	1266 (865-1833)	33.03 (22.57-47.84)	-0.46 (-0.53--0.39)
Cabo Verde	49 (32-69)	42.71 (28.39-60.5)	50 (32-74)	32.69 (20.68-47.83)	-1.09 (-1.37--0.8)
Cambodia	1153 (820-1585)	36.03 (25.63-49.54)	821 (523-1163)	17.94 (11.43-25.43)	-2.89 (-3.07--2.71)
Cameroon	1470 (1024-2037)	45.64 (31.77-63.23)	3749 (2489-5390)	38.68 (25.69-55.62)	-0.74 (-0.85--0.64)
Canada	690 (416-1023)	11.84 (7.14-17.55)	762 (452-1122)	12.06 (7.16-17.75)	0.18 (0.06-0.29)

Central African Republic	294 (204-407)	34.61 (24-47.89)	590 (389-821)	33.71 (22.22-46.93)	-0.14 (-0.19--0.1)
Chad	1008 (722-1373)	55.28 (39.6-75.25)	2820 (1978-3810)	52.55 (36.85-71)	-0.3 (-0.51--0.09)
Chile	196 (113-299)	5.21 (3-7.95)	199 (115-299)	5.21 (3.01-7.83)	-0.26 (-0.56-0.05)
China	47718 (29028-69672)	13.18 (8.02-19.24)	19426 (12212-27725)	8.53 (5.36-12.18)	-1.63 (-1.76--1.5)
Colombia	436 (270-631)	4.26 (2.64-6.17)	532 (322-808)	4.5 (2.72-6.83)	0.63 (-0.25-1.53)
Comoros	51 (36-71)	32.97 (23.41-46.27)	62 (41-89)	29.07 (19.11-41.27)	-0.66 (-0.71--0.6)
Congo	203 (135-285)	25.04 (16.6-35.09)	358 (227-521)	22.93 (14.53-33.34)	-0.41 (-0.46--0.37)
Cook Islands	1 (1-2)	23.05 (14.8-33.94)	1 (1-2)	24.69 (15.43-36.26)	0.16 (0.09-0.22)
Costa Rica	9 (4-15)	0.96 (0.48-1.67)	14 (7-24)	1.24 (0.65-2.13)	1 (0.72-1.29)
Croatia	133 (81-194)	12.91 (7.85-18.79)	70 (44-100)	10.32 (6.52-14.73)	-1.63 (-1.92--1.34)
Cuba	237 (136-352)	7.66 (4.39-11.38)	155 (89-229)	7.77 (4.5-11.52)	-0.04 (-0.17-0.09)
Cyprus	4 (2-6)	2.1 (1.16-3.2)	5 (3-8)	2.41 (1.31-3.73)	0.82 (0.65-1)
Czechia	183 (110-274)	7.72 (4.62-11.51)	143 (87-209)	9.43 (5.71-13.76)	0.51 (0.37-0.64)
C<U+00F4>te d'Ivoire	1810 (1269-2549)	47.3 (33.18-66.61)	3528 (2367-4971)	43.7 (29.33-61.58)	-0.47 (-0.6--0.33)
Democratic People's Republic of Korea	728 (480-1030)	13.18 (8.69-18.63)	496 (308-722)	9.02 (5.6-13.13)	-1.41 (-1.54--1.28)
Democratic Republic of the Congo	3084 (2023-4420)	25.67 (16.84-36.8)	7658 (4931-11077)	26.43 (17.02-38.23)	0.06 (0-0.12)

Denmark	45 (26-69)	4.11 (2.35-6.3)	49 (28-74)	4.55 (2.63-6.94)	0.28 (0.15-0.42)
Djibouti	49 (33-69)	28.98 (19.79-40.91)	86 (55-122)	26.09 (16.86-37.05)	-0.34 (-0.4--0.28)
Dominica	2 (1-2)	7.38 (4.4-10.79)	1 (1-2)	7.73 (4.53-11.4)	0.08 (0.02-0.15)
Dominican Republic	211 (133-311)	8.87 (5.57-13.08)	254 (153-374)	8.75 (5.27-12.89)	-0.27 (-0.38--0.15)
Ecuador	300 (195-429)	9.21 (5.99-13.14)	258 (175-357)	5.26 (3.56-7.28)	-2.54 (-2.76--2.31)
Egypt	3215 (2057-4596)	18.74 (11.99-26.78)	5228 (3287-7782)	18.41 (11.57-27.4)	-0.1 (-0.16--0.05)
El Salvador	85 (54-119)	4.77 (3.04-6.72)	50 (28-76)	2.85 (1.6-4.32)	-2.51 (-2.85--2.17)
Equatorial Guinea	39 (26-56)	30.58 (20.4-43.52)	119 (74-174)	22.43 (14.03-32.85)	-1.52 (-1.72--1.32)
Eritrea	316 (216-440)	31.99 (21.9-44.52)	705 (482-978)	31.23 (21.37-43.32)	-0.27 (-0.36--0.18)
Estonia	69 (42-102)	21.2 (13-31.13)	48 (30-70)	24.2 (14.94-35.55)	1.61 (1.07-2.14)
Eswatini	59 (37-86)	21.41 (13.51-31.4)	78 (49-115)	21.37 (13.35-31.37)	0.1 (-0.03-0.23)
Ethiopia	6412 (4245-9017)	39.09 (25.88-54.96)	9900 (6444-14264)	26.55 (17.28-38.25)	-1.76 (-1.92--1.6)
Fiji	74 (50-105)	31.13 (21.01-44.28)	65 (41-96)	27.58 (17.26-40.48)	-0.85 (-0.99--0.72)
Finland	40 (23-61)	4.16 (2.39-6.25)	40 (23-62)	4.34 (2.52-6.71)	0.05 (-0.32-0.42)
France	470 (265-714)	3.72 (2.09-5.65)	459 (264-720)	3.8 (2.18-5.96)	0.09 (0.05-0.13)
Gabon	68 (44-97)	22.22 (14.39-31.83)	112 (69-164)	21.02 (13.06-30.94)	-0.29 (-0.38--0.19)

Gambia	140 (97-195)	44.56 (30.79-62.31)	292 (194-417)	38.04 (25.29-54.33)	-0.66 (-0.76--0.56)
Georgia	217 (137-315)	16.93 (10.67-24.52)	125 (83-179)	20 (13.21-28.49)	0.79 (0.6-0.98)
Germany	708 (401-1092)	4.75 (2.69-7.34)	659 (380-1006)	5.15 (2.97-7.88)	1.29 (0.97-1.61)
Ghana	1587 (1052-2291)	33.65 (22.31-48.6)	3881 (2543-5538)	39.08 (25.6-55.76)	0.62 (0.54-0.69)
Greece	85 (48-129)	3.61 (2.04-5.5)	60 (35-93)	3.88 (2.24-6)	0.27 (0.15-0.38)
Greenland	2 (1-3)	15.95 (9.94-23.22)	1 (1-2)	12.23 (7.48-17.68)	-0.72 (-0.98--0.46)
Grenada	2 (1-3)	7.26 (4.3-10.66)	2 (1-3)	7.87 (4.64-11.85)	0.21 (0.14-0.27)
Guam	9 (6-14)	23.75 (14.77-34.8)	11 (7-16)	26.12 (16.53-37.99)	0.29 (0.15-0.43)
Guatemala	332 (232-457)	13.35 (9.32-18.33)	326 (198-478)	5.78 (3.51-8.46)	-3.68 (-4.49--2.87)
Guinea	920 (641-1253)	52.09 (36.29-70.96)	1766 (1195-2470)	43.48 (29.43-60.82)	-0.9 (-1--0.79)
Guinea-Bissau	193 (136-259)	59.6 (42.01-80.23)	313 (212-435)	49.94 (33.79-69.32)	-0.75 (-0.87--0.63)
Guyana	32 (21-44)	12.18 (8.25-16.91)	23 (14-33)	10.19 (6.33-14.62)	-1.25 (-1.53--0.97)
Haiti	341 (236-474)	17.56 (12.16-24.42)	396 (252-573)	10.77 (6.84-15.59)	-2.07 (-2.22--1.92)
Honduras	142 (93-199)	9.24 (6.08-12.99)	122 (73-182)	3.9 (2.31-5.8)	-3.76 (-4.07--3.45)
Hungary	254 (155-372)	11.03 (6.74-16.14)	187 (113-275)	12.51 (7.57-18.42)	0.39 (0.32-0.46)
Iceland	2 (1-3)	2.74 (1.54-4.21)	2 (1-3)	3.18 (1.8-4.84)	0.6 (0.55-0.64)

India	110074 (72728-156107)	42.68 (28.2-60.53)	118527 (75082-169008)	29.83 (18.9-42.54)	-1.51 (-1.6--1.43)
Indonesia	6478 (4051-9412)	10.89 (6.81-15.82)	8488 (5252-12355)	12.5 (7.74-18.2)	0.36 (0.28-0.44)
Iran (Islamic Republic of)	4776 (3057-6810)	24.87 (15.92-35.47)	3688 (2302-5305)	20.95 (13.08-30.14)	-0.35 (-0.46--0.23)
Iraq	962 (605-1393)	16.9 (10.63-24.48)	2462 (1522-3662)	18.64 (11.53-27.74)	0.28 (0.15-0.41)
Ireland	34 (20-53)	3.53 (2.03-5.46)	36 (21-54)	3.73 (2.2-5.7)	0.15 (0-0.3)
Israel	19 (10-32)	1.38 (0.7-2.36)	32 (16-55)	1.5 (0.75-2.55)	-2.74 (-3.99--1.48)
Italy	678 (395-1036)	5.42 (3.16-8.28)	432 (270-619)	4.96 (3.1-7.1)	-0.76 (-0.9--0.61)
Jamaica	58 (36-85)	7.63 (4.74-11.16)	58 (34-87)	7.87 (4.63-11.78)	-0.12 (-0.19--0.05)
Japan	6779 (4222-9990)	24.12 (15.02-35.55)	4348 (2783-6199)	24.56 (15.72-35.02)	-0.13 (-0.23--0.03)
Jordan	184 (112-269)	13.42 (8.19-19.7)	566 (359-823)	15.97 (10.14-23.24)	0.7 (0.6-0.8)
Kazakhstan	769 (503-1089)	17.6 (11.53-24.93)	603 (379-865)	15.43 (9.7-22.15)	-0.48 (-0.6--0.37)
Kenya	2184 (1417-3121)	27.56 (17.88-39.39)	4805 (3129-6899)	28.02 (18.25-40.24)	-0.16 (-0.23--0.09)
Kiribati	22 (17-29)	100.45 (74.12-131.99)	24 (17-33)	69.17 (50.14-93.99)	-1.68 (-1.94--1.42)
Kuwait	76 (46-110)	15.99 (9.8-23.2)	147 (89-215)	18.35 (11.1-26.78)	0.54 (0.49-0.59)
Kyrgyzstan	195 (128-285)	15.1 (9.91-22.07)	245 (152-364)	14.46 (8.95-21.43)	0.07 (-0.08-0.22)
Lao People's Democratic Republic	597 (439-814)	46.13 (33.91-62.83)	477 (322-672)	22.67 (15.3-31.9)	-2.95 (-3.16--2.74)

Latvia	147 (92-217)	27.16 (16.98-40.28)	74 (46-110)	27.09 (16.68-40)	0.93 (0.62-1.23)
Lebanon	171 (108-247)	18.18 (11.51-26.3)	216 (132-314)	19.55 (11.97-28.45)	0.49 (0.37-0.6)
Lesotho	135 (85-198)	22.61 (14.23-33.07)	166 (107-241)	25.49 (16.39-36.9)	0.63 (0.5-0.75)
Liberia	302 (211-418)	52.27 (36.49-72.21)	662 (440-952)	41.71 (27.7-59.98)	-1.29 (-1.49--1.1)
Libya	235 (148-350)	16.45 (10.34-24.47)	327 (201-469)	18.88 (11.64-27.1)	0.41 (0.36-0.46)
Lithuania	288 (175-428)	35.25 (21.42-52.36)	156 (96-232)	35.97 (22.19-53.63)	0.37 (0.27-0.47)
Luxembourg	4 (2-6)	5.35 (3.1-8.3)	6 (3-9)	5.45 (3.18-8.33)	-0.47 (-0.61--0.33)
Madagascar	1172 (827-1669)	30.62 (21.59-43.58)	2628 (1751-3645)	29.84 (19.88-41.39)	-0.36 (-0.51--0.21)
Malawi	897 (623-1245)	29.4 (20.42-40.8)	1997 (1344-2814)	30.06 (20.23-42.34)	-0.18 (-0.33--0.02)
Malaysia	476 (289-701)	8.85 (5.37-13.03)	912 (545-1330)	11.17 (6.68-16.29)	0.77 (0.71-0.82)
Maldives	8 (5-12)	11.41 (7.01-16.7)	15 (9-23)	15.07 (9.12-22.21)	1.32 (1.17-1.48)
Mali	1378 (970-1859)	54.18 (38.12-73.06)	3389 (2330-4730)	46.44 (31.93-64.82)	-4.54 (-6.03--3.03)
Malta	2 (1-3)	2.04 (1.13-3.16)	2 (1-2)	2.4 (1.27-3.7)	0.99 (0.84-1.15)
Marshall Islands	7 (5-10)	48.74 (34.02-66.98)	6 (4-8)	34.84 (23.15-50.26)	-1.51 (-1.7--1.32)
Mauritania	276 (191-382)	43.41 (29.95-60.03)	481 (308-696)	35.68 (22.87-51.69)	-0.9 (-0.97--0.84)
Mauritius	32 (19-47)	9.8 (5.89-14.47)	29 (17-42)	10.74 (6.51-15.63)	0.07 (-0.07-0.21)

Mexico	1340 (806-1988)	4.6 (2.77-6.83)	842 (526-1218)	2.58 (1.61-3.73)	-2.83 (-3.15--2.51)
Micronesia (Federated States of)	19 (13-26)	54.66 (38.64-76.87)	11 (7-16)	34.18 (22.7-48.72)	-2.14 (-2.31--1.98)
Monaco	0 (0-0)	3.88 (2.2-5.9)	0 (0-0)	3.91 (2.23-6.04)	0.09 (-0.01-0.2)
Mongolia	139 (94-195)	19.62 (13.29-27.62)	136 (88-195)	18.6 (12.03-26.7)	-0.12 (-0.23-0)
Montenegro	19 (11-27)	11.78 (7.13-17.3)	16 (10-23)	13.26 (8.06-19.35)	0.44 (0.39-0.49)
Morocco	2123 (1385-3072)	26.19 (17.09-37.89)	1975 (1243-2878)	21.23 (13.37-30.94)	-0.99 (-1.1--0.87)
Mozambique	1187 (805-1657)	29.09 (19.74-40.62)	3233 (2224-4513)	32.73 (22.52-45.69)	0.22 (0.11-0.33)
Myanmar	3992 (2822-5477)	31.13 (22.01-42.71)	2324 (1467-3367)	15.9 (10.04-23.04)	-2.93 (-3.11--2.75)
Namibia	101 (64-148)	21.36 (13.47-31.25)	147 (93-215)	20.19 (12.73-29.46)	-0.2 (-0.25--0.15)
Nauru	1 (1-1)	31.76 (21.23-44.35)	1 (1-1)	30.64 (20.12-44.04)	-0.26 (-0.56-0.04)
Nepal	2686 (1842-3721)	45.13 (30.96-62.53)	2089 (1331-2952)	21.87 (13.94-30.9)	-3.58 (-4.05--3.1)
Netherlands	126 (70-195)	3.85 (2.15-5.95)	119 (67-184)	3.92 (2.22-6.07)	0.05 (-0.03-0.13)
New Zealand	40 (23-61)	4.7 (2.69-7.27)	42 (28-60)	4.76 (3.11-6.76)	0.2 (-0.09-0.48)
Nicaragua	39 (24-59)	3.01 (1.82-4.53)	51 (29-78)	2.69 (1.54-4.11)	-0.79 (-0.91--0.67)
Niger	1538 (1117-2060)	62.88 (45.67-84.26)	3729 (2575-5167)	48.37 (33.4-67.02)	-5.16 (-6.69--3.61)
Nigeria	14248 (9577-20164)	50.61 (34.02-71.62)	36245 (24454-51537)	49.93 (33.69-71)	-0.16 (-0.28--0.03)

Niue	0 (0-0)	25.43 (16.43-37.15)	0 (0-0)	26.12 (16.19-38.29)	-0.04 (-0.12-0.04)
North Macedonia	58 (35-85)	11.14 (6.78-16.31)	50 (31-74)	13.21 (8.06-19.5)	0.72 (0.67-0.77)
Northern Mariana Islands	3 (2-4)	22.03 (13.58-32.75)	3 (2-4)	26.89 (16.59-39.97)	0.58 (0.41-0.75)
Norway	106 (64-156)	11.56 (6.94-17.02)	132 (79-195)	13.4 (8.03-19.83)	-0.38 (-0.71--0.04)
Oman	100 (62-145)	18.17 (11.32-26.38)	193 (118-288)	20.35 (12.46-30.32)	0.48 (0.41-0.54)
Pakistan	15533 (10399-22092)	43.51 (29.13-61.88)	22189 (14463-32030)	30.23 (19.71-43.64)	-1.54 (-1.64--1.44)
Palau	1 (1-2)	24.41 (15.46-35.48)	1 (1-1)	26.67 (16.73-39.05)	0.25 (0.17-0.33)
Palestine	132 (84-195)	19.56 (12.46-28.81)	300 (186-438)	18.65 (11.53-27.18)	-0.69 (-1.18--0.2)
Panama	17 (10-26)	2.19 (1.28-3.38)	26 (15-39)	2.4 (1.39-3.67)	0.16 (0.11-0.21)
Papua New Guinea	536 (363-745)	40.93 (27.72-56.91)	1008 (667-1448)	34.54 (22.85-49.6)	-0.89 (-1--0.77)
Paraguay	131 (80-192)	10.6 (6.5-15.61)	148 (87-219)	7.57 (4.45-11.22)	-1.13 (-1.26--1)
Peru	672 (421-986)	9.55 (5.99-14.01)	617 (364-911)	7.11 (4.19-10.5)	-1.41 (-1.6--1.22)
Philippines	6699 (4413-9426)	32.65 (21.51-45.95)	7439 (4746-10601)	23.21 (14.8-33.07)	-1.3 (-1.75--0.85)
Poland	1531 (950-2219)	17.82 (11.06-25.82)	1040 (722-1393)	17.71 (12.3-23.73)	0.37 (0.18-0.56)
Portugal	63 (34-97)	2.51 (1.36-3.88)	50 (28-76)	3 (1.68-4.56)	0.63 (0.51-0.76)
Puerto Rico	61 (36-92)	6.26 (3.67-9.39)	48 (28-72)	7.2 (4.22-10.7)	0.42 (0.38-0.47)

Qatar	21 (13-30)	20.75 (12.83-30.53)	129 (78-189)	25.36 (15.21-37.11)	0.97 (0.81-1.14)
Republic of Korea	2757 (1709-4051)	20.95 (12.99-30.8)	1865 (1148-2804)	22.26 (13.71-33.48)	0.28 (0.2-0.35)
Republic of Moldova	309 (196-444)	29.98 (19.09-43.19)	185 (115-276)	30.4 (18.81-45.29)	0.28 (0.19-0.36)
Romania	680 (419-995)	11.51 (7.1-16.85)	376 (233-556)	12.08 (7.48-17.86)	0.58 (0.4-0.77)
Russian Federation	8751 (5546-12675)	27.78 (17.6-40.23)	6098 (3792-8847)	26.74 (16.63-38.8)	0.19 (0.01-0.37)
Rwanda	1027 (733-1404)	45.44 (32.43-62.14)	1375 (945-1928)	32.78 (22.54-45.97)	-1.37 (-1.51--1.23)
Saint Kitts and Nevis	1 (0-1)	6.52 (3.89-9.8)	1 (1-1)	7.31 (4.26-10.87)	0.4 (0.36-0.44)
Saint Lucia	3 (2-5)	7 (4.21-10.19)	3 (2-5)	7.76 (4.56-11.51)	0.3 (0.23-0.37)
Saint Vincent and the Grenadines	3 (2-4)	8.09 (4.91-11.74)	2 (1-3)	8.13 (4.84-12.05)	-0.21 (-0.28--0.14)
Samoa	18 (12-25)	29.98 (19.72-42.97)	19 (12-27)	27.26 (17.66-39.61)	-0.67 (-0.77--0.57)
San Marino	0 (0-0)	3.66 (2.07-5.63)	0 (0-0)	3.89 (2.23-5.93)	0.15 (0.12-0.19)
Sao Tome and Principe	16 (11-23)	40.12 (27.06-57.27)	22 (14-31)	31.86 (20.53-46.18)	-1.21 (-1.41--1.02)
Saudi Arabia	883 (552-1274)	17.35 (10.86-25.04)	1554 (944-2255)	18.77 (11.41-27.24)	0.37 (0.28-0.45)
Senegal	1129 (784-1533)	46.67 (32.42-63.37)	1910 (1268-2684)	38.18 (25.35-53.67)	-0.93 (-1--0.86)
Serbia	189 (113-277)	9.02 (5.37-13.23)	178 (108-262)	11.34 (6.87-16.69)	0.4 (0.31-0.5)
Seychelles	6 (4-8)	25.01 (18.14-34.05)	4 (3-6)	18.48 (12.26-26.64)	-1.31 (-1.42--1.19)

Sierra Leone	535 (374-731)	49.95 (34.95-68.3)	1303 (905-1816)	47.39 (32.89-66.05)	-0.27 (-0.37--0.17)
Singapore	179 (109-264)	21.72 (13.18-32.06)	161 (99-239)	21.4 (13.23-31.9)	0.05 (0-0.11)
Slovakia	176 (108-258)	13.83 (8.52-20.27)	132 (80-196)	15.65 (9.46-23.29)	-0.31 (-0.51--0.11)
Slovenia	46 (28-69)	10.46 (6.3-15.48)	34 (20-50)	11.57 (6.9-17.27)	-0.59 (-0.89--0.28)
Solomon Islands	60 (42-84)	52.24 (36.32-72.4)	83 (57-117)	40.94 (27.99-57.71)	-1.11 (-1.25--0.97)
Somalia	644 (439-925)	28.11 (19.18-40.37)	2340 (1581-3240)	34.16 (23.09-47.31)	0.31 (0.04-0.58)
South Africa	2117 (1328-3058)	17.75 (11.14-25.64)	2475 (1542-3588)	17.64 (11-25.58)	0.08 (-0.02-0.18)
South Sudan	514 (344-723)	25.72 (17.19-36.16)	856 (550-1222)	26.47 (17.01-37.79)	-0.03 (-0.1-0.05)
Spain	361 (207-554)	3.71 (2.13-5.69)	262 (149-402)	3.79 (2.16-5.82)	-0.02 (-0.13-0.1)
Sri Lanka	561 (344-826)	10.83 (6.64-15.94)	613 (376-899)	11.88 (7.29-17.45)	0.3 (0.25-0.34)
Sudan	1440 (920-2085)	22.32 (14.26-32.32)	3110 (1944-4502)	23.12 (14.45-33.47)	-0.01 (-0.08-0.06)
Suriname	12 (8-17)	9.88 (6.24-14.19)	11 (7-17)	7.94 (4.73-11.66)	-1.01 (-1.09--0.93)
Sweden	98 (57-149)	5.92 (3.42-9)	110 (64-167)	6.37 (3.69-9.68)	0.37 (0.27-0.48)
Switzerland	54 (31-83)	4.08 (2.32-6.26)	56 (32-87)	4.16 (2.39-6.44)	0.18 (0.13-0.23)
Syrian Arab Republic	356 (210-527)	8.16 (4.81-12.09)	560 (340-833)	11.49 (6.97-17.08)	0.73 (0.25-1.2)
Taiwan (Province of China)	458 (276-679)	8.01 (4.83-11.88)	264 (151-396)	6.99 (4.01-10.49)	-0.56 (-0.66--0.46)

Tajikistan	375 (256-522)	22.59 (15.45-31.49)	482 (309-709)	17.85 (11.45-26.27)	-1.2 (-1.35--1.04)
Thailand	2068 (1255-3060)	11.56 (7.02-17.11)	1825 (1097-2672)	13.91 (8.36-20.36)	0.63 (0.55-0.71)
Timor-Leste	79 (57-110)	34.52 (24.92-47.98)	93 (63-131)	20.29 (13.73-28.74)	-2.28 (-2.42--2.14)
Togo	533 (372-749)	44.7 (31.18-62.81)	985 (655-1407)	39.89 (26.51-57)	-0.59 (-0.65--0.52)
Tokelau	0 (0-0)	26.55 (17.26-38.64)	0 (0-0)	26.58 (16.95-39.4)	-0.19 (-0.3--0.08)
Tonga	11 (7-15)	32.15 (21.09-46.11)	9 (6-13)	29.07 (18.78-42.04)	-0.68 (-0.78--0.57)
Trinidad and Tobago	24 (15-35)	6.97 (4.22-10.17)	20 (12-29)	7.35 (4.31-10.75)	0.24 (0.07-0.41)
Tunisia	480 (298-700)	17.95 (11.16-26.19)	467 (287-675)	18.81 (11.55-27.16)	0.32 (0.27-0.37)
Turkmenistan	208 (137-303)	18.1 (11.93-26.36)	249 (162-354)	19.84 (12.91-28.24)	0.33 (0.08-0.58)
Tuvalu	1 (1-1)	32.36 (21.3-46.21)	1 (1-1)	27.75 (17.87-40.62)	-0.84 (-0.96--0.71)
Türkiye	5534 (3487-8132)	29.13 (18.36-42.81)	5708 (3507-8514)	30 (18.43-44.75)	0.66 (0.31-1.01)
Uganda	1597 (1067-2281)	28.25 (18.88-40.35)	4050 (2661-5779)	28.13 (18.48-40.13)	-0.26 (-0.36--0.16)
Ukraine	3511 (2227-4999)	32.4 (20.55-46.13)	2133 (1328-3121)	32.32 (20.12-47.29)	0.2 (0.14-0.27)
United Arab Emirates	70 (43-102)	16.58 (10.13-24.18)	184 (112-266)	17.34 (10.55-25.16)	0.46 (0.16-0.77)
United Kingdom	546 (308-858)	4.62 (2.6-7.25)	490 (299-723)	4.16 (2.54-6.14)	-0.77 (-0.96--0.59)

United Republic of Tanzania	1962 (1282-2826)	23.04 (15.05-33.18)	4597 (2926-6611)	25.06 (15.95-36.04)	0.13 (0.06-0.21)
United States of America	6970 (4213-10194)	12.65 (7.64-18.5)	7744 (5268-10542)	11.98 (8.15-16.31)	-0.23 (-0.72-0.27)
United States Virgin Islands	2 (1-3)	6.36 (3.76-9.58)	1 (1-2)	7.07 (4.12-10.53)	0.28 (0.21-0.35)
Uruguay	39 (23-60)	5.06 (2.96-7.89)	39 (23-60)	5.23 (3.09-8.07)	0.09 (0.05-0.13)
Uzbekistan	1065 (695-1530)	16.66 (10.88-23.94)	1476 (927-2150)	16.49 (10.36-24.02)	-0.25 (-0.44--0.07)
Vanuatu	34 (24-47)	71.35 (50.25-99.97)	45 (31-63)	50.22 (34.71-70.67)	-1.58 (-1.79--1.37)
Venezuela (Bolivarian Republic of)	140 (81-213)	2.34 (1.35-3.55)	164 (94-253)	2.44 (1.4-3.77)	0.06 (-0.03-0.15)
Viet Nam	2122 (1298-3136)	9.85 (6.02-14.55)	2596 (1562-3773)	12.31 (7.4-17.89)	0.96 (0.89-1.02)
Yemen	1236 (816-1779)	29.26 (19.32-42.12)	2504 (1603-3601)	24.64 (15.77-35.43)	-0.86 (-1.01--0.7)
Zambia	926 (652-1282)	33.4 (23.5-46.24)	1681 (1108-2427)	27.3 (18-39.42)	-0.98 (-1.13--0.82)
Zimbabwe	881 (574-1237)	24.54 (16-34.47)	1256 (822-1780)	25.65 (16.78-36.35)	0.08 (0.04-0.12)

Supplementary Table 2： Global Cases and Age-Standardized Incidence of Gastrointestinal Ulcers Among Individuals Aged 10–24 Years in 2019

	**Number 1990**	**ASR 1990**	**Number 2019**	**ASR 2019**	**EAPC CI**
Global	854424 (585815-1200718)	55.16 (37.82-77.52)	958842 (639698-1371106)	51.5 (34.36-73.64)	-0.59 (-0.73--0.46)
Sex					
Female	474920 (326039-662561)	62.34 (42.8-86.97)	547512 (367728-772896)	60.27 (40.48-85.08)	-0.64 (-0.82--0.46)
Male	379504 (253356-544780)	48.21 (32.19-69.21)	411330 (268586-595523)	43.14 (28.17-62.46)	-0.52 (-0.59--0.44)
SDI regions					
High SDI	52905 (32315-78844)	28.85 (17.62-43)	47829 (32084-67913)	27.25 (18.28-38.69)	-0.26 (-0.45--0.08)
High-middle SDI	112968 (71648-165977)	37.33 (23.68-54.85)	83547 (52573-124250)	32.92 (20.72-48.97)	-0.42 (-0.48--0.36)
Middle SDI	208040 (134759-302857)	38.21 (24.75-55.63)	194881 (124651-286017)	35.46 (22.68-52.05)	-0.41 (-0.51--0.3)
Low-middle SDI	306753 (213980-426352)	86.68 (60.47-120.48)	324442 (212831-467452)	63.56 (41.7-91.58)	-1.47 (-1.6--1.35)
Low SDI	173369 (124723-235771)	105.81 (76.12-143.89)	307638 (214461-429010)	82.81 (57.73-115.49)	-1.54 (-1.76--1.31)
GBD region					
Africa	159920 (111318-220926)	79.69 (55.47-110.09)	335927 (229920-472571)	79.96 (54.73-112.49)	-0.53 (-0.72--0.33)
African Region	140966 (99018-194377)	86.8 (60.97-119.69)	305711 (210131-427545)	85.5 (58.77-119.57)	-0.64 (-0.85--0.42)

America	44932 (27882-65911)	22.73 (14.11-33.34)	37762 (25488-53419)	16.32 (11.02-23.09)	-1.36 (-1.48--1.24)
Andean Latin America	2782 (1818-4067)	22.62 (14.78-33.07)	2447 (1542-3609)	14.54 (9.16-21.44)	-2.07 (-2.26--1.88)
Asia	587659 (402962-825564)	60.67 (41.6-85.23)	537470 (349914-775953)	50.4 (32.81-72.76)	-1.01 (-1.15--0.87)
Australasia	432 (254-677)	8.97 (5.29-14.07)	484 (290-739)	8.92 (5.34-13.62)	0.05 (-0.07-0.16)
Caribbean	2354 (1525-3407)	22.1 (14.32-32)	2268 (1381-3384)	19.8 (12.06-29.54)	-0.74 (-0.86--0.61)
Central Asia	7918 (5283-11340)	39.91 (26.63-57.17)	8280 (5179-12223)	36.62 (22.9-54.05)	-0.37 (-0.45--0.29)
Central Europe	8106 (4892-11977)	28.24 (17.04-41.72)	5309 (3491-7607)	29.53 (19.42-42.31)	0.29 (0.18-0.41)
Central Latin America	6027 (3778-9008)	11.14 (6.98-16.65)	5171 (3256-7824)	7.86 (4.95-11.89)	-1.63 (-1.99--1.28)
Central Sub-Saharan Africa	10620 (7337-14782)	61.34 (42.37-85.38)	26038 (17314-37558)	60.58 (40.28-87.39)	-0.1 (-0.18--0.02)
Commonwealth High Income	3791 (2250-5790)	15.67 (9.3-23.93)	3819 (2351-5723)	15.19 (9.35-22.76)	-0.13 (-0.22--0.05)
Commonwealth Low Income	50937 (35494-70823)	75.19 (52.39-104.54)	78712 (51936-113494)	66.33 (43.76-95.64)	-0.68 (-0.83--0.53)
Commonwealth Middle Income	381572 (265049-528117)	105.94 (73.59-146.62)	461107 (306477-650990)	76.34 (50.74-107.78)	-1.49 (-1.61--1.38)
East Asia	109033 (65955-162387)	29.21 (17.67-43.5)	45666 (29221-66650)	19.27 (12.33-28.13)	-1.54 (-1.69--1.39)
East Asia & Pacific - WB	192470 (121894-281644)	34.05 (21.56-49.82)	122674 (78860-179535)	28.02 (18.01-41.01)	-0.91 (-1.08--0.73)
Eastern Europe	30030 (18999-44386)	63.53 (40.19-93.9)	19931 (12438-29721)	61.65 (38.48-91.94)	0.29 (0.11-0.46)

Eastern Mediterranean Region	83441 (57772-117018)	69.35 (48.02-97.26)	124449 (84159-177532)	58.9 (39.83-84.03)	-0.77 (-0.87--0.66)
Eastern Sub-Saharan Africa	42051 (28854-59422)	68.03 (46.68-96.13)	84719 (56722-120532)	60.65 (40.61-86.29)	-0.68 (-0.79--0.58)
Europe	61346 (38457-91721)	34.08 (21.36-50.95)	47020 (29137-69937)	33.1 (20.51-49.23)	0.14 (-0.1-0.37)
Europe & Central Asia - WB	66979 (42281-99369)	34.48 (21.76-51.15)	53607 (33257-79567)	33.48 (20.77-49.69)	0.1 (-0.11-0.32)
European Region	67136 (42374-99599)	34.22 (21.6-50.76)	53779 (33366-79828)	33.05 (20.5-49.05)	0.08 (-0.14-0.3)
High-income Asia Pacific	23803 (14633-35676)	56.46 (34.71-84.62)	15933 (10319-23282)	59.15 (38.31-86.44)	-0.04 (-0.14-0.06)
High-income North America	16793 (10002-24987)	27.55 (16.41-40.99)	19184 (13729-26202)	27.03 (19.34-36.92)	0 (-0.52-0.53)
Latin America & Caribbean - WB	28278 (18102-40931)	20.53 (13.14-29.72)	18688 (11759-27467)	11.6 (7.3-17.05)	-2.33 (-2.53--2.14)
Middle East & North Africa - WB	38660 (25504-56092)	46.73 (30.83-67.8)	50071 (31404-74290)	42.36 (26.57-62.85)	-0.4 (-0.45--0.34)
North Africa and Middle East	60785 (40770-87535)	55.04 (36.92-79.26)	85365 (55923-123750)	52.93 (34.67-76.73)	-0.1 (-0.25-0.06)
North America	16789 (9999-24983)	27.54 (16.41-40.99)	19182 (13727-26199)	27.03 (19.34-36.92)	0 (-0.52-0.53)
Oceania	2743 (1972-3635)	132.38 (95.14-175.42)	4022 (2785-5630)	103.18 (71.45-144.44)	-1.25 (-1.43--1.08)
Region of the Americas	44932 (27882-65911)	22.73 (14.11-33.34)	37762 (25488-53419)	16.32 (11.02-23.09)	-1.36 (-1.48--1.24)
South-East Asia Region	355919 (247583-492310)	88.88 (61.83-122.94)	342752 (220683-499878)	61.29 (39.46-89.38)	-1.71 (-1.84--1.57)
South Asia	362171 (252851-499941)	108.13 (75.49-149.26)	364096 (237221-526851)	69.25 (45.12-100.2)	-1.97 (-2.11--1.83)
South Asia - WB	368813 (258241-509667)	107.22 (75.07-148.16)	379997 (249249-547586)	69.84 (45.81-100.65)	-1.89 (-2.02--1.76)

Southeast Asia	57721 (39166-81596)	38.96 (26.43-55.07)	58004 (36424-85907)	34.15 (21.45-50.58)	-0.73 (-0.92--0.54)
Southern Latin America	1597 (928-2469)	12.07 (7.01-18.65)	1761 (1022-2693)	11.58 (6.72-17.7)	-0.21 (-0.26--0.16)
Southern Sub-Saharan Africa	7421 (4775-10864)	42.86 (27.58-62.75)	9330 (5958-13794)	43.76 (27.94-64.69)	0.13 (0.08-0.17)
Sub-Saharan Africa - WB	141922 (99816-195978)	87.28 (61.38-120.52)	314007 (215743-440096)	85.3 (58.61-119.56)	-0.67 (-0.88--0.46)
Tropical Latin America	15599 (9988-22795)	32.63 (20.89-47.68)	7120 (4465-10522)	13.64 (8.56-20.16)	-3.39 (-3.69--3.09)
Western Europe	7942 (4648-12386)	9.66 (5.66-15.07)	6810 (4122-10484)	9.51 (5.76-14.65)	0 (-0.06-0.05)
Western Pacific Region	160870 (100650-237318)	34.38 (21.51-50.72)	92892 (60462-135945)	27.72 (18.04-40.57)	-0.94 (-1.14--0.74)
Western Sub-Saharan Africa	78498 (54861-106389)	131.75 (92.07-178.56)	186904 (128943-258678)	123.93 (85.5-171.52)	-1.07 (-1.42--0.73)
World Bank High Income	59265 (36139-88408)	26.3 (16.04-39.23)	52684 (35401-74932)	25.43 (17.09-36.17)	-0.18 (-0.33--0.02)
World Bank Low Income	88203 (63827-120519)	85.27 (61.71-116.51)	173401 (121750-240497)	74.66 (52.42-103.55)	-1.41 (-1.73--1.08)
World Bank Lower Middle Income	505793 (351851-700949)	85.81 (59.69-118.91)	608374 (401715-868765)	66.33 (43.8-94.73)	-1.21 (-1.33--1.1)
World Bank Upper Middle Income	200771 (124697-297435)	31.88 (19.8-47.22)	123874 (77608-183758)	24.57 (15.39-36.45)	-0.93 (-1--0.86)
Countries					
Afghanistan	5372 (3953-7070)	141.63 (104.22-186.4)	14498 (10374-19734)	111.55 (79.82-151.83)	-1.24 (-1.83--0.65)
Albania	259 (156-385)	26.02 (15.69-38.76)	175 (102-264)	30.83 (18.02-46.54)	0.62 (0.47-0.76)
Algeria	3783 (2465-5532)	44.33 (28.88-64.82)	3968 (2422-5930)	40.61 (24.78-60.68)	-0.38 (-0.45--0.3)

American Samoa	12 (8-16)	77.89 (52.19-108.55)	12 (8-17)	68.8 (44.85-100.57)	-0.9 (-1.13--0.68)
Andorra	1 (1-2)	7.9 (4.48-12.62)	1 (1-2)	8.08 (4.65-12.95)	-0.19 (-0.27--0.12)
Angola	2130 (1489-2961)	66.39 (46.43-92.29)	5794 (3828-8265)	60.18 (39.76-85.84)	-0.41 (-0.57--0.24)
Antigua and Barbuda	3 (2-4)	14.91 (8.9-22.63)	3 (2-5)	16.4 (9.61-24.89)	0.2 (0.11-0.29)
Argentina	1087 (622-1666)	12.47 (7.14-19.11)	1239 (720-1896)	11.64 (6.76-17.81)	-0.21 (-0.29--0.12)
Armenia	339 (225-495)	38.9 (25.89-56.84)	184 (111-276)	33.83 (20.32-50.64)	-0.38 (-0.59--0.18)
Australia	349 (204-546)	8.79 (5.14-13.75)	399 (228-622)	8.78 (5.03-13.7)	0.02 (-0.06-0.1)
Austria	421 (274-616)	25.92 (16.84-37.88)	241 (150-363)	17.02 (10.63-25.66)	-1.61 (-1.77--1.45)
Azerbaijan	1019 (697-1428)	48.14 (32.9-67.47)	804 (492-1196)	36.47 (22.32-54.25)	-1.18 (-1.36--1)
Bahamas	12 (7-18)	14.41 (8.76-22.04)	15 (9-23)	16.07 (9.56-24.71)	0.23 (0.16-0.31)
Bahrain	50 (31-75)	38.5 (23.78-57.96)	99 (60-149)	40.01 (24.2-59.91)	0.26 (0.12-0.4)
Bangladesh	30361 (20950-41766)	86.28 (59.53-118.68)	32392 (20187-48664)	71.48 (44.55-107.39)	-0.98 (-1.28--0.67)
Barbados	9 (6-14)	14 (8.42-21.17)	8 (5-13)	14.52 (8.54-22.05)	-0.12 (-0.18--0.05)
Belarus	1429 (877-2150)	64.46 (39.58-96.97)	900 (544-1354)	63.83 (38.61-96.06)	0.37 (0.23-0.5)
Belgium	227 (133-357)	11.29 (6.61-17.74)	222 (130-344)	11.47 (6.68-17.74)	0.67 (0.36-0.97)
Belize	10 (6-15)	16.72 (10.31-24.75)	24 (15-37)	18.89 (11.27-28.6)	0.36 (0.34-0.39)

Benin	1674 (1173-2276)	116.01 (81.28-157.72)	4058 (2749-5703)	98.85 (66.98-138.93)	-0.88 (-0.98--0.78)
Bermuda	2 (1-3)	14.5 (8.62-22.04)	1 (1-2)	15.03 (8.88-23.01)	0.09 (0.02-0.15)
Bhutan	392 (297-507)	182.61 (138.33-236.39)	111 (70-164)	51.91 (32.79-76.75)	-5.47 (-6.11--4.82)
Bolivia (Plurinational State of)	592 (396-846)	29.59 (19.77-42.27)	538 (324-824)	16.58 (9.99-25.42)	-2.67 (-2.9--2.44)
Bosnia and Herzegovina	384 (239-574)	32.88 (20.48-49.18)	170 (102-257)	29.9 (17.93-45.18)	-0.33 (-0.44--0.21)
Botswana	224 (143-329)	49.64 (31.62-73.02)	279 (169-421)	42.83 (26.01-64.75)	0.08 (-0.31-0.47)
Brazil	15310 (9799-22347)	32.87 (21.04-47.98)	6802 (4270-10046)	13.54 (8.5-20)	-3.46 (-3.77--3.14)
Brunei Darussalam	41 (26-60)	53.77 (33.92-78.86)	59 (37-89)	55.09 (34.1-82.99)	0.11 (0-0.21)
Bulgaria	443 (269-663)	24.07 (14.59-36.03)	251 (149-372)	26.01 (15.45-38.64)	0.48 (0.35-0.62)
Burkina Faso	4057 (2941-5493)	137.35 (99.56-185.96)	9808 (6970-13406)	135.77 (96.48-185.57)	-0.21 (-0.33--0.1)
Burundi	1417 (999-1982)	84.58 (59.64-118.27)	2771 (1938-3858)	72.3 (50.56-100.67)	-0.42 (-0.52--0.33)
Cabo Verde	128 (86-176)	112.22 (75.54-154.43)	122 (77-177)	79.16 (49.73-115.08)	-1.42 (-1.75--1.08)
Cambodia	3223 (2387-4279)	100.72 (74.59-133.73)	1924 (1259-2760)	42.06 (27.53-60.33)	-3.72 (-3.97--3.48)
Cameroon	3869 (2773-5211)	120.09 (86.06-161.73)	9504 (6625-13313)	98.06 (68.36-137.36)	-0.93 (-1.04--0.82)
Canada	1601 (948-2382)	27.46 (16.27-40.87)	1730 (1018-2564)	27.37 (16.1-40.56)	0.12 (0.04-0.2)
Central African Republic	686 (496-937)	80.73 (58.33-110.18)	1357 (953-1869)	77.53 (54.45-106.8)	-0.21 (-0.25--0.18)

Chad	2813 (2055-3774)	154.23 (112.65-206.89)	8067 (5767-10905)	150.33 (107.46-203.21)	-0.24 (-0.51-0.03)
Chile	427 (249-668)	11.36 (6.63-17.77)	436 (251-667)	11.42 (6.56-17.47)	-0.25 (-0.59-0.09)
China	106427 (64280-158619)	29.4 (17.75-43.81)	43984 (28156-64109)	19.32 (12.37-28.16)	-1.55 (-1.71--1.4)
Colombia	1081 (666-1629)	10.57 (6.51-15.92)	1379 (864-2068)	11.66 (7.31-17.49)	0.94 (-0.15-2.04)
Comoros	111 (78-155)	72.22 (50.6-100.78)	136 (91-196)	63.12 (42.23-91.13)	-0.73 (-0.8--0.66)
Congo	457 (312-640)	56.23 (38.43-78.77)	802 (515-1166)	51.3 (32.97-74.61)	-0.41 (-0.48--0.35)
Cook Islands	4 (2-5)	60.04 (38.67-87.15)	3 (2-4)	64.38 (41.43-94.16)	0.12 (0.05-0.19)
Costa Rica	19 (9-34)	2.11 (0.98-3.72)	31 (16-53)	2.75 (1.42-4.71)	1.04 (0.79-1.3)
Croatia	287 (170-427)	27.75 (16.49-41.36)	145 (92-208)	21.34 (13.65-30.62)	-1.86 (-2.18--1.53)
Cuba	516 (302-786)	16.68 (9.78-25.43)	333 (196-501)	16.73 (9.85-25.16)	-0.1 (-0.24-0.05)
Cyprus	8 (4-13)	4.22 (2.31-6.97)	11 (6-17)	4.86 (2.63-7.9)	0.9 (0.7-1.09)
Czechia	370 (213-566)	15.56 (8.95-23.79)	295 (176-440)	19.41 (11.55-28.96)	0.5 (0.33-0.67)
C<U+00F4>te d'Ivoire	4679 (3376-6266)	122.29 (88.23-163.77)	9138 (6377-12421)	113.21 (79-153.88)	-0.48 (-0.62--0.35)
Democratic People's Republic of Korea	1609 (1045-2295)	29.11 (18.91-41.52)	1099 (680-1653)	20 (12.36-30.06)	-1.42 (-1.57--1.28)
Democratic Republic of the Congo	7103 (4866-9933)	59.13 (40.51-82.69)	17572 (11732-25530)	60.64 (40.49-88.11)	0.04 (-0.03-0.11)
Denmark	94 (54-149)	8.63 (4.93-13.67)	102 (59-161)	9.55 (5.52-15.07)	0.26 (0.1-0.43)
Djibouti	105 (72-151)	62.58 (42.66-89.66)	184 (119-265)	56.2 (36.38-80.86)	-0.29 (-0.34--0.23)

Dominica	4 (2-5)	15.83 (9.45-23.84)	3 (2-4)	16.57 (9.83-24.96)	0.09 (0.02-0.16)
Dominican Republic	464 (289-686)	19.5 (12.15-28.82)	554 (329-837)	19.12 (11.35-28.88)	-0.28 (-0.41--0.16)
Ecuador	665 (452-959)	20.38 (13.85-29.41)	558 (399-759)	11.36 (8.13-15.46)	-2.72 (-2.96--2.47)
Egypt	7273 (4689-10775)	42.38 (27.33-62.79)	11491 (7103-17249)	40.46 (25.01-60.73)	-0.19 (-0.26--0.12)
El Salvador	192 (121-284)	10.81 (6.82-15.98)	115 (66-181)	6.56 (3.76-10.28)	-2.47 (-2.84--2.1)
Equatorial Guinea	92 (65-126)	71.78 (50.78-98.1)	265 (167-392)	50.01 (31.47-73.96)	-1.73 (-1.95--1.52)
Eritrea	669 (473-925)	67.67 (47.84-93.65)	1517 (1045-2103)	67.2 (46.31-93.2)	-0.21 (-0.3--0.12)
Estonia	153 (94-234)	46.98 (28.74-71.61)	107 (67-160)	54.11 (33.68-80.83)	1.85 (1.22-2.48)
Eswatini	131 (85-195)	47.58 (30.81-71.01)	175 (110-261)	47.68 (30.11-71.33)	0.11 (0-0.23)
Ethiopia	13627 (9271-19346)	83.07 (56.51-117.92)	20990 (13758-30427)	56.29 (36.9-81.6)	-1.77 (-1.94--1.61)
Fiji	219 (150-299)	91.88 (62.81-125.4)	178 (118-253)	75.4 (50.09-107.13)	-1.3 (-1.49--1.11)
Finland	85 (50-131)	8.7 (5.16-13.48)	83 (48-129)	9.05 (5.24-14.01)	0.02 (-0.4-0.45)
France	977 (554-1583)	7.73 (4.38-12.52)	946 (553-1507)	7.83 (4.58-12.47)	0.05 (0-0.09)
Gabon	153 (101-218)	50.28 (33.23-71.69)	248 (154-371)	46.58 (29-69.72)	-0.36 (-0.46--0.26)
Gambia	364 (256-493)	116.03 (81.78-157.26)	734 (501-1030)	95.73 (65.37-134.36)	-0.84 (-0.98--0.7)
Georgia	479 (299-709)	37.32 (23.27-55.19)	285 (192-419)	45.5 (30.63-66.78)	0.97 (0.76-1.19)
Germany	1512 (864-2401)	10.15 (5.8-16.12)	1412 (828-2233)	11.05 (6.48-17.48)	1.39 (1.04-1.75)

Ghana	3866 (2594-5469)	82 (55.02-115.99)	9480 (6314-13528)	95.45 (63.58-136.2)	0.59 (0.51-0.68)
Greece	175 (98-280)	7.47 (4.2-11.97)	124 (72-198)	7.99 (4.65-12.78)	0.25 (0.12-0.37)
Greenland	5 (3-8)	36.97 (22.52-54.78)	3 (2-5)	27.57 (16.68-40.54)	-0.8 (-1.07--0.53)
Grenada	4 (2-6)	15.39 (9.15-23.27)	4 (3-7)	16.97 (10.03-26.14)	0.26 (0.2-0.31)
Guam	25 (16-37)	63.8 (40.13-94.53)	29 (18-42)	69 (43.16-100.43)	0.24 (0.06-0.42)
Guatemala	914 (634-1239)	36.71 (25.46-49.75)	756 (461-1128)	13.39 (8.16-19.97)	-4.51 (-5.36--3.65)
Guinea	2679 (1944-3620)	151.74 (110.1-205.03)	4704 (3337-6395)	115.86 (82.17-157.5)	-1.31 (-1.46--1.16)
Guinea-Bissau	546 (402-721)	168.98 (124.28-222.97)	833 (594-1125)	132.86 (94.68-179.33)	-1.01 (-1.17--0.85)
Guyana	68 (45-96)	26.37 (17.37-37.11)	50 (30-74)	22.41 (13.57-33.44)	-1.23 (-1.53--0.93)
Haiti	834 (582-1150)	42.97 (29.99-59.28)	883 (557-1313)	24.01 (15.15-35.7)	-2.5 (-2.7--2.3)
Honduras	366 (240-519)	23.9 (15.67-33.86)	277 (166-433)	8.83 (5.3-13.8)	-4.41 (-4.79--4.04)
Hungary	530 (315-790)	22.98 (13.64-34.24)	398 (238-596)	26.65 (15.9-39.85)	0.45 (0.35-0.55)
Iceland	4 (2-6)	5.55 (3.06-8.89)	4 (2-7)	6.49 (3.62-10.44)	0.61 (0.56-0.67)
India	284918 (197484-392840)	110.48 (76.58-152.33)	274573 (177981-397090)	69.1 (44.79-99.94)	-2.05 (-2.18--1.92)
Indonesia	14214 (8748-20968)	23.9 (14.71-35.25)	18644 (11279-27995)	27.46 (16.61-41.24)	0.32 (0.25-0.39)
Iran (Islamic Republic of)	11089 (7363-15874)	57.76 (38.35-82.68)	8191 (5159-12118)	46.53 (29.31-68.84)	-0.4 (-0.57--0.23)
Iraq	2100 (1313-3116)	36.89 (23.07-54.75)	5465 (3331-8258)	41.39 (25.23-62.54)	0.29 (0.14-0.44)

Ireland	70 (41-112)	7.19 (4.2-11.47)	73 (43-117)	7.68 (4.55-12.31)	0.18 (0.01-0.35)
Israel	38 (19-69)	2.76 (1.4-5.08)	65 (33-116)	3 (1.51-5.36)	-3.39 (-4.88--1.88)
Italy	1456 (850-2264)	11.63 (6.79-18.09)	917 (597-1341)	10.52 (6.86-15.39)	-0.89 (-1.09--0.7)
Jamaica	124 (77-185)	16.27 (10.02-24.25)	125 (75-190)	16.94 (10.07-25.61)	-0.1 (-0.18--0.02)
Japan	16227 (9842-24489)	57.74 (35.02-87.13)	10635 (7007-15109)	60.08 (39.59-85.36)	-0.17 (-0.29--0.05)
Jordan	391 (238-583)	28.57 (17.42-42.63)	1221 (777-1791)	34.46 (21.92-50.54)	0.73 (0.62-0.84)
Kazakhstan	1696 (1108-2449)	38.82 (25.36-56.06)	1313 (811-1941)	33.6 (20.77-49.68)	-0.46 (-0.57--0.36)
Kenya	4556 (3012-6580)	57.49 (38-83.02)	10125 (6693-14692)	59.05 (39.04-85.69)	-0.14 (-0.22--0.06)
Kiribati	79 (60-102)	354.23 (270.66-455.07)	81 (60-105)	232.47 (172.26-303.4)	-1.86 (-2.13--1.59)
Kuwait	168 (101-254)	35.36 (21.31-53.54)	326 (194-494)	40.68 (24.16-61.6)	0.57 (0.51-0.63)
Kyrgyzstan	419 (276-612)	32.44 (21.36-47.35)	536 (326-817)	31.61 (19.23-48.13)	0.26 (0.08-0.44)
Lao People's Democratic Republic	1894 (1440-2439)	146.28 (111.18-188.33)	1173 (812-1642)	55.69 (38.56-77.97)	-4.11 (-4.42--3.8)
Latvia	341 (208-501)	63.24 (38.53-92.81)	170 (103-255)	62.18 (37.55-93.09)	1.06 (0.69-1.44)
Lebanon	380 (236-568)	40.52 (25.2-60.5)	482 (294-729)	43.65 (26.6-66.04)	0.54 (0.39-0.69)
Lesotho	302 (197-441)	50.63 (32.91-73.77)	370 (241-541)	56.7 (36.91-83)	0.61 (0.49-0.73)
Liberia	938 (649-1284)	162.28 (112.19-222.06)	1789 (1209-2516)	112.73 (76.15-158.48)	-1.88 (-2.12--1.64)
Libya	514 (325-767)	35.94 (22.72-53.63)	729 (446-1075)	42.09 (25.79-62.12)	0.44 (0.37-0.51)

Lithuania	706 (431-1074)	86.25 (52.73-131.26)	382 (238-573)	88.21 (54.94-132.39)	0.45 (0.32-0.57)
Luxembourg	8 (5-13)	11.59 (6.7-18.51)	12 (7-19)	11.69 (6.82-17.9)	-0.54 (-0.69--0.38)
Madagascar	2568 (1824-3567)	67.07 (47.62-93.15)	5697 (3892-8064)	64.68 (44.19-91.55)	-0.43 (-0.59--0.26)
Malawi	1965 (1360-2758)	64.39 (44.55-90.38)	4280 (2945-6089)	64.4 (44.32-91.63)	-0.28 (-0.45--0.1)
Malaysia	1028 (605-1558)	19.11 (11.25-28.96)	2025 (1200-3030)	24.8 (14.7-37.1)	0.86 (0.8-0.92)
Maldives	18 (11-27)	25.41 (15.33-37.86)	36 (21-53)	34.96 (20.9-52.04)	1.52 (1.34-1.7)
Mali	4082 (2904-5470)	160.45 (114.15-215.01)	9163 (6378-12510)	125.57 (87.41-171.44)	-4.83 (-6.31--3.34)
Malta	3 (2-5)	4.07 (2.17-6.69)	3 (2-5)	4.84 (2.67-7.85)	0.99 (0.86-1.12)
Marshall Islands	25 (19-33)	164.64 (123.72-215)	17 (12-23)	101.41 (71.63-137.78)	-2.16 (-2.45--1.87)
Mauritania	711 (505-970)	111.76 (79.29-152.46)	1196 (786-1707)	88.8 (58.32-126.71)	-1.09 (-1.2--0.99)
Mauritius	68 (40-102)	20.95 (12.42-31.44)	64 (38-97)	23.71 (14.15-36.11)	0.16 (0.01-0.31)
Mexico	3004 (1774-4616)	10.32 (6.09-15.86)	2051 (1301-3055)	6.29 (3.99-9.36)	-2.61 (-2.93--2.29)
Micronesia (Federated States of)	65 (49-84)	188.62 (143.4-245.1)	31 (22-43)	97.89 (68.62-135.09)	-3.08 (-3.33--2.83)
Monaco	0 (0-1)	8.15 (4.58-13.05)	0 (0-1)	8.06 (4.65-12.66)	0.04 (-0.09-0.16)
Mongolia	323 (222-461)	45.68 (31.37-65.3)	310 (199-449)	42.44 (27.3-61.5)	-0.15 (-0.31-0)
Montenegro	39 (23-59)	24.91 (14.68-37.25)	34 (20-51)	28.32 (16.83-42.32)	0.47 (0.4-0.53)
Morocco	5250 (3529-7549)	64.76 (43.53-93.12)	4512 (2851-6679)	48.52 (30.66-71.82)	-1.34 (-1.5--1.18)

Mozambique	2567 (1794-3568)	62.94 (43.97-87.48)	7023 (4803-9756)	71.11 (48.63-98.78)	0.21 (0.08-0.34)
Myanmar	10558 (7659-14030)	82.32 (59.72-109.4)	5394 (3456-7976)	36.9 (23.64-54.56)	-3.62 (-3.88--3.37)
Namibia	227 (143-338)	47.79 (30.04-71.16)	326 (203-492)	44.61 (27.85-67.4)	-0.23 (-0.28--0.17)
Nauru	3 (2-4)	86.02 (59.59-117.63)	3 (2-4)	82.43 (55.68-115.81)	-0.34 (-0.68-0.01)
Nepal	7708 (5753-10145)	129.53 (96.67-170.47)	4705 (3171-6612)	49.26 (33.2-69.22)	-4.73 (-5.38--4.07)
Netherlands	264 (148-425)	8.06 (4.52-12.98)	247 (142-398)	8.15 (4.68-13.1)	0 (-0.09-0.1)
New Zealand	83 (48-131)	9.83 (5.68-15.61)	85 (57-122)	9.66 (6.44-13.76)	0.19 (-0.12-0.5)
Nicaragua	91 (54-146)	6.95 (4.14-11.15)	120 (69-195)	6.3 (3.64-10.28)	-0.72 (-0.84--0.61)
Niger	4514 (3279-6031)	184.6 (134.11-246.66)	9881 (6975-13488)	128.17 (90.46-174.94)	-5.57 (-7.09--4.03)
Nigeria	37644 (25739-52070)	133.7 (91.42-184.94)	97708 (66407-135647)	134.61 (91.49-186.87)	-0.09 (-0.27-0.09)
Niue	0 (0-1)	67.17 (43.52-95.64)	0 (0-0)	69.07 (43.97-101.41)	-0.08 (-0.17-0.01)
North Macedonia	122 (73-181)	23.5 (14.16-34.82)	107 (63-159)	28.1 (16.58-41.76)	0.76 (0.71-0.81)
Northern Mariana Islands	7 (4-11)	57.26 (35.37-84.54)	8 (5-11)	71.9 (44.25-106.28)	0.61 (0.39-0.84)
Norway	245 (146-378)	26.67 (15.9-41.22)	306 (182-477)	31.16 (18.59-48.59)	-0.7 (-1.15--0.25)
Oman	220 (136-323)	40.11 (24.76-58.84)	433 (258-656)	45.61 (27.19-69.15)	0.57 (0.49-0.65)
Pakistan	38791 (27387-53067)	108.66 (76.72-148.65)	52315 (35808-73826)	71.28 (48.79-100.59)	-1.85 (-1.99--1.71)
Palau	3 (2-4)	64.68 (41.15-93.82)	3 (2-4)	71.11 (44.92-106.66)	0.24 (0.15-0.32)

Palestine	297 (186-443)	43.9 (27.45-65.43)	663 (400-999)	41.15 (24.87-62.03)	-0.88 (-1.47--0.28)
Panama	39 (22-64)	5.11 (2.91-8.45)	60 (35-98)	5.6 (3.24-9.13)	0.18 (0.13-0.23)
Papua New Guinea	1751 (1249-2345)	133.85 (95.47-179.21)	3007 (2037-4259)	103.04 (69.8-145.96)	-1.27 (-1.43--1.1)
Paraguay	289 (178-431)	23.45 (14.42-34.93)	319 (190-479)	16.34 (9.74-24.57)	-1.21 (-1.35--1.07)
Peru	1525 (964-2260)	21.67 (13.7-32.11)	1351 (799-2042)	15.57 (9.21-23.53)	-1.57 (-1.77--1.37)
Philippines	15834 (10884-22455)	77.18 (53.05-109.45)	17047 (11119-25082)	53.18 (34.69-78.24)	-1.43 (-1.94--0.91)
Poland	3371 (2079-5130)	39.23 (24.2-59.72)	2211 (1597-3011)	37.67 (27.2-51.3)	0.29 (0.09-0.48)
Portugal	127 (71-210)	5.1 (2.84-8.43)	104 (59-169)	6.25 (3.52-10.15)	0.74 (0.61-0.88)
Puerto Rico	129 (76-195)	13.17 (7.7-19.89)	103 (61-157)	15.37 (9.12-23.25)	0.45 (0.41-0.5)
Qatar	47 (29-71)	47.6 (28.84-71.67)	301 (181-458)	59.04 (35.47-89.77)	1.1 (0.87-1.32)
Republic of Korea	7070 (4325-10482)	53.74 (32.88-79.68)	4823 (2982-7375)	57.58 (35.6-88.05)	0.26 (0.18-0.34)
Republic of Moldova	721 (461-1063)	70.08 (44.81-103.34)	436 (272-659)	71.5 (44.54-108.03)	0.36 (0.26-0.47)
Romania	1434 (855-2131)	24.28 (14.46-36.06)	793 (477-1166)	25.46 (15.33-37.44)	0.58 (0.38-0.79)
Russian Federation	19099 (12164-28250)	60.63 (38.61-89.68)	13293 (8318-19811)	58.29 (36.48-86.88)	0.26 (0.05-0.47)
Rwanda	2261 (1611-3132)	100.06 (71.29-138.58)	2992 (2070-4181)	71.34 (49.37-99.7)	-1.4 (-1.58--1.22)
Saint Kitts and Nevis	2 (1-3)	13.85 (8.43-21.06)	2 (1-3)	15.61 (9.22-23.69)	0.46 (0.41-0.5)
Saint Lucia	7 (4-10)	14.95 (9.04-22.42)	7 (4-10)	16.65 (9.79-24.97)	0.33 (0.25-0.41)
Saint Vincent and the Grenadines	6 (4-10)	17.28 (10.61-25.5)	5 (3-7)	17.46 (10.53-26.04)	-0.2 (-0.27--0.13)

Samoa	50 (34-69)	85.06 (57.76-116.72)	50 (33-71)	72.59 (48.55-102.85)	-1.05 (-1.19--0.91)
San Marino	0 (0-1)	7.61 (4.23-12.31)	0 (0-1)	8.05 (4.62-12.84)	0.09 (0.05-0.14)
Sao Tome and Principe	44 (31-60)	109.76 (76.77-149.41)	53 (34-75)	78.03 (50.88-111.74)	-1.82 (-2.11--1.53)
Saudi Arabia	1948 (1214-2918)	38.3 (23.86-57.38)	3469 (2062-5279)	41.9 (24.91-63.76)	0.42 (0.33-0.51)
Senegal	2947 (2134-3969)	121.83 (88.24-164.07)	4744 (3282-6646)	94.86 (65.63-132.89)	-1.13 (-1.21--1.05)
Serbia	389 (229-584)	18.58 (10.92-27.86)	373 (220-558)	23.76 (14.02-35.53)	0.46 (0.35-0.57)
Seychelles	13 (10-18)	60.13 (42.84-82.53)	9 (6-13)	43.24 (28.91-62.83)	-1.46 (-1.61--1.31)
Sierra Leone	1533 (1094-2086)	143.19 (102.12-194.8)	3465 (2414-4739)	126 (87.78-172.34)	-0.65 (-0.79--0.52)
Singapore	464 (284-699)	56.27 (34.38-84.74)	416 (252-621)	55.38 (33.53-82.81)	0.03 (-0.03-0.09)
Slovakia	381 (229-572)	29.91 (18.02-44.91)	287 (170-434)	34.18 (20.21-51.62)	-0.41 (-0.66--0.17)
Slovenia	97 (56-145)	21.92 (12.72-32.8)	71 (41-105)	24.3 (14.11-36.2)	-0.66 (-0.97--0.34)
Solomon Islands	188 (141-248)	163.16 (122.06-214.64)	237 (171-322)	116.85 (84.26-158.74)	-1.57 (-1.78--1.36)
Somalia	1351 (940-1907)	58.98 (41.01-83.26)	5067 (3452-7254)	73.97 (50.4-105.91)	0.37 (0.06-0.69)
South Africa	4550 (2825-6744)	38.15 (23.69-56.55)	5342 (3270-7990)	38.09 (23.32-56.97)	0.13 (0.04-0.21)
South Sudan	1121 (753-1601)	56.04 (37.64-80.02)	1840 (1226-2672)	56.91 (37.92-82.64)	-0.08 (-0.16-0)
Spain	743 (424-1196)	7.63 (4.35-12.28)	539 (300-858)	7.8 (4.34-12.42)	-0.01 (-0.15-0.12)

Sri Lanka	1252 (753-1915)	24.15 (14.53-36.95)	1367 (797-2064)	26.52 (15.45-40.03)	0.27 (0.22-0.32)
Sudan	3388 (2302-4890)	52.51 (35.68-75.79)	7197 (4640-10730)	53.51 (34.5-79.78)	-0.1 (-0.21-0)
Suriname	26 (16-38)	21.57 (13.65-31.55)	24 (14-36)	17.05 (10.2-25.37)	-1.08 (-1.15--1)
Sweden	210 (122-326)	12.7 (7.36-19.71)	236 (139-362)	13.66 (8.03-20.96)	0.4 (0.27-0.53)
Switzerland	114 (65-177)	8.58 (4.89-13.29)	118 (69-185)	8.69 (5.1-13.64)	0.1 (0.03-0.17)
Syrian Arab Republic	715 (420-1079)	16.4 (9.64-24.76)	1158 (684-1738)	23.75 (14.01-35.64)	0.77 (0.26-1.28)
Taiwan (Province of China)	997 (595-1489)	17.44 (10.41-26.05)	583 (335-875)	15.45 (8.87-23.19)	-0.53 (-0.64--0.42)
Tajikistan	859 (586-1192)	51.78 (35.36-71.9)	1065 (680-1578)	39.46 (25.21-58.48)	-1.31 (-1.47--1.14)
Thailand	4674 (2822-7096)	26.13 (15.78-39.68)	4210 (2512-6445)	32.08 (19.14-49.11)	0.66 (0.58-0.74)
Timor-Leste	215 (158-287)	93.45 (68.62-124.87)	221 (153-308)	48.34 (33.51-67.48)	-2.88 (-3.06--2.7)
Togo	1406 (990-1919)	117.92 (83.05-160.93)	2454 (1686-3457)	99.39 (68.28-140.01)	-0.86 (-0.94--0.77)
Tokelau	0 (0-0)	71.49 (46.82-102.24)	0 (0-0)	69.79 (44.43-100.6)	-0.29 (-0.41--0.17)
Tonga	33 (22-45)	97.41 (65.67-135.57)	24 (16-34)	78.85 (51.35-111.88)	-1.2 (-1.34--1.05)
Trinidad and Tobago	51 (31-76)	14.95 (8.92-22.19)	43 (26-65)	15.72 (9.43-23.7)	0.26 (0.05-0.47)
Tunisia	1073 (660-1592)	40.15 (24.7-59.57)	1036 (624-1549)	41.7 (25.11-62.35)	0.29 (0.23-0.35)
Turkmenistan	462 (307-660)	40.23 (26.71-57.44)	554 (356-807)	44.2 (28.37-64.34)	0.35 (0.08-0.63)
Tuvalu	2 (2-3)	91.41 (62.58-126.38)	3 (2-4)	74.61 (49.11-105.58)	-1.07 (-1.23--0.92)
Türkiye	13470 (8574-20341)	70.91 (45.14-107.08)	13765 (8366-20739)	72.34 (43.97-108.99)	0.6 (0.22-0.97)

Uganda	3476 (2377-4926)	61.49 (42.04-87.13)	8661 (5825-12441)	60.15 (40.46-86.4)	-0.34 (-0.44--0.24)
Ukraine	7581 (4756-11170)	69.95 (43.88-103.07)	4643 (2911-6944)	70.34 (44.1-105.21)	0.31 (0.21-0.4)
United Arab Emirates	153 (92-234)	36.29 (21.83-55.44)	398 (241-599)	37.55 (22.72-56.57)	0.46 (0.09-0.83)
United Kingdom	1152 (640-1848)	9.73 (5.41-15.61)	1035 (649-1555)	8.79 (5.51-13.2)	-0.77 (-1--0.54)
United Republic of Tanzania	4216 (2773-6124)	49.5 (32.56-71.9)	9774 (6255-14396)	53.28 (34.09-78.47)	0.1 (0.03-0.17)
United States of America	15186 (9008-22678)	27.56 (16.35-41.15)	17450 (12709-23666)	27 (19.66-36.62)	0 (-0.58-0.57)
United States Virgin Islands	4 (2-6)	13.39 (7.89-20.64)	3 (2-4)	14.88 (8.58-22.69)	0.3 (0.23-0.36)
Uruguay	84 (48-130)	10.95 (6.27-16.96)	85 (50-131)	11.43 (6.73-17.65)	0.1 (0.06-0.14)
Uzbekistan	2322 (1513-3305)	36.33 (23.68-51.72)	3229 (2018-4759)	36.07 (22.54-53.17)	-0.2 (-0.36--0.04)
Vanuatu	125 (95-160)	264.76 (201.44-339.72)	148 (109-195)	165.52 (122.04-217.96)	-2.1 (-2.39--1.81)
Venezuela (Bolivarian Republic of)	319 (186-505)	5.32 (3.1-8.43)	382 (219-625)	5.69 (3.26-9.32)	0.15 (0.06-0.24)
Viet Nam	4652 (2730-7079)	21.58 (12.67-32.85)	5814 (3436-8769)	27.57 (16.29-41.58)	1.06 (0.99-1.14)
Yemen	3063 (2147-4230)	72.54 (50.84-100.16)	5877 (3943-8441)	57.82 (38.8-83.05)	-1.2 (-1.43--0.97)
Zambia	2009 (1380-2812)	72.43 (49.76-101.4)	3596 (2372-5229)	58.41 (38.53-84.93)	-1.02 (-1.17--0.87)
Zimbabwe	1987 (1353-2831)	55.36 (37.69-78.87)	2840 (1922-4061)	57.97 (39.23-82.9)	0.07 (0.03-0.11)

Supplementary Table 3：Global Cases and Age-Standardized DALYS of Gastrointestinal Ulcers Among Individuals Aged 10–24 Years in 2019

	Number 1990	ASR 1990	Number2019	ASR 2019	EAPC_CI
Global	613694 (525005-706071)	39.62 (33.89-45.58)	363862 (309793-422230)	19.54 (16.64-22.68)	-2.81 (-2.96--2.66)
Sex					
Female	309860 (242821-387266)	40.67 (31.87-50.83)	184013 (152388-221044)	20.26 (16.78-24.33)	-2.89 (-3.1--2.68)
Male	303834 (264301-353441)	38.6 (33.58-44.9)	179849 (153402-210824)	18.86 (16.09-22.11)	-2.73 (-2.83--2.63)
SDI regions					
High SDI	10688 (9207-12670)	5.83 (5.02-6.91)	4178 (3063-5895)	2.38 (1.75-3.36)	-3.22 (-3.49--2.95)
High-middle SDI	43311 (38135-49339)	14.31 (12.6-16.3)	15158 (12510-18601)	5.97 (4.93-7.33)	-3.36 (-3.52--3.21)
Middle SDI	137442 (120962-165389)	25.25 (22.22-30.38)	58453 (49447-70885)	10.64 (9-12.9)	-3.05 (-3.15--2.95)
Low-middle SDI	294191 (244207-346540)	83.13 (69.01-97.92)	145729 (123570-171123)	28.55 (24.21-33.53)	-4.18 (-4.47--3.9)
Low SDI	127756 (101556-153787)	77.97 (61.98-93.86)	140083 (115759-165841)	37.71 (31.16-44.64)	-2.8 (-2.94--2.65)
GBD region					
Africa	107905 (88738-130700)	53.77 (44.22-65.13)	152011 (120908-188160)	36.18 (28.78-44.79)	-1.37 (-1.42--1.31)
African Region	96751 (78723-118362)	59.57 (48.47-72.88)	140786 (111186-175365)	39.37 (31.09-49.04)	-1.44 (-1.51--1.37)

America	32863 (30591-35650)	16.62 (15.48-18.03)	15761 (13453-18431)	6.81 (5.81-7.97)	-3.15 (-3.38--2.92)
Andean Latin America	5126 (4268-6200)	41.67 (34.7-50.4)	1864 (1397-2404)	11.08 (8.3-14.28)	-4.81 (-5.03--4.58)
Asia	455144 (383992-539159)	46.99 (39.64-55.66)	188875 (157000-228469)	17.71 (14.72-21.42)	-3.91 (-4.18--3.64)
Australasia	132 (108-162)	2.74 (2.23-3.36)	44 (30-63)	0.81 (0.56-1.17)	-4.3 (-4.8--3.79)
Caribbean	3002 (2219-3981)	28.19 (20.84-37.38)	1918 (1358-2579)	16.74 (11.86-22.51)	-2.02 (-2.36--1.67)
Central Asia	4625 (4114-5188)	23.31 (20.74-26.15)	3748 (3110-4601)	16.57 (13.75-20.35)	-2.37 (-2.83--1.9)
Central Europe	2710 (2414-3090)	9.44 (8.41-10.77)	773 (617-986)	4.3 (3.43-5.48)	-2.65 (-2.84--2.46)
Central Latin America	13728 (12710-14804)	25.37 (23.49-27.36)	6684 (5408-8217)	10.16 (8.22-12.49)	-3.32 (-3.52--3.11)
Central Sub-Saharan Africa	11561 (8320-15421)	66.78 (48.05-89.07)	15848 (10902-22936)	36.87 (25.37-53.37)	-1.82 (-1.94--1.7)
Commonwealth High Income	1169 (1039-1341)	4.83 (4.3-5.54)	448 (339-600)	1.78 (1.35-2.39)	-3.82 (-4.03--3.61)
Commonwealth Low Income	35193 (27044-45138)	51.95 (39.92-66.63)	35284 (26885-44955)	29.73 (22.66-37.88)	-1.94 (-2.21--1.66)
Commonwealth Middle Income	316803 (261668-376856)	87.96 (72.65-104.63)	175958 (144745-215372)	29.13 (23.96-35.66)	-4.33 (-4.7--3.96)
East Asia	78684 (65110-96098)	21.08 (17.44-25.74)	11439 (9559-13791)	4.83 (4.03-5.82)	-5.29 (-5.44--5.14)
East Asia & Pacific - WB	130339 (110806-164124)	23.06 (19.6-29.03)	37181 (31261-47835)	8.49 (7.14-10.93)	-3.65 (-3.85--3.46)
Eastern Europe	6972 (6032-8251)	14.75 (12.76-17.46)	3459 (2817-4323)	10.7 (8.72-13.37)	-1.64 (-2.06--1.23)

Eastern Mediterranean Region	34812 (28085-42340)	28.93 (23.34-35.19)	37378 (29828-46468)	17.69 (14.12-21.99)	-1.77 (-1.85--1.69)
Eastern Sub-Saharan Africa	40669 (30221-54349)	65.79 (48.89-87.92)	49796 (38975-61877)	35.65 (27.9-44.3)	-2.33 (-2.46--2.2)
Europe	17386 (15249-20005)	9.66 (8.47-11.11)	6929 (5595-8763)	4.88 (3.94-6.17)	-2.82 (-3.08--2.56)
Europe & Central Asia - WB	20669 (18304-23559)	10.64 (9.42-12.13)	10052 (8340-12252)	6.28 (5.21-7.65)	-2.38 (-2.69--2.07)
European Region	20737 (18359-23624)	10.57 (9.36-12.04)	10080 (8362-12286)	6.19 (5.14-7.55)	-2.41 (-2.72--2.1)
High-income Asia Pacific	3791 (3060-4807)	8.99 (7.26-11.4)	1066 (693-1581)	3.96 (2.57-5.87)	-2.76 (-3.1--2.43)
High-income North America	2405 (1948-3013)	3.95 (3.2-4.94)	1523 (1088-2163)	2.15 (1.53-3.05)	-2.29 (-2.77--1.82)
Latin America & Caribbean - WB	30532 (28409-33048)	22.17 (20.62-23.99)	14252 (12138-16663)	8.85 (7.54-10.34)	-3.2 (-3.42--2.98)
Middle East & North Africa - WB	19956 (16164-24130)	24.12 (19.54-29.17)	11866 (9281-15547)	10.04 (7.85-13.15)	-2.87 (-2.94--2.79)
North Africa and Middle East	28685 (22538-35111)	25.97 (20.41-31.79)	21218 (16636-26910)	13.16 (10.32-16.69)	-2.23 (-2.29--2.16)
North America	2405 (1948-3012)	3.95 (3.2-4.94)	1523 (1088-2163)	2.15 (1.53-3.05)	-2.29 (-2.77--1.82)
Oceania	1609 (1247-2088)	77.64 (60.16-100.73)	2177 (1538-3182)	55.86 (39.46-81.64)	-1.19 (-1.26--1.13)
Region of the Americas	32863 (30591-35650)	16.62 (15.48-18.03)	15761 (13453-18431)	6.81 (5.81-7.97)	-3.15 (-3.38--2.92)
South-East Asia Region	324613 (263371-392308)	81.06 (65.77-97.97)	130907 (102867-160227)	23.41 (18.39-28.65)	-4.9 (-5.32--4.49)
South Asia	307772 (251098-369240)	91.89 (74.97-110.24)	136641 (109681-165836)	25.99 (20.86-31.54)	-4.97 (-5.37--4.56)

South Asia - WB	311878 (254291-374340)	90.66 (73.92-108.82)	142506 (114706-172300)	26.19 (21.08-31.67)	-4.88 (-5.27--4.48)
Southeast Asia	46523 (36063-66504)	31.4 (24.34-44.89)	22578 (18267-32010)	13.29 (10.75-18.85)	-3.06 (-3.17--2.95)
Southern Latin America	781 (672-912)	5.9 (5.08-6.89)	480 (383-603)	3.15 (2.52-3.97)	-1.97 (-2.29--1.64)
Southern Sub-Saharan Africa	3471 (2485-4670)	20.05 (14.35-26.97)	4228 (3041-5910)	19.83 (14.26-27.72)	0.33 (-0.17-0.83)
Sub-Saharan Africa - WB	97581 (79316-118967)	60.01 (48.78-73.16)	146208 (116473-181065)	39.72 (31.64-49.19)	-1.46 (-1.54--1.37)
Tropical Latin America	7997 (7251-8815)	16.73 (15.17-18.44)	3372 (2986-3854)	6.46 (5.72-7.39)	-2.93 (-3.17--2.7)
Western Europe	3640 (3308-4014)	4.43 (4.03-4.88)	940 (752-1206)	1.31 (1.05-1.68)	-4.64 (-4.87--4.4)
Western Pacific Region	103309 (87599-125326)	22.08 (18.72-26.79)	28524 (24274-36003)	8.51 (7.24-10.74)	-3.42 (-3.66--3.18)
Western Sub-Saharan Africa	39813 (30807-53630)	66.82 (51.71-90.01)	74066 (53625-101231)	49.11 (35.56-67.12)	-1.06 (-1.15--0.97)
World Bank High Income	13045 (11361-15269)	5.79 (5.04-6.78)	4857 (3616-6701)	2.34 (1.75-3.23)	-3.21 (-3.47--2.94)
World Bank Low Income	65885 (50290-82810)	63.7 (48.62-80.06)	91432 (72182-111309)	39.37 (31.08-47.93)	-1.82 (-1.9--1.75)
World Bank Lower Middle Income	409799 (343444-480775)	69.52 (58.26-81.56)	235934 (197382-282710)	25.73 (21.52-30.83)	-3.84 (-4.1--3.58)
World Bank Upper Middle Income	124659 (108515-145096)	19.79 (17.23-23.04)	31378 (27092-36555)	6.22 (5.37-7.25)	-4.15 (-4.27--4.03)
Countries					
Afghanistan	3553 (2034-5291)	93.67 (53.62-139.5)	5705 (3699-8336)	43.89 (28.46-64.14)	-2.79 (-3.13--2.45)
Albania	85 (65-114)	8.57 (6.51-11.51)	22 (14-32)	3.83 (2.46-5.69)	-3.28 (-3.9--2.65)

Algeria	2575 (1647-3768)	30.17 (19.3-44.15)	942 (654-1319)	9.64 (6.69-13.5)	-3.82 (-3.95--3.7)
American Samoa	6 (4-9)	41.76 (29.54-57.74)	4 (3-5)	22.39 (16.09-30.79)	-2.62 (-2.85--2.39)
Andorra	0 (0-0)	1.14 (0.74-1.66)	0 (0-0)	0.69 (0.43-1.02)	-2.01 (-2.15--1.87)
Angola	2307 (1287-3551)	71.93 (40.14-110.69)	3197 (1992-4801)	33.21 (20.69-49.87)	-2.38 (-2.58--2.19)
Antigua and Barbuda	1 (1-2)	8.11 (6.06-10.46)	1 (1-1)	4.46 (3.2-6.12)	-3.55 (-4.12--2.98)
Argentina	507 (417-618)	5.82 (4.78-7.09)	379 (291-491)	3.56 (2.74-4.62)	-1.43 (-1.79--1.07)
Armenia	97 (75-124)	11.11 (8.63-14.2)	45 (32-61)	8.19 (5.94-11.25)	-1.9 (-3--0.78)
Australia	104 (82-131)	2.62 (2.06-3.3)	35 (24-52)	0.78 (0.52-1.15)	-4.36 (-4.86--3.85)
Austria	75 (58-95)	4.61 (3.59-5.88)	19 (12-30)	1.36 (0.86-2.1)	-4.81 (-5.17--4.44)
Azerbaijan	570 (434-724)	26.94 (20.5-34.18)	302 (212-439)	13.72 (9.63-19.9)	-4.41 (-5.23--3.57)
Bahamas	12 (9-16)	14.62 (10.85-19.58)	8 (6-12)	8.92 (6.23-12.59)	-1.44 (-1.77--1.12)
Bahrain	20 (14-26)	15.19 (11.17-19.9)	17 (11-25)	6.76 (4.47-10.07)	-3.25 (-3.63--2.86)
Bangladesh	19863 (13581-29037)	56.44 (38.59-82.51)	8561 (4463-12826)	18.89 (9.85-28.3)	-4.4 (-5.35--3.44)
Barbados	10 (8-13)	15.09 (11.36-19.51)	4 (3-5)	6.81 (4.78-9.58)	-3.65 (-3.99--3.32)
Belarus	210 (158-279)	9.47 (7.14-12.6)	80 (51-113)	5.66 (3.63-8.05)	-1.98 (-2.17--1.79)

Belgium	104 (84-128)	5.17 (4.2-6.35)	29 (21-40)	1.52 (1.1-2.05)	-4.61 (-4.84--4.37)
Belize	6 (5-8)	10.26 (7.37-13.53)	10 (7-13)	7.47 (5.37-10.28)	-2.41 (-2.96--1.85)
Benin	942 (615-1445)	65.29 (42.61-100.18)	2057 (1258-3178)	50.12 (30.65-77.42)	-0.83 (-0.95--0.72)
Bermuda	2 (1-2)	12.68 (9.58-16.79)	0 (0-0)	3.54 (2.59-4.8)	-4.68 (-5.08--4.27)
Bhutan	173 (91-263)	80.7 (42.39-122.72)	52 (28-91)	24.09 (12.87-42.72)	-4.78 (-5.09--4.47)
Bolivia (Plurinational State of)	1029 (620-1528)	51.41 (30.96-76.31)	453 (299-657)	13.96 (9.22-20.27)	-4.75 (-4.95--4.55)
Bosnia and Herzegovina	99 (74-133)	8.44 (6.32-11.42)	24 (16-34)	4.23 (2.87-6.04)	-3.36 (-3.99--2.73)
Botswana	114 (69-179)	25.3 (15.33-39.61)	118 (70-183)	18.16 (10.7-28.14)	-1.37 (-1.8--0.94)
Brazil	7837 (7107-8649)	16.83 (15.26-18.57)	3247 (2881-3692)	6.46 (5.73-7.35)	-2.93 (-3.17--2.69)
Brunei Darussalam	16 (11-22)	21.08 (14.37-29.36)	6 (4-8)	5.34 (3.57-7.69)	-5.44 (-6.04--4.84)
Bulgaria	203 (164-256)	11.05 (8.89-13.88)	51 (35-74)	5.33 (3.64-7.69)	-2.49 (-2.76--2.21)
Burkina Faso	1132 (730-1622)	38.34 (24.72-54.92)	2752 (1639-4455)	38.09 (22.69-61.66)	0.23 (0.11-0.35)
Burundi	1666 (990-2725)	99.44 (59.08-162.59)	2338 (1477-3625)	61.01 (38.54-94.59)	-1.62 (-1.81--1.43)
Cabo Verde	74 (52-101)	64.53 (45.61-88.27)	25 (17-33)	16.12 (11.33-21.48)	-4.72 (-5.17--4.27)

Cambodia	5703 (3663-8137)	178.21 (114.46-254.29)	2385 (1476-3881)	52.12 (32.26-84.82)	-4.54 (-4.72--4.37)
Cameroon	1933 (1363-2664)	60 (42.29-82.68)	4151 (2360-6336)	42.83 (24.35-65.37)	-1.14 (-1.39--0.89)
Canada	228 (168-306)	3.92 (2.88-5.25)	131 (80-207)	2.08 (1.27-3.28)	-2.22 (-2.57--1.87)
Central African Republic	1031 (674-1498)	121.31 (79.33-176.21)	1481 (834-2375)	84.63 (47.64-135.7)	-1.37 (-1.47--1.27)
Chad	1551 (1045-2214)	85.01 (57.27-121.39)	3987 (2636-5571)	74.29 (49.12-103.82)	-0.28 (-0.6-0.04)
Chile	231 (180-292)	6.14 (4.81-7.77)	80 (60-106)	2.1 (1.56-2.77)	-3.57 (-3.77--3.37)
China	76407 (62668-94039)	21.1 (17.31-25.97)	10370 (8437-12669)	4.56 (3.71-5.56)	-5.51 (-5.66--5.36)
Colombia	2290 (1856-2720)	22.39 (18.15-26.59)	589 (406-821)	4.99 (3.44-6.94)	-5.13 (-5.57--4.69)
Comoros	82 (15-152)	53.14 (9.7-98.99)	77 (36-130)	35.82 (16.59-60.44)	-2.42 (-3.38--1.45)
Congo	527 (317-825)	64.91 (39.01-101.64)	392 (228-630)	25.11 (14.6-40.35)	-3.33 (-3.59--3.07)
Cook Islands	1 (1-1)	14.36 (9.24-20.26)	0 (0-0)	6.46 (4.27-9.64)	-3.01 (-3.16--2.85)
Costa Rica	52 (41-64)	5.61 (4.49-6.92)	43 (30-63)	3.86 (2.64-5.62)	-2.01 (-2.47--1.55)
Croatia	65 (49-82)	6.25 (4.79-7.97)	18 (12-25)	2.6 (1.77-3.66)	-4.08 (-4.37--3.78)
Cuba	306 (239-380)	9.88 (7.72-12.29)	64 (45-87)	3.23 (2.28-4.38)	-4.6 (-5.18--4.02)

Cyprus	4 (3-5)	1.89 (1.32-2.55)	2 (1-3)	0.84 (0.55-1.16)	-3.36 (-3.64--3.08)
Czechia	110 (88-135)	4.63 (3.71-5.69)	35 (24-48)	2.3 (1.6-3.18)	-2.39 (-2.57--2.21)
C<U+00F4>te d'Ivoire	2571 (1726-3650)	67.2 (45.1-95.4)	4093 (2553-6131)	50.71 (31.62-75.96)	-1.11 (-1.47--0.75)
Democratic People's Republic of Korea	1691 (935-2775)	30.6 (16.92-50.21)	980 (590-1506)	17.83 (10.72-27.39)	-1.65 (-1.71--1.59)
Democratic Republic of the Congo	7432 (4903-10528)	61.88 (40.82-87.65)	10571 (6733-16435)	36.48 (23.24-56.72)	-1.53 (-1.68--1.37)
Denmark	46 (38-55)	4.24 (3.51-5.04)	19 (15-25)	1.81 (1.38-2.31)	-4.05 (-4.62--3.48)
Djibouti	75 (40-128)	44.34 (23.62-76.08)	86 (45-140)	26.36 (13.64-42.61)	-1.72 (-2.11--1.33)
Dominica	3 (2-4)	13.49 (10.12-17.97)	1 (1-2)	8.52 (5.93-11.9)	-1.58 (-1.95--1.2)
Dominican Republic	858 (668-1121)	36.04 (28.07-47.1)	592 (340-903)	20.4 (11.74-31.14)	-2.13 (-2.82--1.43)
Ecuador	1277 (1062-1507)	39.15 (32.57-46.19)	516 (369-697)	10.52 (7.53-14.21)	-4.94 (-5.24--4.63)
Egypt	4489 (3257-5832)	26.16 (18.98-33.99)	3214 (1809-5468)	11.32 (6.37-19.25)	-2.1 (-2.51--1.69)
El Salvador	797 (643-970)	44.82 (36.18-54.54)	179 (115-276)	10.21 (6.57-15.73)	-5.7 (-6.33--5.07)
Equatorial Guinea	119 (71-183)	92.98 (55.22-142.56)	96 (47-163)	18.08 (8.87-30.76)	-6.31 (-6.79--5.82)
Eritrea	681 (389-1087)	68.91 (39.42-109.97)	1160 (689-1847)	51.39 (30.54-81.84)	-1.1 (-1.3--0.9)
Estonia	27 (20-35)	8.18 (6.15-10.58)	8 (5-12)	4.13 (2.58-6.2)	-1.91 (-2.29--1.54)

Eswatini	57 (35-91)	20.77 (12.65-33.18)	89 (52-147)	24.19 (14.13-40.17)	1.27 (0.49-2.06)
Ethiopia	16706 (10840-24138)	101.84 (66.08-147.13)	10215 (6908-14221)	27.39 (18.53-38.14)	-4.98 (-5.2--4.76)
Fiji	139 (93-197)	58.21 (39.19-82.63)	90 (60-130)	38.1 (25.59-54.92)	-1.37 (-1.53--1.21)
Finland	54 (42-68)	5.54 (4.33-6.95)	18 (14-24)	2.02 (1.49-2.66)	-3.61 (-3.83--3.38)
France	443 (364-528)	3.5 (2.88-4.18)	110 (78-152)	0.91 (0.65-1.26)	-5.09 (-5.5--4.67)
Gabon	145 (88-223)	47.44 (29-73.12)	110 (63-185)	20.69 (11.91-34.79)	-2.86 (-2.97--2.75)
Gambia	208 (127-322)	66.53 (40.63-102.85)	330 (211-476)	43.03 (27.51-62.06)	-1.6 (-1.93--1.27)
Georgia	259 (195-335)	20.13 (15.19-26.12)	69 (50-95)	11.02 (7.91-15.12)	-1.93 (-2.69--1.16)
Germany	670 (536-830)	4.5 (3.6-5.57)	230 (170-303)	1.8 (1.33-2.37)	-3.31 (-3.48--3.13)
Ghana	1494 (996-2171)	31.69 (21.12-46.04)	2192 (1444-3051)	22.07 (14.54-30.72)	-0.8 (-0.98--0.62)
Greece	71 (57-88)	3.03 (2.43-3.75)	38 (29-50)	2.44 (1.86-3.19)	-0.69 (-0.95--0.42)
Greenland	2 (1-3)	14.93 (9.49-21.37)	1 (0-1)	6.07 (3.88-9.68)	-3.67 (-4.24--3.1)
Grenada	4 (3-6)	16.68 (12.52-21.26)	2 (2-3)	8.28 (5.91-11.24)	-2.1 (-2.39--1.81)
Guam	8 (6-12)	20.73 (14.21-30.67)	4 (3-6)	10.77 (7.5-14.67)	-2.53 (-2.82--2.23)
Guatemala	2550 (2022-3238)	102.37 (81.19-130.01)	2319 (1618-3189)	41.06 (28.65-56.46)	-3.82 (-4.25--3.38)
Guinea	1350 (900-1916)	76.48 (50.98-108.54)	2403 (1462-3506)	59.19 (36.01-86.33)	-0.5 (-0.63--0.38)

Guinea-Bissau	414 (273-605)	128.14 (84.58-187.36)	504 (346-732)	80.44 (55.16-116.67)	-1.22 (-1.44--1)
Guyana	115 (86-151)	44.54 (32.98-58.29)	54 (36-79)	24.39 (16.33-35.47)	-1.95 (-2.3--1.6)
Haiti	1299 (633-2164)	66.98 (32.62-111.57)	979 (537-1532)	26.62 (14.61-41.65)	-3.02 (-3.28--2.75)
Honduras	1423 (1023-1941)	92.87 (66.74-126.66)	650 (402-985)	20.71 (12.8-31.4)	-5.41 (-5.62--5.2)
Hungary	207 (165-258)	8.97 (7.14-11.18)	53 (37-74)	3.57 (2.46-4.93)	-3.32 (-3.5--3.14)
Iceland	2 (2-3)	3.31 (2.56-4.22)	1 (0-1)	0.86 (0.59-1.17)	-4.76 (-5.09--4.43)
India	276484 (221796-339200)	107.21 (86.01-131.53)	113184 (88063-140826)	28.49 (22.16-35.44)	-5.22 (-5.7--4.74)
Indonesia	8566 (6292-14221)	14.4 (10.58-23.91)	3008 (2169-4442)	4.43 (3.19-6.54)	-4.41 (-4.58--4.23)
Iran (Islamic Republic of)	5543 (4487-7120)	28.87 (23.37-37.08)	1932 (1596-2367)	10.97 (9.06-13.45)	-2.87 (-3.05--2.68)
Iraq	927 (539-1499)	16.28 (9.46-26.34)	792 (537-1104)	6 (4.07-8.36)	-3.82 (-4--3.65)
Ireland	45 (36-56)	4.59 (3.65-5.74)	11 (8-15)	1.2 (0.87-1.6)	-5.68 (-6.42--4.92)
Israel	39 (31-49)	2.86 (2.25-3.62)	16 (12-20)	0.72 (0.53-0.94)	-6.27 (-6.91--5.63)
Italy	422 (374-485)	3.37 (2.98-3.88)	72 (49-104)	0.83 (0.57-1.19)	-5.88 (-6.39--5.38)
Jamaica	104 (79-140)	13.65 (10.36-18.31)	74 (50-109)	10.04 (6.73-14.69)	-2.58 (-3.41--1.75)
Japan	1869 (1460-2470)	6.65 (5.19-8.79)	735 (487-1087)	4.15 (2.75-6.14)	-1.85 (-1.98--1.72)

Jordan	175 (119-248)	12.83 (8.72-18.15)	176 (120-249)	4.96 (3.4-7.02)	-3.75 (-4.06--3.43)
Kazakhstan	745 (584-940)	17.05 (13.36-21.52)	485 (369-693)	12.42 (9.45-17.73)	-2.2 (-2.84--1.55)
Kenya	3544 (2413-5587)	44.72 (30.45-70.5)	6676 (4508-9490)	38.94 (26.3-55.35)	-0.1 (-0.39-0.2)
Kiribati	47 (26-74)	209.91 (115.64-332.33)	42 (20-70)	121.9 (57.99-201.99)	-1.97 (-2.07--1.87)
Kuwait	34 (25-44)	7.13 (5.36-9.38)	27 (17-42)	3.37 (2.07-5.26)	-2.51 (-2.78--2.23)
Kyrgyzstan	306 (236-385)	23.68 (18.23-29.79)	146 (111-193)	8.62 (6.56-11.34)	-4.16 (-4.54--3.78)
Lao People's Democratic Republic	1726 (1041-2469)	133.26 (80.37-190.7)	965 (629-1381)	45.83 (29.88-65.58)	-3.71 (-3.79--3.63)
Latvia	55 (42-72)	10.24 (7.72-13.34)	16 (10-23)	5.7 (3.74-8.35)	-1.93 (-2.24--1.63)
Lebanon	151 (105-220)	16.13 (11.14-23.46)	89 (55-135)	8.09 (4.97-12.22)	-2.06 (-2.33--1.8)
Lesotho	166 (92-268)	27.85 (15.42-44.82)	238 (147-351)	36.55 (22.54-53.78)	2.34 (1.68-3.01)
Liberia	386 (257-576)	66.7 (44.45-99.56)	784 (419-1331)	49.36 (26.43-83.82)	-1.26 (-1.5--1.02)
Libya	275 (185-430)	19.26 (12.91-30.05)	158 (104-236)	9.11 (6.03-13.61)	-2.64 (-2.95--2.32)
Lithuania	73 (50-104)	8.95 (6.14-12.73)	37 (25-53)	8.53 (5.8-12.27)	-0.08 (-0.45-0.29)
Luxembourg	4 (3-5)	4.99 (3.91-6.34)	1 (1-2)	1.36 (0.98-1.83)	-5.09 (-5.58--4.59)
Madagascar	2884 (1770-4320)	75.32 (46.22-112.81)	3702 (2376-5392)	42.03 (26.97-61.22)	-1.7 (-2.12--1.28)
Malawi	1810 (1098-2756)	59.29 (35.96-90.31)	2517 (1508-4030)	37.88 (22.7-60.64)	-1.83 (-2.13--1.53)

Malaysia	454 (327-628)	8.44 (6.08-11.67)	508 (348-744)	6.22 (4.26-9.12)	-2.12 (-2.5--1.73)
Maldives	8 (5-12)	11.23 (7.1-17.27)	3 (2-5)	3.18 (2.01-4.86)	-4.08 (-4.61--3.55)
Mali	2243 (1381-3352)	88.17 (54.27-131.73)	4494 (2503-7455)	61.59 (34.31-102.16)	-1.99 (-2.47--1.51)
Malta	3 (2-4)	3.46 (2.68-4.4)	1 (1-1)	1.38 (1-1.85)	-3.62 (-3.78--3.45)
Marshall Islands	21 (13-31)	137.34 (82.35-205.2)	11 (7-17)	68.3 (41.67-104.78)	-2.28 (-2.46--2.1)
Mauritania	459 (328-641)	72.14 (51.5-100.72)	377 (231-588)	27.98 (17.15-43.62)	-2.95 (-3.07--2.83)
Mauritius	73 (59-89)	22.42 (18.05-27.4)	23 (16-33)	8.68 (6.05-12.25)	-3.47 (-3.88--3.07)
Mexico	5653 (5219-6041)	19.42 (17.93-20.76)	2314 (1949-2828)	7.09 (5.97-8.67)	-3.45 (-3.96--2.93)
Micronesia (Federated States of)	41 (27-59)	119.9 (79.99-172.83)	20 (11-31)	62.01 (34.47-98.03)	-2.49 (-2.67--2.32)
Monaco	0 (0-0)	2.29 (1.67-3.17)	0 (0-0)	1.16 (0.82-1.61)	-2.2 (-2.58--1.81)
Mongolia	385 (263-536)	54.48 (37.27-75.86)	196 (127-295)	26.81 (17.38-40.37)	-3.03 (-3.52--2.55)
Montenegro	12 (9-15)	7.32 (5.44-9.67)	6 (4-8)	4.61 (3.3-6.3)	-1.7 (-2.28--1.11)
Morocco	2476 (1588-3572)	30.54 (19.58-44.06)	1208 (810-1705)	12.98 (8.71-18.33)	-3.37 (-3.57--3.17)
Mozambique	1731 (1018-2733)	42.44 (24.95-67)	4549 (2579-7278)	46.06 (26.11-73.69)	0.87 (0.6-1.14)
Myanmar	14605 (8809-24761)	113.88 (68.69-193.07)	3908 (2349-6558)	26.73 (16.07-44.86)	-5.49 (-5.73--5.25)

Namibia	107 (62-156)	22.58 (13.08-32.82)	112 (69-173)	15.38 (9.47-23.75)	-1.08 (-1.47--0.69)
Nauru	3 (2-4)	90.42 (53.4-147.66)	2 (1-3)	60.81 (31.48-97.55)	-1.41 (-1.87--0.94)
Nepal	645 (428-961)	10.85 (7.19-16.15)	332 (190-547)	3.48 (1.99-5.72)	-5.11 (-5.74--4.48)
Netherlands	120 (97-146)	3.66 (2.95-4.45)	26 (18-36)	0.84 (0.59-1.2)	-5.73 (-6.1--5.35)
New Zealand	28 (23-34)	3.33 (2.73-4.09)	9 (6-12)	0.99 (0.73-1.34)	-4.03 (-4.63--3.43)
Nicaragua	202 (153-275)	15.41 (11.68-20.97)	117 (74-183)	6.13 (3.92-9.64)	-3.44 (-3.83--3.04)
Niger	2292 (1439-3378)	93.74 (58.84-138.14)	5536 (3144-8855)	71.81 (40.78-114.85)	-1.87 (-2.29--1.45)
Nigeria	19584 (12739-32370)	69.56 (45.25-114.97)	35225 (20066-61833)	48.53 (27.64-85.18)	-1.12 (-1.27--0.97)
Niue	0 (0-1)	53.83 (33.5-83.92)	0 (0-0)	31.07 (18.93-50.48)	-2.11 (-2.22--2)
North Macedonia	28 (20-42)	5.46 (3.91-8)	12 (8-17)	3.13 (2.06-4.54)	-2.42 (-2.67--2.18)
Northern Mariana Islands	4 (2-5)	28.26 (17.64-41.54)	2 (1-2)	15.93 (11.37-21.5)	-2.11 (-2.44--1.79)
Norway	30 (23-40)	3.28 (2.54-4.38)	20 (12-31)	2.01 (1.23-3.11)	-2.73 (-3.13--2.34)
Oman	53 (33-80)	9.75 (6.08-14.53)	48 (30-73)	5.11 (3.14-7.66)	-1.45 (-1.79--1.12)
Pakistan	10605 (7492-14783)	29.71 (20.99-41.41)	14512 (9597-21098)	19.77 (13.08-28.75)	-1.73 (-1.96--1.51)
Palau	2 (1-3)	37.17 (21.89-59.36)	1 (1-1)	28.09 (17.7-41.51)	-0.81 (-0.9--0.72)

Palestine	90 (45-167)	13.26 (6.62-24.63)	110 (78-156)	6.84 (4.85-9.68)	-2.36 (-2.8--1.91)
Panama	64 (49-81)	8.49 (6.54-10.78)	31 (21-44)	2.87 (1.94-4.08)	-3.61 (-4.01--3.21)
Papua New Guinea	1036 (723-1484)	79.21 (55.25-113.43)	1660 (1066-2602)	56.9 (36.52-89.16)	-1.21 (-1.28--1.13)
Paraguay	160 (122-212)	12.96 (9.87-17.22)	125 (86-179)	6.4 (4.4-9.19)	-2.9 (-3.13--2.67)
Peru	2819 (2178-3653)	40.07 (30.95-51.92)	895 (579-1332)	10.31 (6.67-15.35)	-4.86 (-5.26--4.45)
Philippines	12105 (10159-16378)	59 (49.52-79.83)	10448 (8368-14928)	32.59 (26.1-46.57)	-1.4 (-1.77--1.02)
Poland	803 (694-945)	9.34 (8.08-11)	300 (239-382)	5.1 (4.07-6.5)	-1.32 (-1.69--0.94)
Portugal	159 (124-207)	6.36 (4.96-8.29)	17 (12-24)	1.04 (0.74-1.42)	-7.55 (-8.1--6.99)
Puerto Rico	70 (56-88)	7.12 (5.66-8.91)	14 (9-20)	2.03 (1.36-2.94)	-5.63 (-6.15--5.11)
Qatar	6 (4-9)	6.06 (3.69-9.12)	21 (11-34)	4.09 (2.23-6.58)	-0.98 (-1.24--0.71)
Republic of Korea	1850 (1466-2431)	14.06 (11.14-18.48)	300 (175-487)	3.58 (2.08-5.81)	-4.29 (-5.04--3.54)
Republic of Moldova	236 (185-297)	22.9 (17.95-28.86)	76 (55-102)	12.4 (8.99-16.66)	-1.98 (-2.21--1.74)
Romania	846 (682-1031)	14.31 (11.54-17.45)	162 (115-228)	5.22 (3.7-7.34)	-3.67 (-3.93--3.42)
Russian Federation	4749 (4135-5580)	15.08 (13.13-17.71)	2046 (1627-2619)	8.97 (7.14-11.49)	-2.38 (-3.03--1.73)
Rwanda	2661 (1739-4127)	117.74 (76.94-182.6)	1885 (1125-3087)	44.95 (26.83-73.6)	-4.23 (-4.57--3.88)

Saint Kitts and Nevis	1 (1-2)	9.69 (7.44-12.44)	1 (0-1)	4.54 (3.13-6.35)	-1.53 (-2.22--0.83)
Saint Lucia	6 (4-7)	12.59 (9.81-15.81)	3 (2-4)	6.87 (4.94-9.45)	-2.46 (-2.74--2.17)
Saint Vincent and the Grenadines	7 (5-9)	18.79 (14.17-24.23)	3 (2-4)	10.69 (7.68-14.37)	-2.58 (-3.01--2.15)
Samoa	32 (19-50)	53.95 (32.72-85.36)	21 (13-31)	30.69 (19.39-44.6)	-1.95 (-2.02--1.87)
San Marino	0 (0-0)	1.25 (0.88-1.74)	0 (0-0)	0.93 (0.61-1.38)	-1.19 (-1.46--0.92)
Sao Tome and Principe	15 (9-25)	38.23 (21.52-61.43)	16 (10-26)	23.02 (15.01-39.08)	-2.51 (-3.28--1.72)
Saudi Arabia	522 (318-832)	10.26 (6.25-16.36)	394 (236-583)	4.76 (2.85-7.05)	-2.25 (-2.47--2.03)
Senegal	1756 (1053-2608)	72.61 (43.52-107.8)	2360 (1308-3679)	47.19 (26.15-73.57)	-1.27 (-1.5--1.03)
Serbia	131 (98-170)	6.27 (4.68-8.11)	42 (29-61)	2.67 (1.82-3.91)	-3.44 (-3.65--3.22)
Seychelles	6 (4-8)	25.66 (17.32-34.84)	3 (2-4)	14.15 (9.15-20.88)	-1.95 (-2.44--1.46)
Sierra Leone	681 (419-1043)	63.6 (39.13-97.42)	1647 (964-2499)	59.88 (35.06-90.87)	0.35 (0.16-0.53)
Singapore	56 (41-77)	6.8 (4.97-9.39)	25 (15-41)	3.39 (1.99-5.5)	-2.78 (-3.06--2.5)
Slovakia	94 (73-120)	7.39 (5.71-9.46)	39 (27-56)	4.69 (3.24-6.6)	-1.58 (-1.72--1.45)
Slovenia	28 (21-36)	6.25 (4.76-8.05)	9 (6-12)	2.93 (2.07-4.06)	-3.77 (-4.33--3.21)
Solomon Islands	117 (68-184)	100.87 (59.24-159.28)	137 (94-199)	67.36 (46.15-98.33)	-1.28 (-1.36--1.21)

Somalia	1311 (620-2263)	57.21 (27.08-98.77)	3991 (2052-7012)	58.27 (29.96-102.37)	-0.54 (-1.04--0.05)
South Africa	1672 (1194-2180)	14.02 (10.01-18.28)	1098 (786-1442)	7.83 (5.6-10.28)	-1.35 (-2.35--0.34)
South Sudan	764 (378-1486)	38.21 (18.89-74.3)	943 (544-1691)	29.17 (16.82-52.3)	-1.31 (-1.55--1.07)
Spain	592 (465-744)	6.08 (4.77-7.64)	61 (43-87)	0.88 (0.62-1.25)	-6.82 (-7.26--6.39)
Sri Lanka	545 (299-745)	10.52 (5.76-14.37)	157 (105-227)	3.04 (2.03-4.41)	-3.95 (-4.41--3.49)
Sudan	2094 (1114-3369)	32.45 (17.26-52.21)	2373 (1343-3957)	17.64 (9.99-29.42)	-1.9 (-1.99--1.82)
Suriname	41 (24-55)	33.97 (19.91-45.75)	21 (15-29)	14.79 (10.43-20.18)	-3.43 (-3.82--3.05)
Sweden	77 (63-93)	4.67 (3.81-5.64)	35 (27-46)	2.04 (1.55-2.66)	-2.6 (-2.77--2.42)
Switzerland	31 (23-41)	2.32 (1.74-3.06)	12 (8-17)	0.86 (0.58-1.22)	-3.88 (-4.11--3.65)
Syrian Arab Republic	736 (500-1147)	16.88 (11.47-26.31)	320 (210-471)	6.56 (4.3-9.66)	-3.55 (-3.95--3.15)
Taiwan (Province of China)	586 (474-717)	10.25 (8.29-12.53)	89 (61-125)	2.35 (1.63-3.32)	-6.05 (-6.52--5.59)
Tajikistan	503 (381-708)	30.33 (23-42.68)	525 (347-863)	19.45 (12.87-31.97)	-3.45 (-4.2--2.7)
Thailand	1863 (1093-2757)	10.42 (6.11-15.41)	567 (368-819)	4.32 (2.81-6.24)	-4.51 (-5.37--3.64)
Timor-Leste	169 (86-253)	73.42 (37.48-109.93)	155 (93-221)	34.04 (20.35-48.37)	-3.41 (-4.03--2.79)
Togo	724 (498-1015)	60.72 (41.72-85.1)	1131 (694-1620)	45.8 (28.11-65.63)	-0.6 (-0.75--0.44)

Tokelau	0 (0-0)	58.88 (34.08-91.22)	0 (0-0)	25.61 (16.76-38.02)	-2.95 (-3.02--2.88)
Tonga	14 (9-20)	41.44 (26.4-58.44)	10 (7-14)	31.95 (21.42-46.27)	-0.79 (-1.02--0.56)
Trinidad and Tobago	53 (42-66)	15.57 (12.31-19.27)	22 (14-32)	7.94 (5.07-11.58)	-2.28 (-2.86--1.7)
Tunisia	434 (249-746)	16.23 (9.32-27.91)	195 (124-320)	7.87 (4.99-12.9)	-2.46 (-2.58--2.34)
Turkmenistan	281 (223-353)	24.46 (19.42-30.73)	307 (218-426)	24.51 (17.41-33.94)	-0.66 (-1.22--0.1)
Tuvalu	2 (1-4)	100.67 (55.24-163.62)	1 (1-2)	43.11 (26.45-63.71)	-2.69 (-2.83--2.55)
Türkiye	3179 (2199-4470)	16.73 (11.58-23.53)	1357 (895-2003)	7.13 (4.7-10.53)	-2.92 (-3.1--2.74)
Uganda	2357 (1413-3711)	41.69 (25-65.64)	5358 (3216-8367)	37.21 (22.34-58.1)	-1.26 (-1.73--0.78)
Ukraine	1622 (1303-1996)	14.97 (12.02-18.42)	1197 (870-1664)	18.14 (13.18-25.21)	0.06 (-0.63-0.75)
United Arab Emirates	18 (11-26)	4.23 (2.66-6.25)	30 (17-48)	2.87 (1.65-4.52)	-1.28 (-1.44--1.12)
United Kingdom	647 (596-723)	5.46 (5.04-6.11)	201 (165-295)	1.7 (1.4-2.51)	-4.73 (-5.02--4.43)
United Republic of Tanzania	2546 (1723-3648)	29.89 (20.23-42.83)	4091 (2481-6372)	22.3 (13.53-34.73)	-0.95 (-1.18--0.72)
United States of America	2175 (1757-2738)	3.95 (3.19-4.97)	1391 (1004-1966)	2.15 (1.55-3.04)	-2.3 (-2.79--1.81)
United States Virgin Islands	3 (2-4)	9.88 (6.71-14.04)	1 (0-1)	3.37 (2.28-4.92)	-5.36 (-6.26--4.46)
Uruguay	43 (35-53)	5.65 (4.51-6.87)	20 (15-27)	2.75 (2.05-3.57)	-2.89 (-3.29--2.48)

Uzbekistan	1479 (1189-1830)	23.15 (18.61-28.64)	1672 (1260-2248)	18.68 (14.08-25.11)	-1.81 (-2.48--1.14)
Vanuatu	47 (28-70)	100.06 (60.23-148.19)	68 (44-101)	76.27 (49.12-112.1)	-1.51 (-1.74--1.28)
Venezuela (Bolivarian Republic of)	697 (535-907)	11.63 (8.92-15.13)	442 (293-629)	6.59 (4.37-9.38)	-2.38 (-3--1.76)
Viet Nam	640 (429-913)	2.97 (1.99-4.24)	419 (247-675)	1.99 (1.17-3.2)	-1.33 (-1.61--1.05)
Yemen	1317 (691-2220)	31.18 (16.37-52.57)	2090 (1261-3183)	20.57 (12.4-31.32)	-1.65 (-1.86--1.44)
Zambia	1822 (1200-2677)	65.71 (43.26-96.54)	2168 (1311-3228)	35.21 (21.29-52.44)	-2.58 (-3.01--2.16)
Zimbabwe	1354 (844-2216)	37.72 (23.52-61.74)	2572 (1459-4170)	52.52 (29.78-85.14)	1.53 (1.25-1.81)

Supplementary Table 4：Global Cases and Age-Standardized Deaths of Gastrointestinal Ulcers Among Individuals Aged 10–24 Years in 2019

	Number1990	ASR 1990	Number 2019	ASR 2019	EAPC CI
Global	8063 (6836-9399)	0.52 (0.44-0.61)	4404 (3826-5042)	0.24 (0.21-0.27)	-3.1 (-3.28--2.92)
Sex					
Female	3963 (2982-5004)	0.52 (0.39-0.66)	2096 (1762-2502)	0.23 (0.19-0.28)	-3.3 (-3.56--3.04)
Male	4100 (3557-4787)	0.52 (0.45-0.61)	2307 (1972-2729)	0.24 (0.21-0.29)	-2.91 (-3.02--2.79)
SDI regions					
High SDI	112 (105-121)	0.06 (0.06-0.07)	24 (21-26)	0.01 (0.01-0.02)	-5.42 (-5.65--5.19)
High-middle SDI	532 (473-605)	0.18 (0.16-0.2)	154 (135-178)	0.06 (0.05-0.07)	-4.09 (-4.29--3.88)
Middle SDI	1798 (1580-2184)	0.33 (0.29-0.4)	680 (584-830)	0.12 (0.11-0.15)	-3.44 (-3.56--3.32)
Low-middle SDI	3940 (3246-4699)	1.11 (0.92-1.33)	1809 (1535-2119)	0.35 (0.3-0.42)	-4.45 (-4.77--4.13)
Low SDI	1676 (1297-2052)	1.02 (0.79-1.25)	1734 (1420-2068)	0.47 (0.38-0.56)	-2.96 (-3.13--2.79)
GBD region					
Africa	1409 (1141-1720)	0.7 (0.57-0.86)	1890 (1482-2368)	0.45 (0.35-0.56)	-1.47 (-1.54--1.4)
African Region	1267 (1022-1572)	0.78 (0.63-0.97)	1754 (1366-2218)	0.49 (0.38-0.62)	-1.55 (-1.64--1.47)
America	433 (406-467)	0.22 (0.21-0.24)	197 (169-231)	0.09 (0.07-0.1)	-3.33 (-3.57--3.08)
Andean Latin America	71 (59-86)	0.57 (0.48-0.7)	25 (19-33)	0.15 (0.11-0.19)	-4.91 (-5.14--4.68)
Asia	6014 (4981-7182)	0.62 (0.51-0.74)	2249 (1882-2744)	0.21 (0.18-0.26)	-4.28 (-4.6--3.96)
Australasia	2 (1-2)	0.03 (0.03-0.04)	0 (0-0)	0 (0-0.01)	-7.16 (-7.72--6.59)

Caribbean	41 (30-55)	0.38 (0.28-0.52)	26 (17-35)	0.22 (0.15-0.31)	-2.08 (-2.44--1.71)
Central Asia	61 (54-68)	0.31 (0.27-0.34)	48 (40-59)	0.21 (0.18-0.26)	-2.56 (-3.06--2.05)
Central Europe	33 (30-36)	0.11 (0.1-0.13)	7 (6-9)	0.04 (0.03-0.05)	-3.65 (-3.87--3.43)
Central Latin America	191 (177-207)	0.35 (0.33-0.38)	92 (74-114)	0.14 (0.11-0.17)	-3.36 (-3.59--3.13)
Central Sub-Saharan Africa	156 (109-214)	0.9 (0.63-1.23)	205 (133-299)	0.48 (0.31-0.7)	-1.94 (-2.07--1.81)
Commonwealth High Income	14 (13-15)	0.06 (0.05-0.06)	4 (3-5)	0.01 (0.01-0.02)	-5.39 (-5.66--5.12)
Commonwealth Low Income	458 (341-591)	0.68 (0.5-0.87)	444 (326-582)	0.37 (0.27-0.49)	-2.02 (-2.3--1.74)
Commonwealth Middle Income	4201 (3412-5085)	1.17 (0.95-1.41)	2102 (1724-2534)	0.35 (0.29-0.42)	-4.71 (-5.14--4.28)
East Asia	1049 (863-1291)	0.28 (0.23-0.35)	133 (111-161)	0.06 (0.05-0.07)	-5.75 (-5.9--5.6)
East Asia & Pacific - WB	1716 (1463-2206)	0.3 (0.26-0.39)	443 (375-602)	0.1 (0.09-0.14)	-3.98 (-4.17--3.79)
Eastern Europe	78 (71-88)	0.17 (0.15-0.19)	36 (30-43)	0.11 (0.09-0.13)	-2.16 (-2.69--1.62)
Eastern Mediterranean Region	421 (331-517)	0.35 (0.27-0.43)	427 (338-537)	0.2 (0.16-0.25)	-1.95 (-2.03--1.87)
Eastern Sub-Saharan Africa	553 (399-742)	0.89 (0.65-1.2)	655 (504-827)	0.47 (0.36-0.59)	-2.44 (-2.58--2.3)
Europe	202 (185-226)	0.11 (0.1-0.13)	64 (57-74)	0.05 (0.04-0.05)	-3.81 (-4.15--3.46)
Europe & Central Asia - WB	245 (225-272)	0.13 (0.12-0.14)	104 (93-119)	0.07 (0.06-0.07)	-3.02 (-3.4--2.64)
European Region	246 (226-273)	0.13 (0.12-0.14)	105 (94-119)	0.06 (0.06-0.07)	-3.05 (-3.42--2.67)
High-income Asia Pacific	35 (31-43)	0.08 (0.07-0.1)	3 (3-3)	0.01 (0.01-0.01)	-6.93 (-7.32--6.55)
High-income North America	22 (20-23)	0.04 (0.03-0.04)	7 (6-8)	0.01 (0.01-0.01)	-4.58 (-4.98--4.18)
Latin America & Caribbean - WB	412 (385-445)	0.3 (0.28-0.32)	190 (162-224)	0.12 (0.1-0.14)	-3.24 (-3.49--2.99)
Middle East & North Africa - WB	250 (199-308)	0.3 (0.24-0.37)	130 (100-173)	0.11 (0.08-0.15)	-3.28 (-3.36--3.19)

North Africa and Middle East	353 (272-441)	0.32 (0.25-0.4)	234 (179-303)	0.14 (0.11-0.19)	-2.61 (-2.69--2.54)
North America	22 (20-23)	0.04 (0.03-0.04)	7 (6-8)	0.01 (0.01-0.01)	-4.58 (-4.98--4.18)
Oceania	21 (16-27)	1 (0.76-1.32)	28 (19-42)	0.72 (0.48-1.08)	-1.18 (-1.26--1.11)
Region of the Americas	433 (406-467)	0.22 (0.21-0.24)	197 (169-231)	0.09 (0.07-0.1)	-3.33 (-3.57--3.08)
South-East Asia Region	4332 (3444-5303)	1.08 (0.86-1.32)	1574 (1228-1952)	0.28 (0.22-0.35)	-5.28 (-5.76--4.8)
South Asia	4083 (3277-4980)	1.22 (0.98-1.49)	1631 (1287-2011)	0.31 (0.24-0.38)	-5.35 (-5.82--4.88)
South Asia - WB	4136 (3318-5039)	1.2 (0.96-1.46)	1701 (1349-2081)	0.31 (0.25-0.38)	-5.26 (-5.72--4.8)
Southeast Asia	614 (471-908)	0.41 (0.32-0.61)	280 (222-421)	0.16 (0.13-0.25)	-3.25 (-3.35--3.15)
Southern Latin America	10 (8-12)	0.07 (0.06-0.09)	6 (4-7)	0.04 (0.03-0.05)	-2.24 (-2.63--1.85)
Southern Sub-Saharan Africa	44 (30-61)	0.25 (0.17-0.35)	53 (37-79)	0.25 (0.17-0.37)	0.37 (-0.19-0.94)
Sub-Saharan Africa - WB	1278 (1021-1586)	0.79 (0.63-0.98)	1825 (1428-2290)	0.5 (0.39-0.62)	-1.56 (-1.66--1.46)
Tropical Latin America	101 (93-109)	0.21 (0.19-0.23)	42 (38-48)	0.08 (0.07-0.09)	-2.87 (-3.18--2.56)
Western Europe	46 (43-50)	0.06 (0.05-0.06)	8 (7-10)	0.01 (0.01-0.01)	-6.14 (-6.42--5.86)
Western Pacific Region	1357 (1151-1657)	0.29 (0.25-0.35)	342 (292-445)	0.1 (0.09-0.13)	-3.72 (-3.97--3.47)
Western Sub-Saharan Africa	499 (372-694)	0.84 (0.63-1.17)	885 (617-1264)	0.59 (0.41-0.84)	-1.08 (-1.21--0.96)
World Bank High Income	141 (133-151)	0.06 (0.06-0.07)	30 (27-33)	0.01 (0.01-0.02)	-5.23 (-5.46--4.99)
World Bank Low Income	869 (638-1104)	0.84 (0.62-1.07)	1168 (913-1455)	0.5 (0.39-0.63)	-1.85 (-1.92--1.77)
World Bank Lower Middle Income	5418 (4446-6431)	0.92 (0.75-1.09)	2843 (2389-3349)	0.31 (0.26-0.37)	-4.17 (-4.47--3.87)
World Bank Upper Middle Income	1631 (1427-1914)	0.26 (0.23-0.3)	359 (319-410)	0.07 (0.06-0.08)	-4.62 (-4.75--4.5)
Countries					

Afghanistan	46 (25-70)	1.2 (0.66-1.84)	69 (41-107)	0.53 (0.32-0.82)	-3.01 (-3.32--2.71)
Albania	1 (1-1)	0.1 (0.07-0.14)	0 (0-0)	0.03 (0.02-0.05)	-4.9 (-5.72--4.08)
Algeria	33 (21-49)	0.39 (0.24-0.58)	10 (7-15)	0.1 (0.07-0.16)	-4.39 (-4.52--4.27)
American Samoa	0 (0-0)	0.54 (0.37-0.78)	0 (0-0)	0.27 (0.18-0.39)	-2.87 (-3.12--2.63)
Andorra	0 (0-0)	0.01 (0.01-0.02)	0 (0-0)	0 (0-0.01)	-3.92 (-4.11--3.73)
Angola	31 (17-49)	0.97 (0.52-1.53)	41 (24-64)	0.42 (0.25-0.67)	-2.55 (-2.76--2.34)
Antigua and Barbuda	0 (0-0)	0.11 (0.08-0.14)	0 (0-0)	0.05 (0.04-0.07)	-4.1 (-4.74--3.46)
Argentina	6 (5-8)	0.07 (0.06-0.09)	4 (3-6)	0.04 (0.03-0.06)	-1.59 (-1.99--1.18)
Armenia	1 (1-2)	0.13 (0.1-0.18)	1 (0-1)	0.09 (0.07-0.13)	-2.14 (-3.46--0.8)
Australia	1 (1-2)	0.03 (0.02-0.04)	0 (0-0)	0 (0-0.01)	-7.41 (-8.01--6.8)
Austria	1 (1-1)	0.05 (0.04-0.06)	0 (0-0)	0.01 (0-0.01)	-7.87 (-8.29--7.44)
Azerbaijan	7 (6-10)	0.35 (0.26-0.46)	4 (3-6)	0.17 (0.11-0.26)	-4.71 (-5.6--3.81)
Bahamas	0 (0-0)	0.2 (0.15-0.27)	0 (0-0)	0.12 (0.08-0.17)	-1.56 (-1.91--1.21)
Bahrain	0 (0-0)	0.18 (0.13-0.25)	0 (0-0)	0.07 (0.04-0.11)	-4.01 (-4.49--3.52)
Bangladesh	254 (162-377)	0.72 (0.46-1.07)	94 (39-152)	0.21 (0.09-0.34)	-5.09 (-6.19--3.98)
Barbados	0 (0-0)	0.21 (0.15-0.27)	0 (0-0)	0.09 (0.06-0.13)	-3.99 (-4.36--3.62)
Belarus	2 (1-3)	0.09 (0.07-0.12)	0 (0-1)	0.03 (0.02-0.05)	-3.8 (-4.21--3.38)
Belgium	1 (1-2)	0.07 (0.05-0.08)	0 (0-0)	0.01 (0.01-0.02)	-6.33 (-6.66--6)
Belize	0 (0-0)	0.13 (0.09-0.18)	0 (0-0)	0.09 (0.06-0.13)	-2.68 (-3.3--2.06)
Benin	12 (7-19)	0.83 (0.51-1.31)	26 (15-41)	0.63 (0.36-1.01)	-0.82 (-0.95--0.7)

Bermuda	0 (0-0)	0.17 (0.13-0.23)	0 (0-0)	0.04 (0.03-0.06)	-5.38 (-5.8--4.95)
Bhutan	2 (1-3)	0.99 (0.46-1.61)	1 (0-1)	0.3 (0.14-0.56)	-4.62 (-4.88--4.35)
Bolivia (Plurinational State of)	14 (8-21)	0.7 (0.41-1.05)	6 (4-9)	0.19 (0.12-0.28)	-4.81 (-5.01--4.61)
Bosnia and Herzegovina	1 (1-2)	0.1 (0.07-0.14)	0 (0-0)	0.04 (0.02-0.06)	-4.59 (-5.41--3.77)
Botswana	1 (1-2)	0.32 (0.17-0.52)	1 (1-2)	0.22 (0.12-0.36)	-1.56 (-2.02--1.1)
Brazil	99 (91-107)	0.21 (0.19-0.23)	41 (37-46)	0.08 (0.07-0.09)	-2.86 (-3.17--2.54)
Brunei Darussalam	0 (0-0)	0.26 (0.16-0.38)	0 (0-0)	0.03 (0.02-0.05)	-8.05 (-8.75--7.35)
Bulgaria	3 (2-3)	0.14 (0.11-0.18)	1 (0-1)	0.06 (0.04-0.09)	-3.14 (-3.46--2.81)
Burkina Faso	13 (7-20)	0.43 (0.25-0.66)	31 (15-54)	0.43 (0.21-0.75)	0.3 (0.14-0.47)
Burundi	23 (13-38)	1.37 (0.79-2.29)	32 (19-51)	0.83 (0.5-1.34)	-1.65 (-1.85--1.45)
Cabo Verde	1 (1-1)	0.83 (0.56-1.18)	0 (0-0)	0.17 (0.11-0.24)	-5.39 (-5.84--4.93)
Cambodia	78 (48-113)	2.43 (1.51-3.52)	32 (20-54)	0.71 (0.43-1.18)	-4.49 (-4.66--4.33)
Cameroon	24 (17-35)	0.76 (0.52-1.07)	51 (26-81)	0.53 (0.27-0.84)	-1.19 (-1.46--0.91)
Canada	2 (2-3)	0.04 (0.03-0.04)	1 (0-1)	0.01 (0.01-0.01)	-4.78 (-5.33--4.22)
Central African Republic	14 (9-21)	1.68 (1.07-2.48)	20 (11-33)	1.15 (0.62-1.88)	-1.44 (-1.54--1.34)
Chad	20 (13-29)	1.08 (0.69-1.6)	49 (31-71)	0.92 (0.57-1.33)	-0.34 (-0.68-0)
Chile	3 (2-4)	0.08 (0.06-0.1)	1 (1-1)	0.02 (0.02-0.03)	-4.32 (-4.6--4.04)
China	1018 (831-1263)	0.28 (0.23-0.35)	119 (98-149)	0.05 (0.04-0.07)	-6.02 (-6.16--5.87)
Colombia	32 (26-38)	0.31 (0.25-0.37)	7 (5-11)	0.06 (0.04-0.09)	-5.58 (-6.07--5.09)
Comoros	1 (0-2)	0.71 (0.08-1.38)	1 (0-2)	0.47 (0.19-0.83)	-2.58 (-3.67--1.47)

Congo	7 (4-12)	0.89 (0.52-1.44)	5 (3-8)	0.32 (0.17-0.54)	-3.6 (-3.88--3.31)
Cook Islands	0 (0-0)	0.16 (0.1-0.25)	0 (0-0)	0.05 (0.03-0.08)	-4.52 (-4.69--4.35)
Costa Rica	1 (1-1)	0.08 (0.06-0.1)	1 (0-1)	0.05 (0.04-0.08)	-2.07 (-2.54--1.59)
Croatia	1 (1-1)	0.07 (0.05-0.09)	0 (0-0)	0.02 (0.01-0.03)	-5.08 (-5.44--4.71)
Cuba	4 (3-5)	0.13 (0.1-0.17)	1 (0-1)	0.03 (0.02-0.05)	-5.58 (-6.23--4.93)
Cyprus	0 (0-0)	0.02 (0.02-0.03)	0 (0-0)	0.01 (0-0.01)	-4.48 (-4.9--4.05)
Czechia	1 (1-2)	0.05 (0.04-0.07)	0 (0-0)	0.02 (0.01-0.03)	-3.62 (-3.92--3.31)
C<U+00F4>te d'Ivoire	33 (20-49)	0.86 (0.53-1.27)	51 (29-80)	0.63 (0.36-0.99)	-1.21 (-1.61--0.81)
Democratic People's Republic of Korea	23 (12-39)	0.42 (0.22-0.7)	13 (8-21)	0.24 (0.14-0.38)	-1.63 (-1.69--1.57)
Democratic Republic of the Congo	100 (64-145)	0.83 (0.53-1.21)	136 (83-216)	0.47 (0.29-0.74)	-1.63 (-1.79--1.47)
Denmark	1 (0-1)	0.05 (0.04-0.07)	0 (0-0)	0.02 (0.01-0.02)	-4.95 (-5.64--4.26)
Djibouti	1 (0-2)	0.59 (0.29-1.05)	1 (1-2)	0.34 (0.15-0.58)	-1.84 (-2.27--1.41)
Dominica	0 (0-0)	0.18 (0.13-0.25)	0 (0-0)	0.11 (0.07-0.15)	-1.76 (-2.16--1.36)
Dominican Republic	12 (9-16)	0.5 (0.38-0.66)	8 (4-13)	0.28 (0.15-0.44)	-2.19 (-2.92--1.45)
Ecuador	18 (15-21)	0.54 (0.45-0.65)	7 (5-10)	0.14 (0.1-0.2)	-5 (-5.32--4.68)
Egypt	57 (40-76)	0.33 (0.23-0.44)	37 (17-68)	0.13 (0.06-0.24)	-2.3 (-2.77--1.82)
El Salvador	11 (9-14)	0.63 (0.51-0.77)	3 (2-4)	0.14 (0.09-0.22)	-5.76 (-6.4--5.12)
Equatorial Guinea	2 (1-3)	1.27 (0.73-1.98)	1 (0-2)	0.22 (0.09-0.41)	-6.67 (-7.17--6.17)
Eritrea	9 (5-15)	0.94 (0.51-1.54)	16 (9-26)	0.69 (0.38-1.15)	-1.14 (-1.35--0.92)
Estonia	0 (0-0)	0.08 (0.06-0.1)	0 (0-0)	0.02 (0.01-0.03)	-5.38 (-5.93--4.82)

Eswatini	1 (0-1)	0.26 (0.14-0.43)	1 (1-2)	0.31 (0.16-0.52)	1.47 (0.6-2.34)
Ethiopia	231 (144-334)	1.41 (0.88-2.04)	132 (85-196)	0.35 (0.23-0.52)	-5.2 (-5.43--4.97)
Fiji	2 (1-3)	0.76 (0.48-1.11)	1 (1-2)	0.49 (0.31-0.72)	-1.36 (-1.54--1.18)
Finland	1 (1-1)	0.07 (0.06-0.09)	0 (0-0)	0.02 (0.02-0.03)	-4.15 (-4.44--3.85)
France	6 (5-7)	0.04 (0.04-0.05)	1 (1-1)	0.01 (0-0.01)	-7.04 (-7.47--6.61)
Gabon	2 (1-3)	0.64 (0.37-1.01)	1 (1-2)	0.26 (0.13-0.46)	-3.1 (-3.23--2.98)
Gambia	3 (2-4)	0.85 (0.49-1.37)	4 (2-6)	0.53 (0.32-0.81)	-1.71 (-2.08--1.35)
Georgia	3 (3-5)	0.27 (0.2-0.35)	1 (1-1)	0.13 (0.09-0.18)	-2.39 (-3.24--1.53)
Germany	9 (7-11)	0.06 (0.05-0.07)	2 (2-3)	0.02 (0.01-0.02)	-4.77 (-5.06--4.48)
Ghana	18 (11-28)	0.39 (0.24-0.6)	24 (14-35)	0.24 (0.14-0.35)	-1.1 (-1.33--0.87)
Greece	1 (1-1)	0.04 (0.03-0.05)	0 (0-1)	0.03 (0.02-0.04)	-0.86 (-1.17--0.55)
Greenland	0 (0-0)	0.19 (0.11-0.28)	0 (0-0)	0.07 (0.04-0.12)	-4.22 (-4.85--3.59)
Grenada	0 (0-0)	0.23 (0.17-0.29)	0 (0-0)	0.11 (0.07-0.15)	-2.29 (-2.6--1.98)
Guam	0 (0-0)	0.25 (0.16-0.4)	0 (0-0)	0.1 (0.07-0.15)	-3.36 (-3.69--3.03)
Guatemala	35 (28-45)	1.42 (1.12-1.81)	33 (23-45)	0.58 (0.4-0.8)	-3.78 (-4.22--3.34)
Guinea	17 (10-25)	0.95 (0.59-1.42)	30 (17-45)	0.74 (0.42-1.12)	-0.37 (-0.52--0.23)
Guinea-Bissau	5 (3-8)	1.68 (1.03-2.53)	6 (4-10)	1.04 (0.68-1.58)	-1.22 (-1.45--0.98)
Guyana	2 (1-2)	0.62 (0.45-0.83)	1 (0-1)	0.34 (0.22-0.5)	-2 (-2.38--1.63)
Haiti	18 (8-30)	0.9 (0.42-1.54)	13 (7-21)	0.36 (0.19-0.57)	-3 (-3.28--2.72)
Honduras	20 (14-27)	1.28 (0.92-1.74)	9 (5-14)	0.29 (0.17-0.44)	-5.4 (-5.62--5.17)

Hungary	3 (2-3)	0.11 (0.09-0.14)	0 (0-1)	0.03 (0.02-0.04)	-4.59 (-4.78--4.4)
Iceland	0 (0-0)	0.04 (0.03-0.06)	0 (0-0)	0.01 (0-0.01)	-6.32 (-6.62--6.02)
India	3710 (2936-4641)	1.44 (1.14-1.8)	1377 (1047-1746)	0.35 (0.26-0.44)	-5.58 (-6.13--5.03)
Indonesia	109 (78-191)	0.18 (0.13-0.32)	28 (20-50)	0.04 (0.03-0.07)	-5.45 (-5.69--5.21)
Iran (Islamic Republic of)	69 (55-91)	0.36 (0.28-0.48)	22 (18-26)	0.12 (0.1-0.15)	-3.22 (-3.42--3.02)
Iraq	11 (6-20)	0.2 (0.11-0.35)	7 (4-10)	0.05 (0.03-0.08)	-4.88 (-5.14--4.63)
Ireland	1 (0-1)	0.06 (0.05-0.08)	0 (0-0)	0.01 (0.01-0.02)	-6.97 (-7.91--6.03)
Israel	1 (0-1)	0.04 (0.03-0.05)	0 (0-0)	0.01 (0.01-0.01)	-6.97 (-7.61--6.31)
Italy	5 (5-5)	0.04 (0.04-0.04)	0 (0-0)	0 (0-0)	-9.44 (-10--8.89)
Jamaica	1 (1-2)	0.18 (0.14-0.25)	1 (1-1)	0.13 (0.09-0.2)	-2.76 (-3.66--1.85)
Japan	14 (13-15)	0.05 (0.05-0.05)	2 (2-2)	0.01 (0.01-0.01)	-5.15 (-5.3--5)
Jordan	2 (1-3)	0.16 (0.11-0.23)	2 (1-2)	0.04 (0.03-0.07)	-5 (-5.43--4.56)
Kazakhstan	9 (7-12)	0.22 (0.17-0.28)	6 (4-9)	0.16 (0.11-0.23)	-2.39 (-3.11--1.67)
Kenya	48 (32-78)	0.61 (0.4-0.98)	90 (59-131)	0.52 (0.34-0.77)	-0.09 (-0.41-0.23)
Kiribati	1 (0-1)	2.75 (1.4-4.53)	1 (0-1)	1.58 (0.65-2.75)	-1.96 (-2.04--1.87)
Kuwait	0 (0-0)	0.07 (0.05-0.09)	0 (0-0)	0.02 (0.01-0.02)	-5.41 (-5.95--4.86)
Kyrgyzstan	4 (3-5)	0.32 (0.24-0.41)	2 (1-2)	0.1 (0.07-0.14)	-4.77 (-5.18--4.37)
Lao People's Democratic Republic	23 (13-33)	1.76 (1.01-2.57)	13 (8-19)	0.61 (0.39-0.89)	-3.61 (-3.67--3.54)
Latvia	1 (0-1)	0.1 (0.07-0.13)	0 (0-0)	0.04 (0.02-0.05)	-4.13 (-4.62--3.64)
Lebanon	2 (1-3)	0.2 (0.13-0.31)	1 (1-2)	0.09 (0.05-0.14)	-2.62 (-2.96--2.27)

Lesotho	2 (1-4)	0.35 (0.18-0.6)	3 (2-5)	0.48 (0.28-0.74)	2.59 (1.86-3.33)
Liberia	5 (3-7)	0.79 (0.48-1.24)	10 (5-17)	0.61 (0.3-1.09)	-1.07 (-1.32--0.82)
Libya	3 (2-6)	0.24 (0.16-0.39)	2 (1-3)	0.1 (0.06-0.16)	-3.23 (-3.59--2.86)
Lithuania	1 (0-1)	0.06 (0.05-0.08)	0 (0-0)	0.06 (0.04-0.08)	-0.44 (-1.11-0.24)
Luxembourg	0 (0-0)	0.06 (0.05-0.08)	0 (0-0)	0.01 (0.01-0.01)	-6.8 (-7.39--6.21)
Madagascar	40 (23-61)	1.03 (0.6-1.58)	49 (30-74)	0.56 (0.34-0.85)	-1.76 (-2.2--1.32)
Malawi	24 (14-38)	0.8 (0.46-1.24)	33 (19-55)	0.5 (0.28-0.83)	-1.93 (-2.24--1.61)
Malaysia	6 (4-8)	0.1 (0.07-0.15)	6 (4-9)	0.07 (0.05-0.11)	-2.62 (-3.07--2.18)
Maldives	0 (0-0)	0.14 (0.08-0.22)	0 (0-0)	0.02 (0.01-0.03)	-6.51 (-7.05--5.97)
Mali	28 (15-43)	1.1 (0.61-1.71)	55 (28-98)	0.76 (0.38-1.34)	-1.16 (-1.4--0.91)
Malta	0 (0-0)	0.05 (0.04-0.06)	0 (0-0)	0.02 (0.01-0.02)	-4.21 (-4.39--4.03)
Marshall Islands	0 (0-0)	1.87 (1.05-2.89)	0 (0-0)	0.91 (0.52-1.44)	-2.29 (-2.5--2.08)
Mauritania	6 (4-9)	0.94 (0.64-1.37)	4 (2-7)	0.32 (0.18-0.55)	-3.26 (-3.41--3.12)
Mauritius	1 (1-1)	0.31 (0.24-0.38)	0 (0-0)	0.11 (0.07-0.16)	-3.77 (-4.19--3.34)
Mexico	79 (72-84)	0.27 (0.25-0.29)	32 (27-39)	0.1 (0.08-0.12)	-3.47 (-4--2.94)
Micronesia (Federated States of)	1 (0-1)	1.57 (1-2.34)	0 (0-0)	0.82 (0.42-1.36)	-2.39 (-2.57--2.2)
Monaco	0 (0-0)	0.03 (0.02-0.04)	0 (0-0)	0.01 (0.01-0.02)	-3.07 (-3.54--2.6)
Mongolia	5 (4-7)	0.74 (0.5-1.05)	3 (2-4)	0.36 (0.22-0.57)	-3.15 (-3.65--2.65)
Montenegro	0 (0-0)	0.09 (0.06-0.12)	0 (0-0)	0.04 (0.03-0.07)	-2.35 (-3.08--1.61)
Morocco	31 (18-46)	0.38 (0.22-0.56)	14 (8-21)	0.15 (0.09-0.22)	-3.7 (-3.91--3.49)

Mozambique	23 (12-37)	0.55 (0.3-0.92)	60 (30-99)	0.61 (0.31-1)	1 (0.71-1.29)
Myanmar	200 (115-347)	1.56 (0.9-2.71)	52 (30-92)	0.36 (0.2-0.63)	-5.55 (-5.79--5.31)
Namibia	1 (1-2)	0.28 (0.14-0.43)	1 (1-2)	0.18 (0.1-0.3)	-1.17 (-1.63--0.71)
Nauru	0 (0-0)	1.23 (0.7-2.08)	0 (0-0)	0.81 (0.39-1.35)	-1.49 (-1.95--1.02)
Nepal	2 (1-3)	0.03 (0.02-0.05)	1 (0-1)	0.01 (0-0.01)	-5.25 (-5.76--4.73)
Netherlands	2 (1-2)	0.05 (0.04-0.06)	0 (0-0)	0.01 (0-0.01)	-8.18 (-8.63--7.72)
New Zealand	0 (0-0)	0.04 (0.03-0.05)	0 (0-0)	0.01 (0.01-0.01)	-6.25 (-6.83--5.67)
Nicaragua	3 (2-4)	0.21 (0.16-0.29)	2 (1-3)	0.08 (0.05-0.13)	-3.51 (-3.93--3.1)
Niger	28 (17-44)	1.15 (0.68-1.8)	70 (35-117)	0.9 (0.46-1.52)	-0.77 (-0.92--0.63)
Nigeria	247 (152-426)	0.88 (0.54-1.51)	409 (208-791)	0.56 (0.29-1.09)	-1.39 (-1.56--1.22)
Niue	0 (0-0)	0.72 (0.43-1.17)	0 (0-0)	0.39 (0.22-0.67)	-2.3 (-2.42--2.18)
North Macedonia	0 (0-0)	0.06 (0.04-0.09)	0 (0-0)	0.02 (0.01-0.04)	-4.17 (-4.59--3.75)
Northern Mariana Islands	0 (0-0)	0.37 (0.22-0.57)	0 (0-0)	0.18 (0.12-0.26)	-2.67 (-3.02--2.32)
Norway	0 (0-0)	0.03 (0.02-0.04)	0 (0-0)	0.01 (0-0.01)	-5.83 (-6.25--5.42)
Oman	1 (0-1)	0.1 (0.06-0.17)	0 (0-1)	0.04 (0.02-0.06)	-2.31 (-2.8--1.83)
Pakistan	116 (75-172)	0.32 (0.21-0.48)	159 (97-243)	0.22 (0.13-0.33)	-1.74 (-2--1.47)
Palau	0 (0-0)	0.49 (0.27-0.81)	0 (0-0)	0.35 (0.21-0.55)	-0.92 (-1.02--0.82)
Palestine	1 (0-2)	0.15 (0.06-0.32)	1 (1-2)	0.07 (0.04-0.1)	-2.79 (-3.28--2.29)
Panama	1 (1-1)	0.12 (0.09-0.15)	0 (0-1)	0.04 (0.02-0.05)	-3.88 (-4.31--3.45)
Papua New Guinea	13 (9-19)	1.02 (0.68-1.49)	21 (13-35)	0.73 (0.44-1.19)	-1.2 (-1.29--1.1)

Paraguay	2 (1-3)	0.16 (0.12-0.22)	2 (1-2)	0.08 (0.05-0.12)	-3.09 (-3.35--2.84)
Peru	39 (30-51)	0.55 (0.42-0.73)	12 (7-18)	0.14 (0.09-0.21)	-5 (-5.42--4.59)
Philippines	160 (134-219)	0.78 (0.65-1.07)	137 (108-200)	0.43 (0.34-0.62)	-1.33 (-1.72--0.94)
Poland	9 (8-10)	0.1 (0.09-0.11)	3 (2-3)	0.04 (0.04-0.05)	-1.97 (-2.44--1.51)
Portugal	2 (2-3)	0.09 (0.07-0.11)	0 (0-0)	0.01 (0.01-0.01)	-8.99 (-9.62--8.37)
Puerto Rico	1 (1-1)	0.09 (0.07-0.12)	0 (0-0)	0.02 (0.01-0.03)	-7.3 (-7.86--6.73)
Qatar	0 (0-0)	0.05 (0.03-0.09)	0 (0-0)	0.02 (0.01-0.03)	-3.07 (-3.49--2.64)
Republic of Korea	21 (16-28)	0.16 (0.12-0.22)	1 (0-1)	0.01 (0-0.01)	-9.79 (-10.71--8.86)
Republic of Moldova	3 (2-4)	0.28 (0.21-0.35)	1 (1-1)	0.13 (0.09-0.17)	-2.57 (-2.87--2.26)
Romania	11 (9-14)	0.19 (0.15-0.23)	2 (1-3)	0.06 (0.04-0.08)	-4.54 (-4.84--4.24)
Russian Federation	54 (49-61)	0.17 (0.15-0.19)	20 (16-25)	0.09 (0.07-0.11)	-3.13 (-3.96--2.3)
Rwanda	37 (23-58)	1.62 (1.02-2.56)	25 (14-42)	0.6 (0.33-1.01)	-4.37 (-4.73--4.01)
Saint Kitts and Nevis	0 (0-0)	0.13 (0.1-0.17)	0 (0-0)	0.05 (0.03-0.08)	-1.7 (-2.49--0.9)
Saint Lucia	0 (0-0)	0.17 (0.13-0.22)	0 (0-0)	0.09 (0.06-0.12)	-2.71 (-3.02--2.4)
Saint Vincent and the Grenadines	0 (0-0)	0.25 (0.19-0.33)	0 (0-0)	0.14 (0.1-0.19)	-2.78 (-3.24--2.31)
Samoa	0 (0-1)	0.71 (0.4-1.16)	0 (0-0)	0.39 (0.23-0.59)	-2.03 (-2.12--1.95)
San Marino	0 (0-0)	0.01 (0.01-0.02)	0 (0-0)	0.01 (0-0.01)	-2 (-2.43--1.57)
Sao Tome and Principe	0 (0-0)	0.43 (0.21-0.73)	0 (0-0)	0.26 (0.15-0.48)	-2.53 (-3.43--1.62)
Saudi Arabia	6 (3-10)	0.12 (0.06-0.2)	3 (2-5)	0.04 (0.02-0.06)	-3.27 (-3.54--3)
Senegal	22 (12-35)	0.92 (0.51-1.43)	30 (15-49)	0.59 (0.3-0.98)	-1.26 (-1.53--0.99)

Serbia	2 (1-2)	0.07 (0.05-0.1)	0 (0-0)	0.02 (0.01-0.03)	-5.18 (-5.47--4.89)
Seychelles	0 (0-0)	0.33 (0.21-0.46)	0 (0-0)	0.18 (0.11-0.27)	-2.03 (-2.61--1.44)
Sierra Leone	8 (5-13)	0.77 (0.44-1.25)	21 (11-32)	0.75 (0.4-1.18)	0.58 (0.36-0.8)
Singapore	0 (0-1)	0.05 (0.04-0.07)	0 (0-0)	0.01 (0.01-0.01)	-7.24 (-7.71--6.78)
Slovakia	1 (1-1)	0.08 (0.06-0.1)	0 (0-1)	0.04 (0.03-0.06)	-2.02 (-2.21--1.82)
Slovenia	0 (0-0)	0.07 (0.05-0.09)	0 (0-0)	0.02 (0.02-0.03)	-4.97 (-5.73--4.21)
Solomon Islands	2 (1-2)	1.32 (0.73-2.12)	2 (1-3)	0.88 (0.58-1.34)	-1.24 (-1.32--1.15)
Somalia	18 (8-31)	0.77 (0.34-1.37)	53 (25-98)	0.78 (0.37-1.43)	-0.59 (-1.11--0.07)
South Africa	20 (13-27)	0.17 (0.11-0.23)	11 (8-16)	0.08 (0.05-0.11)	-1.69 (-2.87--0.49)
South Sudan	10 (5-20)	0.51 (0.23-1.02)	12 (6-23)	0.37 (0.19-0.7)	-1.46 (-1.72--1.19)
Spain	8 (6-10)	0.08 (0.06-0.1)	0 (0-1)	0.01 (0-0.01)	-9.18 (-9.63--8.73)
Sri Lanka	7 (3-9)	0.13 (0.06-0.18)	1 (1-2)	0.02 (0.01-0.04)	-5.72 (-6.26--5.17)
Sudan	27 (13-44)	0.41 (0.2-0.69)	28 (14-49)	0.2 (0.1-0.37)	-2.15 (-2.25--2.06)
Suriname	1 (0-1)	0.47 (0.27-0.64)	0 (0-0)	0.2 (0.14-0.28)	-3.6 (-4.01--3.19)
Sweden	1 (1-1)	0.06 (0.04-0.07)	0 (0-0)	0.02 (0.01-0.02)	-3.59 (-3.79--3.39)
Switzerland	0 (0-0)	0.03 (0.02-0.04)	0 (0-0)	0.01 (0-0.01)	-6.17 (-6.48--5.87)
Syrian Arab Republic	10 (6-15)	0.22 (0.15-0.36)	4 (2-6)	0.07 (0.04-0.12)	-4.1 (-4.57--3.63)
Taiwan (Province of China)	8 (6-9)	0.13 (0.11-0.16)	1 (1-1)	0.02 (0.01-0.03)	-7.53 (-8.08--6.99)
Tajikistan	7 (5-10)	0.4 (0.3-0.59)	7 (4-12)	0.25 (0.16-0.43)	-3.63 (-4.43--2.83)
Thailand	23 (12-36)	0.13 (0.07-0.2)	5 (3-8)	0.04 (0.02-0.06)	-6.02 (-7.09--4.93)

Timor-Leste	2 (1-3)	0.97 (0.44-1.49)	2 (1-3)	0.45 (0.25-0.66)	-3.42 (-4.11--2.73)
Togo	9 (6-13)	0.76 (0.5-1.08)	14 (8-21)	0.57 (0.33-0.86)	-0.51 (-0.69--0.34)
Tokelau	0 (0-0)	0.78 (0.43-1.26)	0 (0-0)	0.32 (0.19-0.49)	-3.19 (-3.27--3.1)
Tonga	0 (0-0)	0.51 (0.3-0.75)	0 (0-0)	0.4 (0.25-0.61)	-0.69 (-0.94--0.44)
Trinidad and Tobago	1 (1-1)	0.21 (0.16-0.26)	0 (0-0)	0.1 (0.06-0.15)	-2.42 (-3.05--1.78)
Tunisia	5 (3-10)	0.2 (0.1-0.36)	2 (1-4)	0.08 (0.05-0.15)	-3.01 (-3.14--2.88)
Turkmenistan	4 (3-5)	0.32 (0.25-0.41)	4 (3-6)	0.32 (0.22-0.46)	-0.74 (-1.33--0.15)
Tuvalu	0 (0-0)	1.36 (0.71-2.25)	0 (0-0)	0.57 (0.32-0.88)	-2.75 (-2.9--2.61)
Türkiye	33 (22-51)	0.18 (0.11-0.27)	8 (6-12)	0.04 (0.03-0.06)	-5.31 (-5.88--4.73)
Uganda	31 (17-51)	0.55 (0.31-0.91)	71 (40-115)	0.49 (0.28-0.8)	-1.32 (-1.82--0.82)
Ukraine	18 (15-22)	0.17 (0.14-0.2)	14 (10-20)	0.21 (0.15-0.31)	-0.01 (-0.91-0.89)
United Arab Emirates	0 (0-0)	0.03 (0.02-0.05)	0 (0-0)	0.01 (0.01-0.02)	-3.52 (-3.7--3.33)
United Kingdom	8 (8-10)	0.07 (0.07-0.08)	2 (2-3)	0.02 (0.02-0.03)	-5.57 (-5.92--5.22)
United Republic of Tanzania	33 (21-48)	0.38 (0.24-0.57)	51 (27-84)	0.28 (0.15-0.46)	-1.04 (-1.3--0.77)
United States of America	20 (18-21)	0.04 (0.03-0.04)	7 (6-8)	0.01 (0.01-0.01)	-4.57 (-4.96--4.17)
United States Virgin Islands	0 (0-0)	0.13 (0.09-0.19)	0 (0-0)	0.04 (0.02-0.06)	-6.25 (-7.29--5.19)
Uruguay	1 (0-1)	0.07 (0.06-0.09)	0 (0-0)	0.03 (0.02-0.04)	-3.34 (-3.8--2.87)
Uzbekistan	20 (15-24)	0.31 (0.24-0.38)	22 (16-30)	0.24 (0.18-0.33)	-1.95 (-2.66--1.24)
Vanuatu	1 (0-1)	1.23 (0.69-1.94)	1 (1-1)	0.97 (0.58-1.5)	-1.42 (-1.65--1.18)
Venezuela (Bolivarian Republic of)	10 (7-13)	0.16 (0.12-0.21)	6 (4-9)	0.09 (0.06-0.13)	-2.44 (-3.09--1.79)

Viet Nam	5 (3-8)	0.02 (0.02-0.04)	2 (1-3)	0.01 (0-0.01)	-4.31 (-4.77--3.85)
Yemen	16 (7-28)	0.37 (0.16-0.67)	24 (13-40)	0.24 (0.13-0.39)	-1.72 (-1.96--1.49)
Zambia	25 (16-37)	0.89 (0.57-1.34)	28 (16-44)	0.46 (0.27-0.72)	-2.71 (-3.16--2.26)
Zimbabwe	18 (10-31)	0.5 (0.29-0.86)	35 (19-58)	0.71 (0.38-1.19)	1.63 (1.33-1.94)

Supplementary Table 5： Comparison of Forecasting Performance Metrics Between ES and ARIMA Models

	AIC	BIC	ME	RMSE	MAE	MASE	ACF1
ARIMA	-68.93	-63.46	-0.00277	0.058103	0.038859	0.227785	-0.1384
ES	734.627	741.633	-6619.16	32096.99	25337.68	0.765643	-0.193

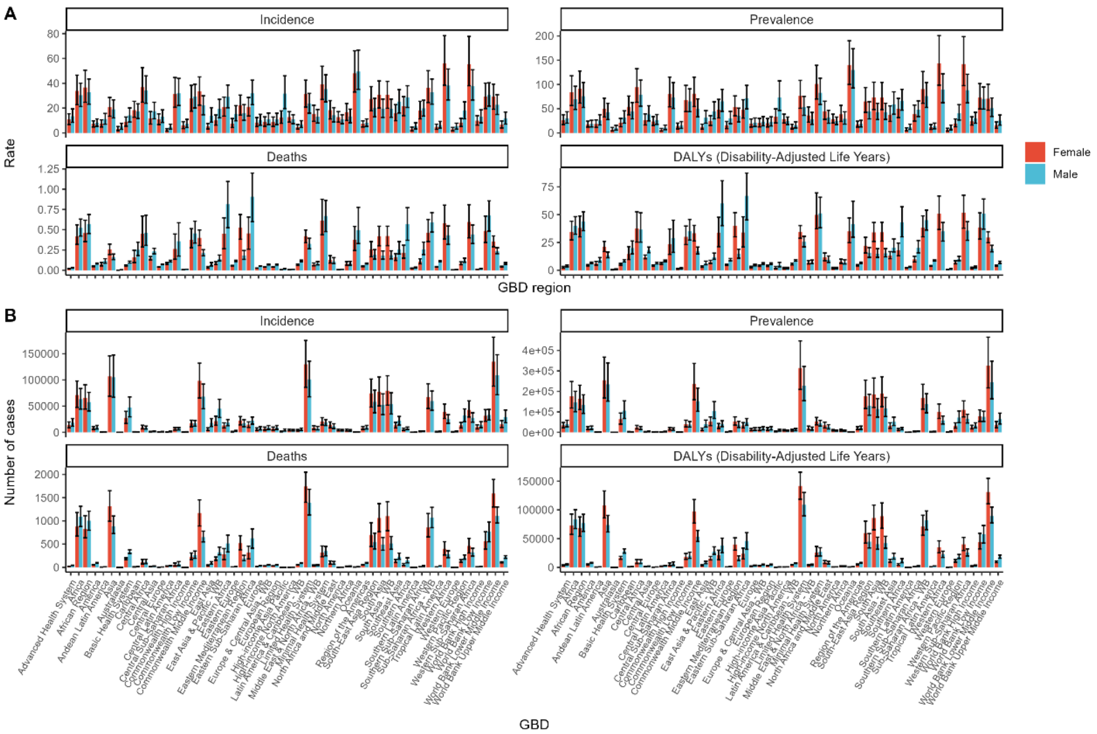

Supplementary1: Sex-specific burden of gastrointestinal ulcers among individuals aged 10–24 years across GBD regions, showing rates and case numbers with 95% uncertainty intervals.

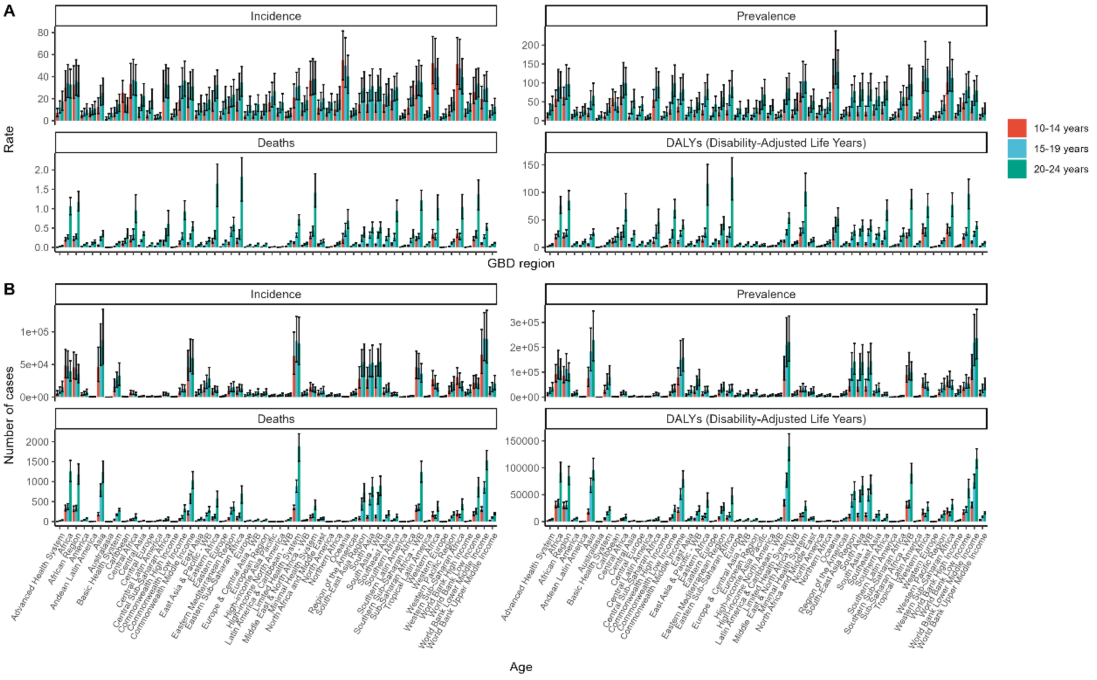

Supplementary2: Age-specific burden of gastrointestinal ulcers among individuals aged 10–24 years across GBD regions, showing rates and case numbers with 95% uncertainty intervals.

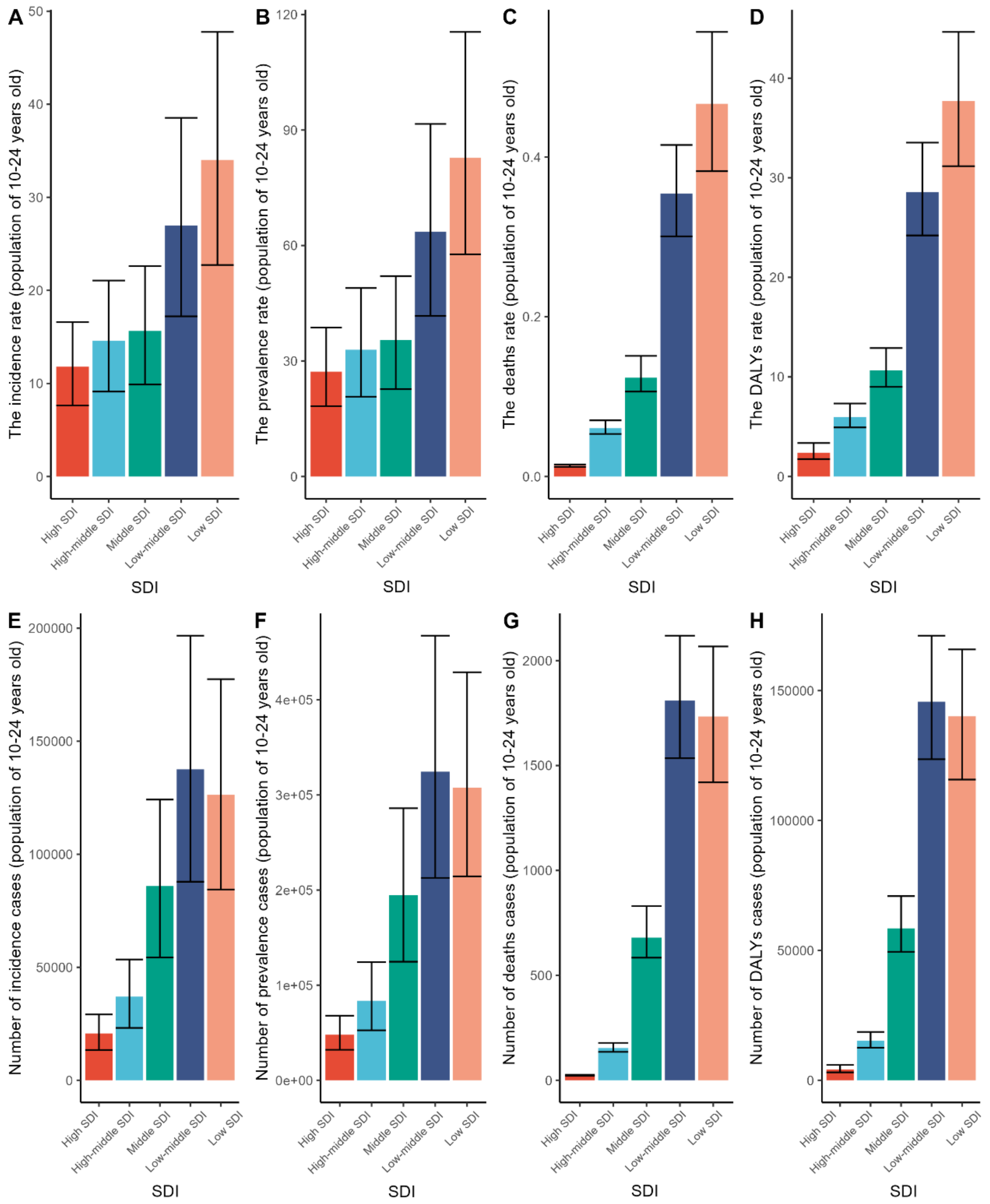

Supplementary3: Burden of gastrointestinal ulcers among individuals aged 10–24 years across SDI levels, with 95% uncertainty intervals.

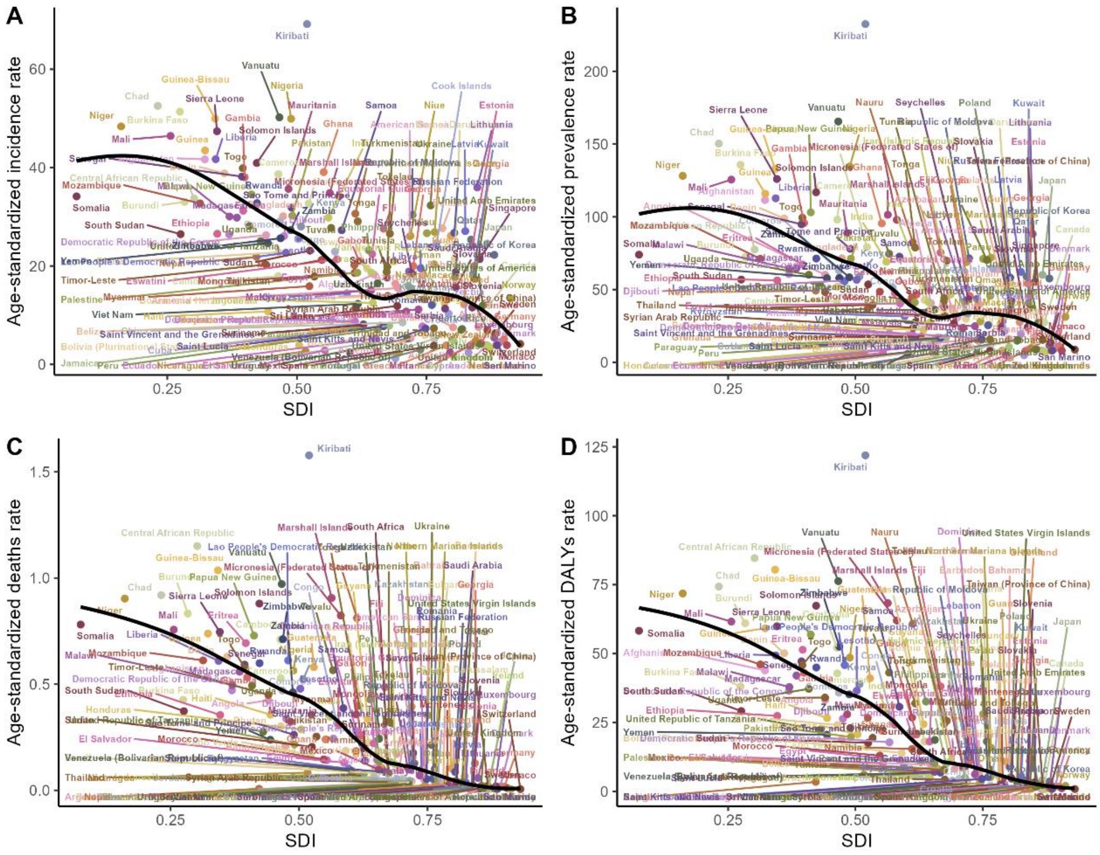

Supplementary4: Association between SDI and age-standardized rates of gastrointestinal ulcers (incidence, prevalence, deaths, and DALYs) among individuals aged 10–24 years across countries.

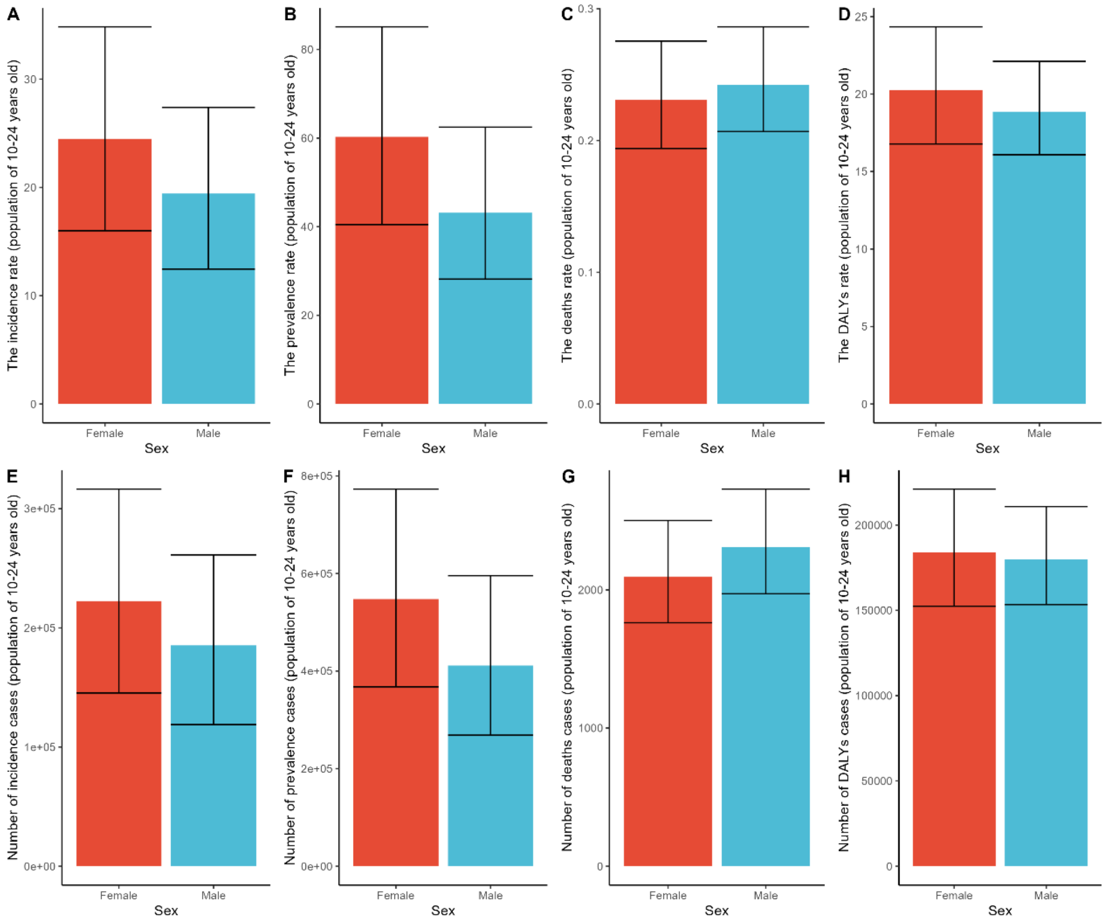

Supplementary5: Comparison of gastrointestinal ulcer burden between females and males aged 10–24 years, showing rates and case numbers with 95% uncertainty intervals.

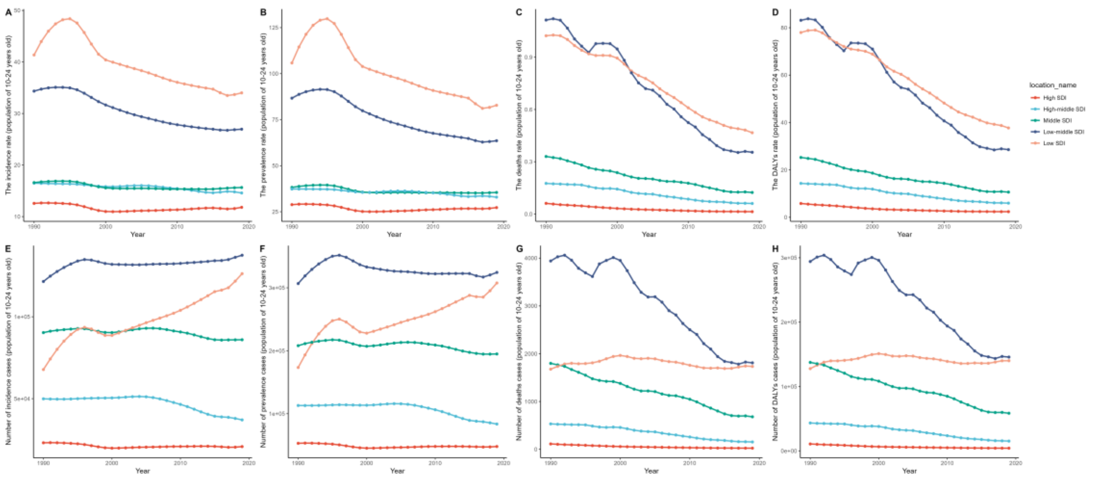

Supplementary6: Trends in gastrointestinal ulcer burden among individuals aged 10–24 years from 1990 to 2019, stratified by World Bank income level, showing age-standardized rates and case numbers with 95% uncertainty intervals.

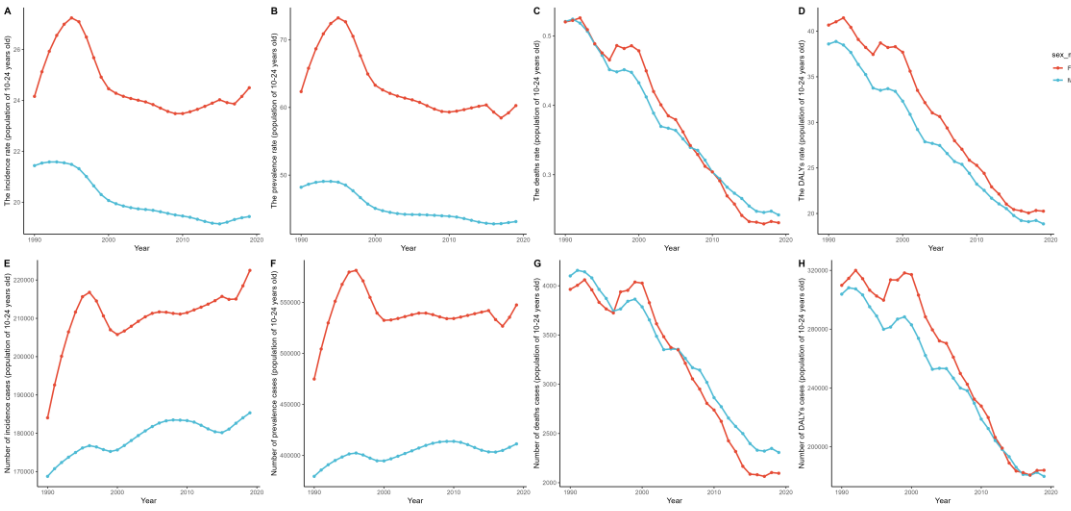

Supplementary7: rends in gastrointestinal ulcer burden among females and males aged 10–24 years from 1990 to 2019, showing age-standardized rates and case numbers with 95% uncertainty intervals..
